# Twenty-Five Novel Loci for Carotid Intima-Media Thickness: A Genome-Wide Association Study in >45 000 Individuals and Meta-Analysis of >100 000 Individuals

**DOI:** 10.1161/ATVBAHA.121.317007

**Published:** 2021-12-02

**Authors:** Ming Wai Yeung, Siqi Wang, Yordi J. van de Vegte, Oleg Borisov, Jessica van Setten, Harold Snieder, Niek Verweij, M. Abdullah Said, Pim van der Harst

**Affiliations:** Department of Cardiology (M.W.Y., S.W., Y.J.v.d.V., N.V., M.A.S., P.v.d.H.), University of Groningen, University Medical Center Groningen, the Netherlands.; Department of Epidemiology (S.W., H.S.), University of Groningen, University Medical Center Groningen, the Netherlands.; Division of Heart & Lungs, Department of Cardiology, University Medical Center Utrecht, University of Utrecht, the Netherlands (M.W.Y., J.v.S., P.v.d.H.).; Institute for Genomic Statistics and Bioinformatics, University Hospital Bonn, Germany (O.B.).

**Keywords:** carotid intima-media thickness, genetics, genome-wide association study, meta, analysis, population genetics

## Abstract

Carotid artery intima-media thickness (cIMT) is a widely accepted marker of subclinical atherosclerosis. Twenty susceptibility loci for cIMT were previously identified and the identification of additional susceptibility loci furthers our knowledge on the genetic architecture underlying atherosclerosis.

**Approach and Results:**

We performed 3 genome-wide association studies in 45 185 participants from the UK Biobank study who underwent cIMT measurements and had data on minimum, mean, and maximum thickness. We replicated 15 known loci and identified 20 novel loci associated with cIMT at *P*<5×10^−8^. Seven novel loci (*ZNF385D*, AD*AMTS9*, *EDNRA*, *HAND2*, *MYOCD*, *ITCH/EDEM2/MMP24*, and *MRTFA*) were identified in all 3 phenotypes. An additional new locus (*LOXL1*) was identified in the meta-analysis of the 3 phenotypes. Sex interaction analysis revealed sex differences in 7 loci including a novel locus (*SYNE3*) in males. Meta-analysis of UK Biobank data with a previous meta-analysis led to identification of three novel loci (*APOB, FIP1L1, and LOXL4*). Transcriptome-wide association analyses implicated additional genes *ARHGAP42*, *NDRG4*, and *KANK2*. Gene set analysis showed an enrichment in extracellular organization and the PDGF (platelet-derived growth factor) signaling pathway. We found positive genetic correlations of cIMT with coronary artery disease *r*_*g*_=0.21 (*P*=1.4×10^-7^), peripheral artery disease *r*_*g*_=0.45 (*P*=5.3×10^-5^), and systolic blood pressure *r*_*g*_=0.30 (*P*=4.0×10^-18^). A negative genetic correlation between average of maximum cIMT and high-density lipoprotein was found *r*_*g*_=−0.12 (*P*=7.0×10^-4^).

**Conclusions:**

Genome-wide association meta-analyses in >100 000 individuals identified 25 novel loci associated with cIMT providing insights into genes and tissue-specific regulatory mechanisms of proatherosclerotic processes. We found evidence for shared biological mechanisms with cardiovascular diseases.

**Highlights**

- Leveraging data from over 45 000 UK Biobank participants, we discovered 22 novel genetic loci and replicated 15 known loci associated with carotid intima-media thickness from genome-wide association study.
- Meta-analysis of >100k individuals combining UK Biobank data and a previous meta-analysis yielded three additional novel loci.
- Interaction analysis revealed presence of sex-specific effects in one of the novel loci and six previously reported loci.
- Expression analyses revealed many prioritized genes enriched in endothelial cells and smooth muscle cells.
- Positive genetic correlations between carotid intima-media thickness and respectively peripheral artery disease, coronary artery disease, ischemic stroke and systolic blood pressure were observed; A negative genetic correlation was found between carotid intima-media thickness and high-density lipoprotein cholesterol.

**See accompanying editorial on page 502**

Cardiovascular disease is a leading cause of death worldwide with atherosclerosis as the underlying cause of most cases.^1^ Atherosclerosis is characterized by the accumulation of lipids in the subintimal space of medium-sized and large arteries. A fibrous cap is formed on the atherosclerotic plaque due to the proliferation of smooth muscle cells and matrix deposition.^2^ Plaques can gradually impede the blood flow, eventually leading to ischemia of end-organ tissues. Rupture of plaques due to unstable, thin caps and subsequent thrombosis can lead to abrupt occlusion of the artery, causing a stop of the blood flow and thereby ischemia and necrosis of the tissue following the occlusion.^3^ This process is the underlying pathophysiological mechanism of major cardiovascular events, such as myocardial infarction^4^ and ischemic stroke,^5^ which are both leading causes of mortality and disability in developed countries.^6^

Due to the long preceding phase of atherosclerosis, it can take decades before the clinical presentation of symptoms. An early detection of atherosclerotic plaque formation may aid in the risk stratification and identification of individuals at high risk of atherosclerotic cardiovascular diseases, such as myocardial infarction, coronary artery disease (CAD), and peripheral artery disease (PAD). Noninvasive imaging techniques such as ultrasound measurement of the carotid artery intima-media thickness (cIMT) can detect subclinical atherosclerosis and is widely accepted as an index of atherosclerosis severity.^7^ Increased cIMT has been associated with higher risk of cardiovascular diseases.^8,9^ Twin studies suggest a substantial genetic component in cIMT,^10^ and previous genome-wide association studies (GWAS) of cIMT have identified 20 genetic loci linked to known genes as well as novel candidate genes associated with atherosclerosis.^11–13^ In the current study, we aimed to identify additional genetic loci to further our knowledge on the genetic architecture of increased cIMT using a large population-based study.

## Methods

Please see the Major Resources Table in the Supplemental Material. The data that support the findings of this study are available from the corresponding author upon reasonable request.

### Study Population

The UK Biobank study is a population-based prospective cohort in the United Kingdom in which ≈500 000 individuals aged between 40 and 69 years were included between 2006 and 2010. All participants have given informed consent for this study. The UK Biobank has ethical approval from North West–Haydock Research Ethics Committee (REC reference: 16/NW/0274). Details of the UK Biobank study have been described in detail previously.^14^ This research has been conducted using the UK Biobank Resource under Application Number 15031.

### cIMT Measurements

At the time of analyses, cIMT measurements were available for 46 705 participants who had completed the second follow-up visit for the UK Biobank. The cIMT measurements were derived automatically from the 2-dimensional carotid scan using a CardioHealth Station (Panasonic Biomedical Sales Europe BV, Leicestershire, United Kingdom). A total of 4 cIMT measurements were taken, namely at 120° and 150° for the right carotid and 210° and 240° for the left carotid artery. For each angle, the minimum, mean and maximum values were recorded.^15^

Quality control of cIMT measurements was performed by the UK Biobank, validated both internally and externally using a set of predefined criteria.^15,16^ cIMT measurements that did not pass the quality control, either with cIMT values of zero or as indicated by the corresponding quality control flags (field IDs 22682, 22683, 22684, 22685) were excluded from the analyses. Measurements collected at the second follow-up visit (imaging visit) were considered for the current analysis. Average values of the cIMT of the four angles were taken as the final cIMT measurements, namely average of minimum cIMT (cIMT_min_), average of mean cIMT (cIMT_mean_), and average of maximum cIMT (cIMT_max_).

### Genotyping and Imputation

Genotype data for 488 377 participants were available in the UK Biobank study. Two custom Affymetrix Axiom (UK Biobank Lung Exome Variant Evaluation or UK Biobank) genotyping arrays with >95% common content were used. Imputation was performed using Haplotype Reference Consortium as the primary reference panel with addition of merged UK10K and 1000 Genome phase 3 reference panels. Quality control of samples and variants, as well as of the imputation was performed by the Wellcome Trust Centre for Human Genetics.^17^ The current study was performed on the imputed data supplied by the UK Biobank. Individuals with overall missingness >5% or excessive heterozygosity, and individuals whose genetically inferred sex did not match with the reported sex, were excluded from the analyses. Additionally, variants with a minor allele frequency <0.5% or an INFO-score (imputation information score) smaller than 0.3 were excluded.

### Genome-Wide Association Studies

A transethnic GWAS (demographics shown in Table S1 in the Supplemental Material) was performed on 3 averaged cIMT measurements; cIMT_min_, cIMT_mean_, and cIMT_max_, using BOLT-LMM v2.3.1.^18^ A linear mixed model was fitted for each of the inverse rank normalized cIMT measurements. GWAS analyses were adjusted for age at the time of the imaging visit, sex, genotyping array, and the first 30 principal components (provided by UK Biobank) to adjust for population stratification. Individuals with missing information on any of the covariates were excluded from the GWAS analyses. To obtain a set of independent single nucleotide polymorphisms (SNPs) associated with cIMT phenotypes, clumping was performed on variants (lead variants) that passed the genome-wide significance threshold of *P*<5×10^-8^ based on linkage disequilibrium (LD) r^2^>0.005 and 2.5-Mb distance using the clumping procedure in PLINK 1.9.^19^ A locus was defined as a 1-Mb region surrounding the most significant variant. The nearest protein-coding gene and any additional gene within 10 kb of each locus’ lead variant were annotated to the locus.

### Statistical Fine-Mapping

To further refine the signals detected in GWAS, we applied statistical fine-mapping across a 1-MB region around the lead variant for each of the loci identified to prioritize putative causal variants. Bayesian fine-mapping was performed on summary statistics of cIMT_mean_ using FINEMAP (v1.4).^20^ A shotgun stochastic search method was used to produce 95% credible sets under the assumption of several causal variants (*k*) from 1 up to 5, each with estimated posterior probabilities which jointly summed up to 1. We considered the variants in the top causal configuration under *k* with the highest posterior probabilities as the likely causal variants. For each likely causal variant, we searched for coding variants in high LD (R^2^ > 0.8) with dbNSFP (database for nonsynonymous SNPs’ functional predictions, v.4.2).^21^

### Meta-Analysis of cIMT Measurements

Meta-analysis of the UK Biobank and the largest GWAS meta-analysis of cIMT previously published by the CHARGE (Cohorts for Heart and Aging Research in Genomic Epidemiology)/UCLEB (UCL-LSHTM-Edinburgh-Bristol) consortia (71 128 European participants from 31 studies)^12^ was performed for cIMT_max_ using Multi-Trait Analysis of GWAS (MTAG)^22^; MTAG is a tool for analysis of multiple GWAS summary statistics which applies a generalized inverse-variance-weighted meta-analysis that allows for sample overlap. Additionally, a second meta-analysis of the 3 cIMT measurements within the UK Biobank was performed with MTAG. All MTAG analyses were performed on common variants (minor allele frequency >0.01) with an INFO-score >0.3 under the assumption of perfect genetic correlation and equal heritability (with the options --perfect_gencov and --equal_h2).

### Sex Interaction Analysis

To examine if there are sex-specific differences in the genetic loci associated with cIMT, we performed sex-stratified GWAS on all 3 cIMT traits. We systematically tested the difference between the effect size estimates obtained from the stratified GWAS. A *P* value (*P*_sexdiff_) was obtained using the following test statistics^23^:


$$t_{sex}=\frac{Beta_{male}-Beta_{female}}{\sqrt{SE_{male}^{2}+SE_{female}^{2}-2r_{sex}\cdot SE_{male}\cdot SE_{female}}}$$

where *r*_*sex*_ was the Spearman rank correlation coefficient between sexes across all variants included in the sex-stratified GWAS. We examined the sex-specific effects on loci either identified in the main GWAS or sex-stratified GWAS, and a locus was considered to demonstrate sex difference if *P*_sexdiff_ passed Bonferroni-corrected threshold (=0.05/number of loci tested).

### Transcriptome-Wide Association Study

To prioritize genes identified in GWAS, we incorporated expression quantitative trait loci data and performed a transcriptome-wide association study (TWAS). Tissue-specific expression levels of the genes were predicted using a model trained on an external reference panel with both genotype and expression data available; associations between the predicted expression levels and cIMT phenotypes were then tested on a genic level. We performed the TWAS using the Unified Test for Molecular SignaTures method with imputation models pretrained on data from the Genotype-Tissue Expression project.^24,25^ Briefly, gene expression levels were imputed by training a multivariate lasso regression model taking all available tissues into considerations (separate penalty terms for within-tissue and cross-tissue effects which allow different effect directions in different tissues). Imputed gene expressions were then used for the association tests with the cIMT traits (TWAS). Association analyses were performed on three arterial tissues, namely aorta, coronary and tibial, to obtain tissue-specific result (arterial TWAS). Given that the relatively smaller sample size for arterial tissues may restrict the power to detect potential associations, a joint test combining the test statistics of 44 tissues was calculated using the Generalized Berk-Jones test (cross-tissue TWAS).^24^

### Multi-Marker Analysis for Genomic Annotation

In addition to TWAS, we applied Multi-Marker Analysis for Genomic Annotation (MAGMA) tool to conduct gene-based tests as well as a gene set analysis.^26^ Briefly, gene-based test with summary statistics involves computation of a gene-level test statistics by combining that of all variants within a gene, after which gene-based *P* was derived from an approximate sampling distribution estimated from a reference dataset. The gene-based statistics were then aggregated to predefined sets for association tests with cIMT traits, with LD between genes corrected by computing gene correlation matrix using the reference dataset. MAGMA analysis was done via the platform Functional Mapping and Annotation of GWAS v1.3.6a with MAGMA v1.08a, using default settings. Gene-based analyses were done with an SNP-wide mean model and the gene set analysis was performed using 15 496 gene sets (5500 curated gene sets and 9996 Gene Ontology terms respectively) from MsigDB v7.0.^27,28^ We applied Bonferroni corrections to assess the statistical significance of results from all gene-based and gene set analyses.

### Heritability Estimation of cIMT and Genetic Correlation With Cardiovascular Diseases and Risk Factors

The heritability of cIMT measurements was estimated using the restricted maximum likelihood algorithm from the BOLT-LMM software. The proportion of additive variance explained by the lead variants was estimated by fitting a multivariable linear regression model on the cIMT measurements, assuming an additive genetic model. To investigate the shared genetic component between cIMT and vascular diseases namely, abdominal aortic aneurysm (AAA), CAD, stroke, and PAD as well as cardiovascular diseases (CVD) risk factors, we estimated genetic correlations between cIMT phenotypes and the diseases/risk factors both within our data and with the addition of external cohorts using linkage disequilibrium score regression (LDSC) v1.0.1.^29^ The CVD risk factors investigated in the current study include diastolic blood pressure (DBP), systolic blood pressure (SBP), HDL-C (high-density lipoprotein cholesterol), LDL-C (low-density lipoprotein cholesterol), total cholesterol, and triglyceride levels. Ascertainment of AAA, CAD, ischemic stroke, and PAD in the UK Biobank was based on a combination of hospital inpatient and self-reported records during an interview with a trained nurse (Table S2 in the Supplemental Material). Measurements of CVD risk factors in the UK Biobank are described in the supplement (Supplementary Method). Genetic associations of the cardiovascular disease were done within the subset of unrelated UK Biobank participants that was not included in the cIMT GWAS. With regards to the analysis using external cohorts, we used summary statistics data from the CARDIoGRAMplusC4D (Coronary Artery Disease Genome-Wide Replication and Meta-Analysis Plus the Coronary Artery Disease Genetics) consortium, a meta-analysis with 60 801 CAD cases and 123 504 controls to estimate the genetic correlation between cIMT and CAD.^30^ Summary statistics of the transethnic meta-analysis MEGASTROKE collaboration (60 341 cases; 454 450 controls) were used for the genetic correlation between cIMT and stroke including its subtypes.^31^ The summary statistics from transethnic analysis on the GERA (Genetic Epidemiology Research on Adult Health and Aging) cohort consisting of 99 785 individuals were used for the genetic correlation with blood pressure traits.^32^ For the analysis with lipid traits, we used the summary statistics from the meta-analysis (joint analysis of Metabochip and GWAS with a total of 188 577 individuals) from the GLGC (Global Lipids Genetics Consortium).^33^ We additionally compared the cIMT loci with the loci identified for these cardiovascular diseases using Functional Mapping and Annotation of GWAS, which searched the GWAS Catalog (version e96_r2019-09-24) for variants in high LD (R^2^>0.6) with lead variants in our GWAS.^34^

## Results

### Study Population Characteristics

Out of 502 493 UK Biobank participants, we included 45 185 individuals with available cIMT measurements that passed the quality control and had available genetic data (Figure S1 in the Supplemental Material). Baseline characteristics of the study population are shown in Table S1 in the Supplemental Material. This population consisted of a majority of White participants (96.6%) along with participants of Asian descent (1.4%), African descent (0.6%), of a mixed descent (0.5%), and of other/unknown ethnic background (0.6%). Overall, the mean (SD) cIMT_min_ was 0.58 (0.11) mm, the mean cIMT_mean_ was 0.69 (0.13) mm, and the mean cIMT_max_ was 0.80 (0.15) mm. Smaller cIMT measurements were observed in women compared with men with cIMT_min_ 0.57 (0.10) versus 0.60 (0.12), cIMT_mean_ 0.67 (0.11) versus 0.71 (0.14), and cIMT_max_ 0.77 (0.13) versus 0.83 (0.16). The cIMT measurements were strongly correlated (r^2^ 0.86-0.96; Figure S2 in the Supplemental Material).

### Novel Loci Associated With cIMT

A total of 11 247 984 variants were tested in the current analyses. A total of 2837 variants in 30 loci were associated with cIMT_min_, 2781 variants in 30 loci with cIMT_mean_, and 1789 variants in 25 loci with cIMT_max_. A summary of all loci that were identified can be found in Table 1. Among these, a total of 20 loci have not been reported in previous studies. Moreover, all loci except 5q31.3 (*NR3C1*) and 12q21.31 (*RASSF9*) remained significantly associated with the cIMT phenotypes at a stringent Bonferroni adjusted *P*<1.67×10^-8^ (5×10^-8^/3). Replication of loci identified in the current study in a previous large meta-analysis of the CHARGE consortium^12^ can be found in Table S3 in the Supplemental Material. Of the 20 novel loci identified in the current study, 15 lead variants had *P*<0.05 in the external cohort with consistent direction of effect. Conversely, we looked up previously reported associations in the current result. Association results of the five previously reported loci that were not replicated in the current analysis are provided in Table S4 in the Supplemental Material.

**Table 1.**
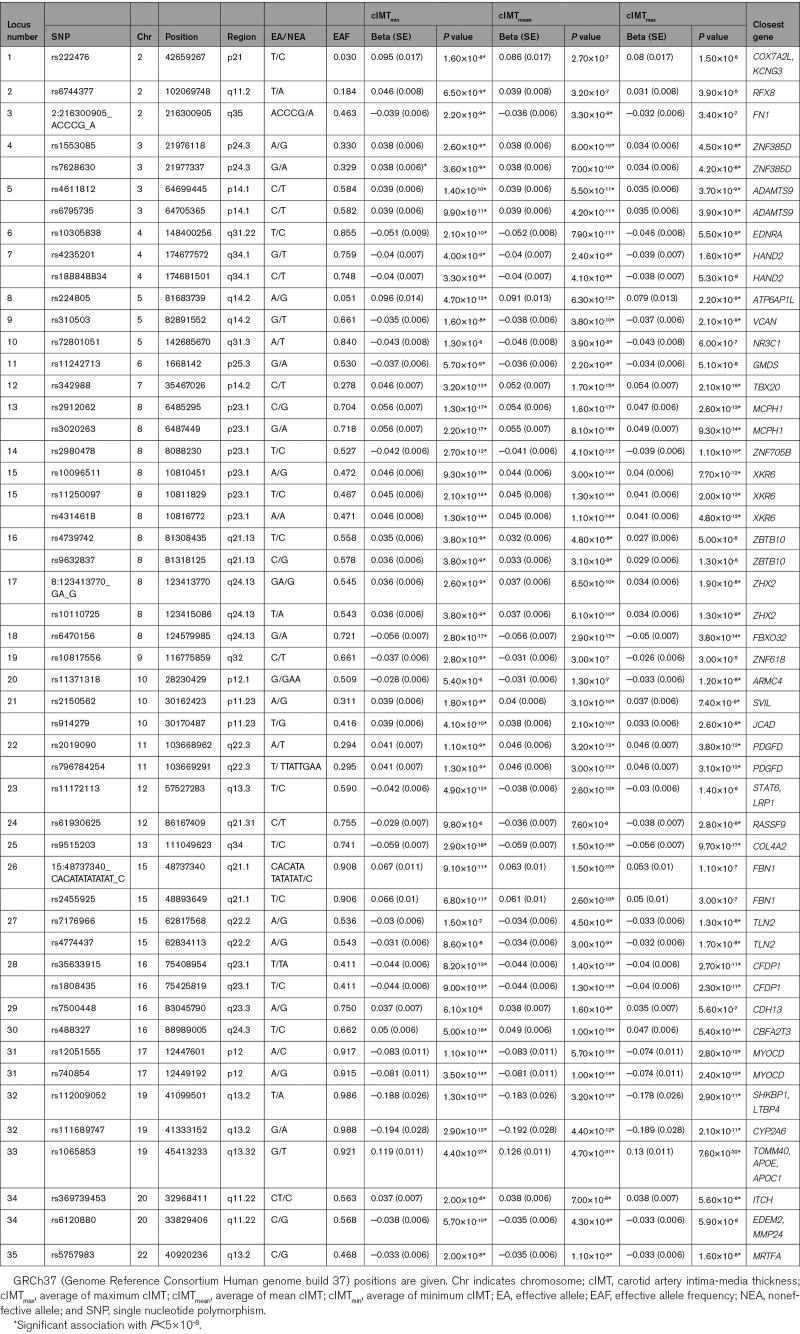
Independent Loci Identified for All cIMT Measurements

**Figure 1.**
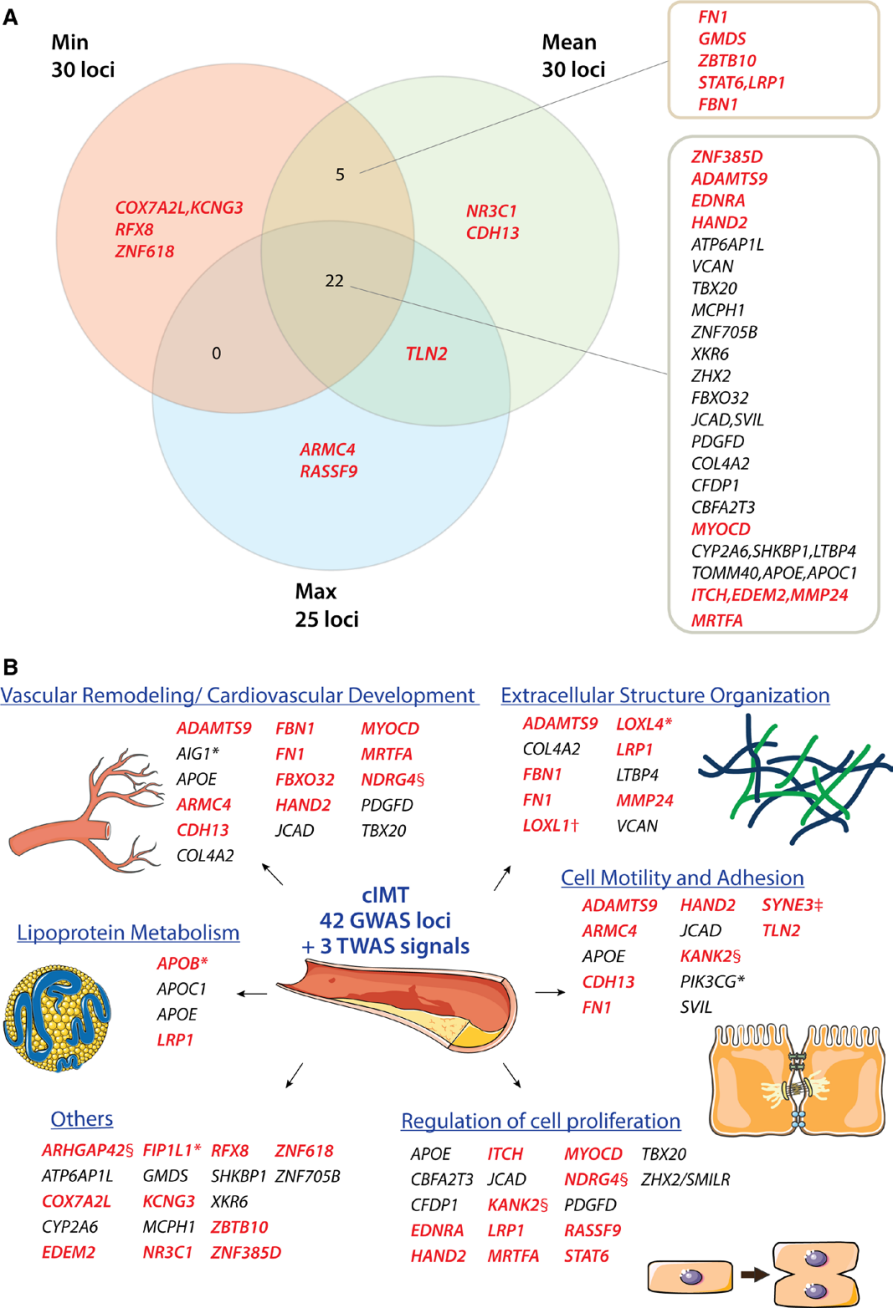
**Overview of independent loci identified for carotid intima-media thickness (cIMT). A**, Overlaps of independent loci identified between genome-wide association study (GWAS) for three cIMT phenotypes. Each locus is marked with the nearest protein-coding gene to the lead variant. Genes marked in bold red font were the closest genes from the respective lead variants of novel loci. **B**, Candidate genes from main GWAS, meta-analyses, and sex interaction analysis were manually grouped into functional categories relevant to atherosclerosis. Genes marked in bold red font were from novel loci; *genes discovered in the meta-analysis of UKB (UK Biobank) and CHARGE (Cohorts for Heart and Aging Research in Genomic Epidemiology)/UCLEB (UCL-LSHTM-Edinburgh-Bristol) consortia; †discovered in meta-analysis of three cIMT measurements; ‡sex-specific locus; §genes discovered in transcriptome-wide association study (TWAS) not belonging to any GWAS loci. Parts of the figure were modified from materials provided by Servier Medical art, licensed under a Creative Common Attribution 3.0 Generic License (http://servier.com/Powerpoint-image-bank). max indicates average of maximum cIMT; mean, average of mean cIMT; and min, average of minimum cIMT.

Comparing the 3 GWAS that were performed in the current study, there was a high degree of overlap in the loci identified at *P*<5×10^-8^ (Figure 1). Notably, cIMT_min_ and cIMT_mean_ shared 27 out of 30 loci, whereas 22 out of 25 loci identified for cIMT_max_ were also detected for cIMT_mean_. A total of 22 loci overlapped between the 3 GWAS, 7 of which are novel: 3p24.3 (*ZNF385D*), 3p14.1 (*ADAMTS9*), 4q31.22 (*EDNRA*), 4q34.1 (*HAND2*), 17p12 (*MYOCD*), 22q11.22 (*ITCH, EDEM2, MMP24*), and 22q13.2 (*MRTFA*). Association results by each cIMT measurement are provided in Table S5 through S7 in the Supplemental Material. Regional association plots for each locus are provided in Figure S4 in the Supplemental Material.

To refine the signals identified at these loci, we performed statistical fine-mapping using FINEMAP and then looked up the coding variants in high LD (R^2^>0.8) with the fine-mapped variants in the dbNSFP database. The closest gene(s) of the fine-mapped variants were the same as the lead variants for the majority of loci except for the locus at 2p21—the fine-mapped variant rs6736913 is close to *EML4* (Table S8 in the Supplemental Material). Additional signals were identified by FINEMAP at 5q31.3 (rs258811, intronic variant of *ARHGAP26*), at 12q13.3 (rs7484541, intronic variant of *R3HDM2*), and at 16q23.1 (rs4888367, intronic variant of *BCAR1*). A total of nine coding variants were found in dbNSFP for 8 loci (Table S8 in the Supplemental Material).

### MTAG Analysis

A total of 6 725 452 variants were analyzed in the meta-analysis of UK Biobank and the CHARGE/UCLEB consortia. The GWAS-equivalent sample sizes estimated by MTAG under the assumptions of LD score regression were 31 887 for UK Biobank cohort and 68 366 for the CHARGE/UCLEB consortia data. Table 2 presents the loci identified in this meta-analysis. Of the 15 loci identified, 10 were also detected in the main individual GWAS within the UK Biobank cohort (Figure 1); loci at 6q24.2 (*AIG1*) and 7q22.3 (*PIK3CG*) were reported in the CHARGE/UCLEB consortia meta-analysis. Three novel loci are 2p24.1 (*APOB*), 4q12 (*FIP1L1*), and 10q24.2 (*LOXL4*), although suggestive evidence for association (*P*<5×10^-^^7^) was already reported in the CHARGE/UCLEB consortia meta-analysis for *APOB* and *LOXL4*. The regional plots of these loci are presented in Figure S5 in the Supplemental Material.

**Table 2.**
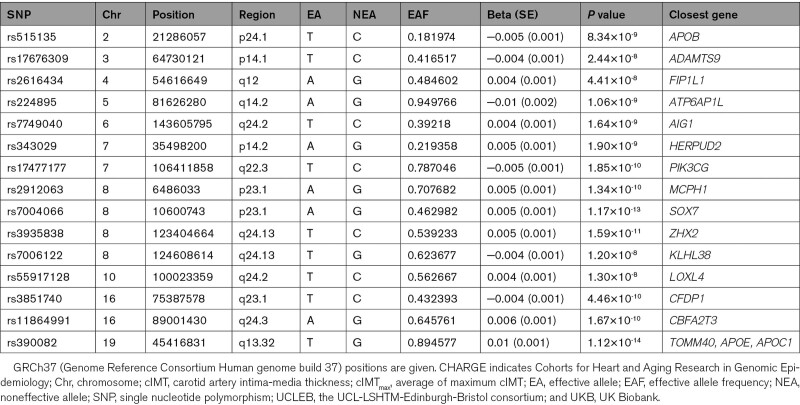
Independent Loci for cIMT_max_ in the Meta-Analysis of UKB and CHARGE/UCLEB Consortia

Meta-analysis of 7 320 435 variants within the UK Biobank across all cIMT measurements revealed 23 loci, 22 of which were the overlapping loci detected in the individual GWAS (Figure 1). The signal at 15q24.1 (*LOXL1*), which did not pass the genome-wide significance threshold in the individual GWAS, was amplified in the meta-analysis (tagged by rs12441130, Beta=−0.033, SE=0.006, *P*_MTAG_=7.87×10^-^^9^). The regional plot of this new locus is presented in Figure S6 in the Supplemental Material. The stop-lost variant rs12441130 was also prioritized by FINEMAP as the most probable causal variant in this region.

### Sex-Specific Loci

Sex-stratified GWAS identified 12, 14, and 8 loci for cIMT_min_, cIMT_mean_, and cIMT_max_, respectively, among female participants; among male participants, there were 9, 7, and 7 loci for cIMT_min_, cIMT_mean_, and cIMT_max_ respectively. All of these loci were reported in the main GWAS except for one male-specific locus at 14q32.13 tagged by rs1209079 (*SYNE3*). These sex-stratified associations were then submitted to the interaction analysis. Out of 42 loci examined, 7 showed significant differences (*P*_sexdiff_ <0.05/42=1.19×10^-3^) between female and male participants (Table S9 and Figure S7 in the Supplemental Material). Two loci 5q14.2 (*VCAN, HAPLN1*) and 8p23.1 (*MCPH1*) have been reported in previous sex-stratified analysis on cIMT_mean._^13^ Among the 5 new loci, 17p12 (*MAP2K4*/*MYOCD*) were significantly associated with cIMT_mean_ and cIMT_max_ in females only, whereas associations at 14q32.13 (*SYNE3*) and the 3 loci at 8p23.1 (*SOX7, GATA4/C8orf49, ZNF705B*) were only significant in males.

### Gene Expression Analyses

**Figure 2.**
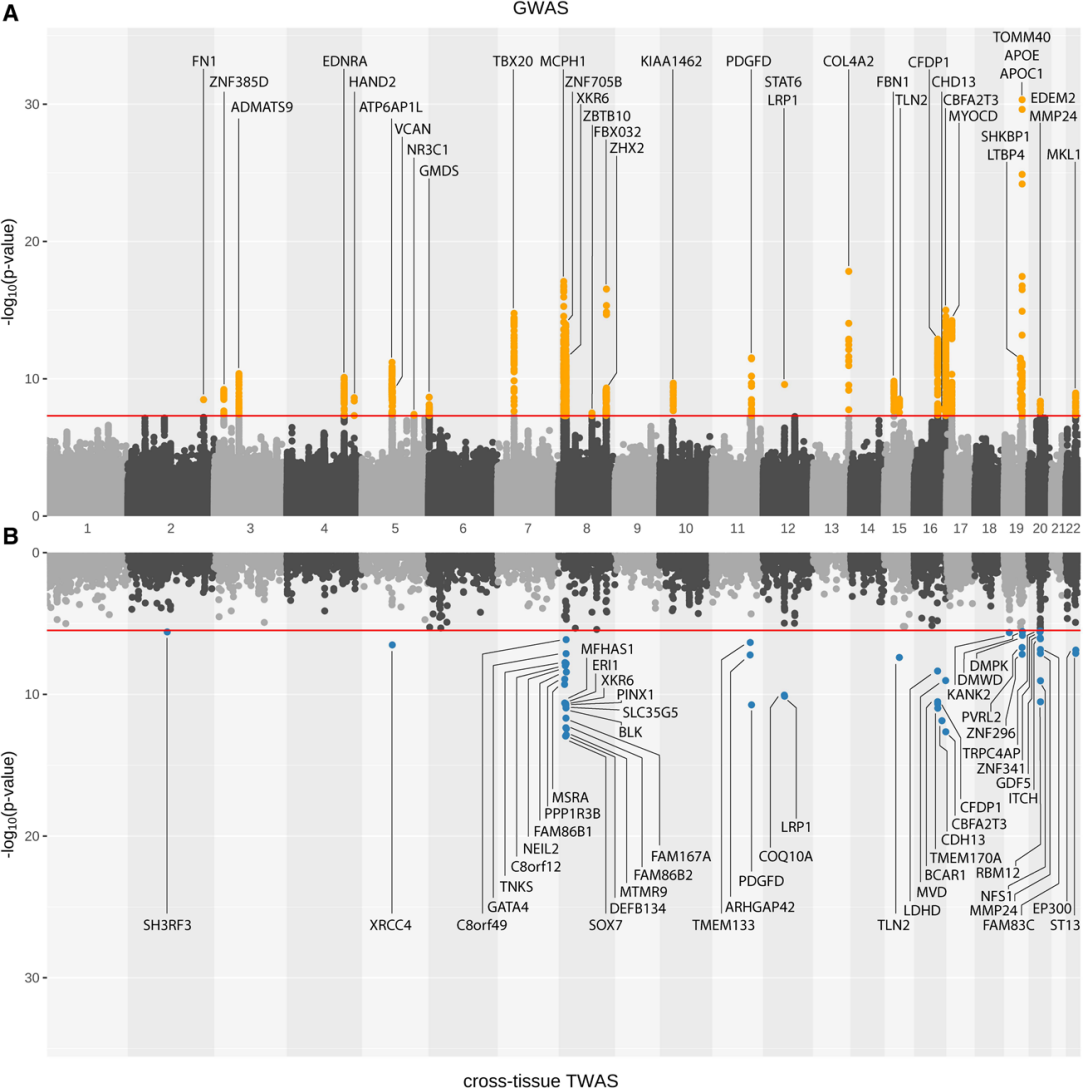
**Miami plot of average of mean carotid intima-media thickness. A** shows genome-wide association study (GWAS) results, whereas **B** shows results from cross-tissue transcriptome-wide association study (TWAS). Orange dots: variants passing genome-wide significance threshold (*P*<5×10^–8^) in GWAS; Blue dots: genes passing significance threshold after Bonferroni correction (*P*<3.3×10^–6^) in TWAS.

Test statistics were available for 15 004 out of 17 290 genes in the cross-tissue analysis on all 44 tissues. From the cross-tissue analysis, we identified 42, 49, and 42 genes with *P*<3.33×10^-6^ (0.05/15 004) for cIMT_min_, cIMT_mean_, and cIMT_max_, respectively (Table S10 in the Supplemental Material). Among these prioritized genes, 35 genes were identified for all three cIMT measurements and were located primarily on chromosome 8, chromosome 16, and 20 nearby the loci identified in the GWAS (Figure 2). Numerous prioritized genes, namely *PINX1*, *BCAR1*, *CDFP1*, and *CBFA2T3*, have been previously associated with cIMT. Although some of the genes prioritized in the TWAS are also the nearest protein-coding gene to the lead variant in the GWAS (*XKR6* at 8p23.1, *PDGFD* at 11q23, *LRP1* at 12q13.3, *TLN2* at 15q22.2, *CDH13* at 16q23.3, *ITCH*, and *MMP24* at 20q11.22), TWAS put forward additional candidate genes such as *XRCC4* at 5q14.2, and *ST13* at 22q13.2 among the GWAS loci. A new association represented by *KANK2* at 19p13.2, which was not found at the GWAS stage, was identified through the cross-tissue TWAS (Figure 2, Figures S8 and S9 in the Supplemental Material).

**Figure 3.**
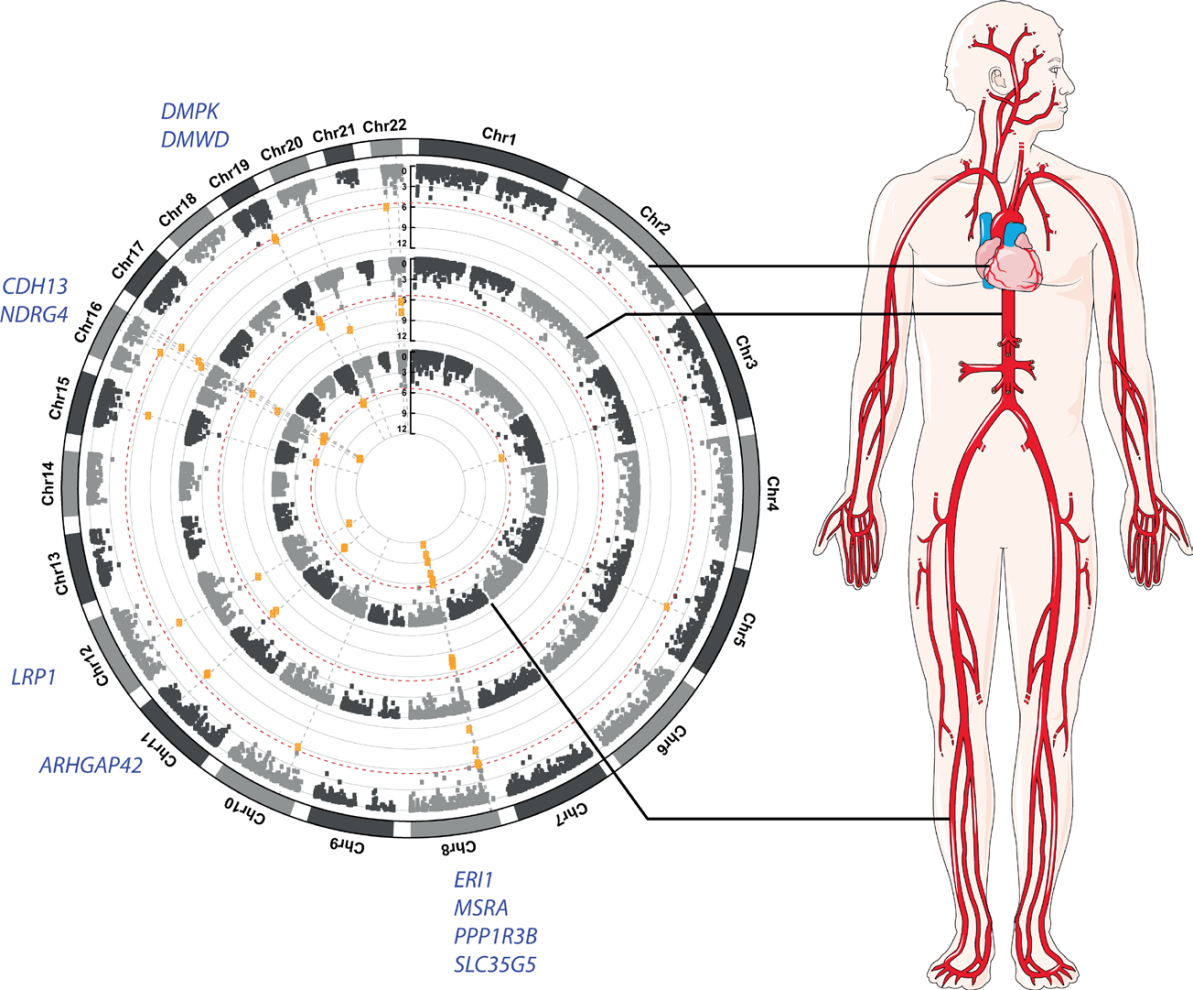
**Circular Manhattan plots of transcriptome-wide association study (TWAS) results in arterial tissues for average of mean carotid intima-media thickness.** Single-tissue TWAS was performed using Genotype-Tissue Expression data on aorta, coronary artery, and tibial artery tissue. Red dotted line: Bonferroni-corrected significance thresholds. Highlighted are the genes that were significant in all arterial tissues. Part of the figure was modified from materials provided by Servier Medical art, licensed under a Creative Common Attribution 3.0 Generic License. Chr indicates chromosome.

Figure 3 depicts the results of the single-tissue tests for cIMT_mean_ with 3 arterial tissues (aorta, coronary, and tibial). A total of 30 genes were prioritized, with 17 from the aorta, 16 from the coronary artery, and 20 from the tibial artery that passed the Bonferroni-corrected *P* value thresholds (4.18×10^-6^, 4.12×10^-6^, and 4.14×10^-6^ respectively; Table S11 in the Supplemental Material). Genes that were consistently prioritized in arterial tissues include *ERI1*, *MSRA*, and *SLC35G5* on chromosome 8; *ARHGAP42* on chromosome 11; *LRP1* on chromosome 12; *CDH13* and *NDRG4* on chromosome 16; and *DMPK* as well as *DMWD* on chromosome 19. Of note, *LRP1* and *CDH13* were also identified in the GWAS; *ARHGAP42* and *NDRG4*, uncovered in arterial-specific TWAS, did not belong to any GWAS loci.

We also performed colocalization analysis with the summary data-based Mendelian randomization (Supplementary Methods). Among the 9 significant genes in summary data-based Mendelian randomization analysis (Table S12 in the Supplemental Material), the majority was also prioritized in either cross-TWAS (*TLN2* and *BCAR1*), arterial TWAS *(ARHGAP42* and *LRP1)*, or both (*CDH13).* Summary data-based Mendelian randomization analysis also identified *SVIL* which was first reported in a previous GWAS.^13^

Finally, we examined the expression of the genes prioritized on a single-cell level in human carotid atherosclerotic plaques samples. UMAP (Uniform Manifold Approximation and Projection) clustering of 6191 cells from 38 patients yielded 18 distinct cell populations including endothelial cells, smooth muscle cells, and various immune cells (Figure S10 in the Supplemental Material). Of the 76 genes with expression data available, 14 genes were highly expressed in smooth muscle cells (*AIG1, EDNRA, FBN1, FBXO32, FDFT1, KANK2, LRP1, LTBP4, PDGFD, PPP1R3B, STAT6, SVIL, TLN2*, and *VCAN*) and 9 were highly expressed in endothelial cells (*ADAMTS9, BCAR1, CDH13, COL4A1, COL4A2, JCAD, NDRG4, SOX7*, and *SVIL*).

### Gene-Based and Gene Set Analysis

Gene-based analyses with MAGMA yielded 44, 54, and 35 genes with *P*<2.63×10^-6^ (0.05/18 991) for cIMT_min_, cIMT_mean_, and cIMT_max_, respectively (Table S13 in the Supplemental Material). We additionally performed gene prioritization using DEPICT (Data-Driven Expression Prioritized Integration for Complex Traits) and found only *GATA4* had a false discovery rate of <5% (Table S14 in the Supplemental Material). An overview of gene prioritization results for GWAS loci identified in the UK Biobank is presented in Table S15 in the Supplemental Material.

Three gene sets passed the Bonferroni-corrected significance threshold in the gene set analyses for cIMT measurements. These included gene sets related to formation and functions of structural protein collagen, platelet-derived growth factor signaling pathway, as well as a gene set within the 20q11 amplicon identified in breast tumor sample. The top 10 gene sets for each cIMT measurements are provided in Table S16 in the Supplemental Material.

### Heritability of cIMT

Using the BOLT-REML algorithm, we estimated the SNP-heritability ($h_{SNP}^{2}$) to be 0.263 (SE=0.013), 0.284 (SE=0.013), and 0.248 (SE=0.013) for cIMT_min_, cIMT_mean_, and cIMT_max_, respectively. Consistent with phenotypic correlation, the cIMT measurements showed very high genetic correlations: cIMT_min_ and cIMT_mean_: *r*_*g*_ =0.989 (SE=0.003); cIMT_min_ and cIMT_max_: *r*_*g*_ =0.975 (SE=0.009); cIMT_mean_ and cIMT_max_: *r*_*g*_ =0.996 (SE=0.002).

A weighted genetic risk score was constructed for each cIMT phenotype, consisting of a summation of the number of effect alleles multiplied by their effect estimate for the respective sentinel SNPs. We found the fractions of cIMT variance explained by the weighted genetic scores of the genome-wide significant SNPs were 2.3%, 2.2%, and 1.8% for cIMT_min_, cIMT_mean_, and cIMT_max_, respectively.

### Shared Genetic Components With CVD and Risk Factors

When testing the genetic correlation with the cardiovascular diseases, the strongest correlations were found for cIMT_max_ and the weakest for cIMT_min_ (Table 3). Among the 3 diseases, the strongest genetic correlations with cIMT were observed for PAD: *r*_*g*_ =0.33 (*P*=0.005), 0.39 (*P*=0.0004), 0.45 (*P*=5.3×10^-5^) for cIMT_min_, cIMT_mean_, and cIMT_max_, respectively. Positive genetic correlation with CAD was observed for all cIMT measurement in UK Biobank with the highest correlation for cIMT_max_: *r*_*g*_=0.21 (*P*=1.4×10^-7^); a similar trend but lower correlations were observed using data from the CARDIoGRAMplusC4D cohort. Positive genetic correlations between ischemic stroke and cIMT measurements were observed using the summary statistics from the MEGASTROKE consortium with the highest correlation for cIMT_max_: *r*_*g*_=0.23 (*P*=1.4×10^-5^), whereas no significant correlation was observed with other stroke subtypes within the MEGASTROKE data nor with stroke within the UK Biobank cohort. Likewise, we found no significant genetic correlations with AAA within the UK Biobank cohort. Among the correlation with CVD risk factors, we found significant positive correlations between all cIMT measurements and SBP in both GERA and UK Biobank cohort with the highest correlation for cIMT_max_: *r*_*g*_=0.30 (*P*=4.0×10^-18^) observed in the UK Biobank. A negative correlation was observed for cIMT_max_ with HDL-C: *r*_*g*_=−0.12 (*P*=7.0×10^-4^).

**Table 3.**
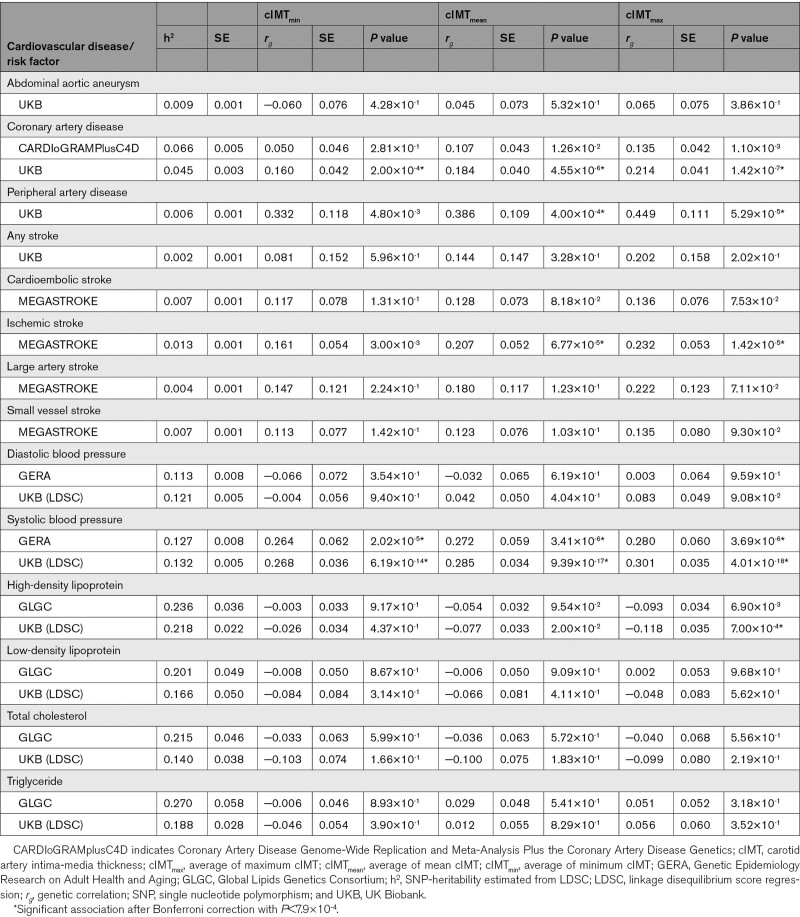
Genetic Correlation Analyses With Cardiovascular Diseases and Risk Factors

The shared genetic background was evident from overlaps of loci between the current GWAS and previous GWAS on CVD/risk factors as listed in GWAS Catalog.^34^ Studies reported association of *APOB* with CAD,^35^ pulse pressure,^36^ LDL-C,^37–43^ and total cholesterol.^42,44^ *ADAMTS9* has been implicated in previous GWAS of CAD,^35^ DBP, SBP^32,45^ as well as total cholesterol level.^43^ Association with CAD,^35,46,47^ ischemic large artery stroke,^31,46^ intracranial aneurysm,^48^ PAD,^49^ pulse pressure,^32,50,51^ and SBP^32,52^ were previously reported for endothelin receptor gene *EDNRA*. *AIG1* was found to be associated with pulse pressure.^51^ *TBX20* was found associated with DBP in a large meta-analysis on blood pressure traits.^36,50^ *PIK3CG* was found to be associated with CAD,^35^ DBP,^51^ SBP,^32,45,52,53^ and pulse pressure.^32,51,54–57^ The region at 8p23.1, marked by 2 loci in the current study (*ZNF705* and *XKR6*), was associated with CVD risk factors namely, pressure traits including DBP,^32,45^ SBP^32,45,51–53^ and pulse pressure,^32,51^ and lipid traits, including LDL-C^43^ and triglycerides.^58,59^ Numerous studies also reported this region may be associated with the interplay between CVD risk factors.^60,61^ *ARMC4* was found to be associated with SBP.^36,52^ Several studies reported *PDGFD* to be associated with CAD.^35,53,62,63^ *LRP1* has been reported to be associated with AAA^64^ as well as cervical artery dissection^65^ and CAD.^35^ In addition to the association with CAD,^35,63^ the *COL4A1*/*COL4A2* region has also been found to be associated with ischemic stroke (small vessel).^31^ The *FBN1* gene, whose detrimental mutation leads to genetic connective disorder Marfan syndrome, perhaps expectedly, was found to associate with pressure traits as well as aneurysms and dissections.^66,67^ The signal identified at *TLN2* was also a locus for DBP and pulse pressure.^51^ Association with CAD^35,53^ and blood pressure traits^32,50–53^ were reported for the locus at 16q23.1 (*CFDP1*) as well as 16q23.3 (*CDH13*). The locus 19q13.32 (*TOMM40*/*APOE*/*APOC1*) is a widely replicated locus for lipid traits including apolipoproteins levels,^68^ LDL-C,^33,37–40,42,43,69–77^ HDL-C,^33,42–44,56,72,74,76,78^ total cholesterol,^33,42,43,56,72,74–76,78^ triglycerides^42^ as well as for CAD.^35,47,63^ Table S17 shows the 14 cIMT variants which were also significantly associated with CAD from the meta-analysis of UK Biobank and CARDIoGRAMplusC4D.^35^

## Discussion

We investigated the genetic architecture of cIMT, a widely used marker of preclinical atherosclerosis, by performing GWAS in over 45 000 individuals of the UK Biobank. We performed secondary analyses including sex-stratified analysis and the meta-analysis of the three cIMT measurements which revealed one additional novel locus, respectively. We also conducted a meta-analysis combining the UK Biobank data and the CHARGE/UCLEB consortia data (GWAS-equivalent sample size=100 253) which resulted in 3 additional new loci. In total, we identified 25 novel loci associated with cIMT. We followed up with our findings by a series of bioinformatic analyses including statistical fine-mapping, expression analyses, gene-based analyses to refine the signals and prioritize candidate genes in the respective loci. Gene set analysis revealed enrichment of candidate genes in biological pathways related to structural components and formation of extracellular matrix, regulation of angiogenesis as well as lipid metabolism. Our results provide novel leads to dissect the complex process of atherosclerosis and targets of therapy.

### Comparison With Previous Studies

The results confirm previous reports on genetics of cIMT traits. We replicated all SNPs associated with cIMT_mean_, and all except one (rs11025608) for cIMT_max_ in a previous GWAS that used a proportion of the current UK Biobank samples.^13^ We also confirmed 7 of 11 loci that were identified in the CHARGE/UCLEB consortia meta-analysis, including 31 studies with various study designs.^12^ These include variants at *ATP6AP1L, MCPH1, SGK223* (moderate LD of 0.267 between the 2 lead variants rs11785239 and rs2980478 near *ZNF705B* identified in the current analysis), *ZHX2*, *PINX1, CBFA2T3*, and *APOE.* In addition to replicating these 15 previously reported loci, we report an additional 25 novel GWAS loci for cIMT, thereby doubling the number of loci associated with cIMT. It is of note that *ADAMTS9* was previously found in colocalization analysis and *EDNRA* was associated with carotid plaque in the mentioned meta-analysis.^12^ Moreover, our TWAS analyses suggested a link between cIMT and *KANK2* from cross-tissue analysis, as well as *ARHGAP42* and *NDRG4* from arterial-specific analysis, which were signals that had not been identified by GWAS.

### Biological Insights of Selected Candidate Genes

Based on the multiple findings of the current study, either by proximity to the lead variants, identification of nonsynonymous coding variants in high LD or various gene-based prioritization analyses (Table S15 in the Supplemental Material) as well as existing literature, we discuss the candidate genes more relevant to vascular biology below. Various candidate genes identified in the present GWAS have known effects on atherosclerosis progression, for instance through their relation with vascular remodeling, extracellular matrix organization, and abnormal lipid metabolism (Figure 1). Other than the Nikolsky breast cancer 20q11 amplicon, whose region coincides with the locus at 20q11.22 in our GWAS, gene sets prioritized in the enrichment analysis with MAGMA revolve mainly around remodeling of connective tissues. Both collagen IV with *COL4A1/COL4A2* (encoding 2 of the 6 alpha chains) and peptidyl lysine oxidases (*LOXL1* and *LOXL4*) are involved in the organization of extracellular matrix (crosslinking of collagen fibrils). Current analysis also identified fellow extracellular matrix components fibrillin-1 (*FBN1*, the gene responsible for the congenital connective disorder Marfan syndrome) and fibronectin-1 (*FN1*). It is of note that both *ADAMTS9* and *MMP24* encode metalloproteinases also involved in maintenance of extracellular matrix. *ADAMTS9* belongs to the same subgroup as *ADAMTS1* as this group cleaves the proteoglycan versican, a structural component of the extracellular matrix encoded by *VCAN.*^79^ Antiangiogenic activity of *ADAMTS9* has been reported with a gene knockdown study,^80^ whereas in human studies, *ADAMTS9* has been associated with an interaction effect with smoking in coronary artery calcification^81^ and higher serum *ADAMTS9* levels have been found in CAD patients.^82^ *MMP24* encodes a metalloproteinase which activates gelatinase A, another matrix metalloproteinase expressed in vascular cells and encoded by *MMP2.*^83^ Extensive studies on both animal and human arteries suggest an important role of *MMP2* in vascular remodeling by degrading collagen IV at basement membrane which facilitates vascular smooth muscle cell (VSMC) migration.^84,85^ It is worth noting that increased MMP activities were observed which contributes to aortic aneurysm formation in human^86^ and mouse^87,88^ with defective *FBN1* via interaction with the large latent complex (of which fibrillin binding protein LTBP4 [latent transforming growth factor beta-binding protein 4] is a member). This further suggests involvement of metalloproteinases in arteriopathy. Altered metalloproteinases activities at atherosclerotic lesions may be attributed to endothelial to mesenchymal transition promoted by *FBXO32.*^89,90^ In the current study, we also identified another endothelial to mesenchymal transition associated gene: heart and neural crest derivatives-expressed 2 (*HAND2*) encodes a transcription factor involved in embryonic cardiogenesis and postnatal ventricular structural remodeling^91^ but has also been implicated in the regulation of angiogenesis.^92^ Loss-of-function mutations of *HAND2* were previously associated with familial dilated cardiomyopathy^93^ and congenital heart defects.^94^

In addition to degradation of extracellular matrix, atherosclerosis also involves accumulation of VSMC at the lesion site. The PDGF signaling pathway, identified in the gene set analysis, is important for migration and proliferation of VSMC. Notably, *STAT6*, a gene close to the new locus identified on chromosome 12, encodes for one of the members of the PDGF pathway, whereas *LRP1* at the same locus encodes for a signaling receptor, a known modulator of the PDGF signaling pathway and has been found to be an important protein in maintaining vascular wall integrity.^95,96^ Interestingly, a recent study reported *SMILR*, a long noncoding RNA located in the consistently replicated locus at 8q24.14 (*ZHX2*), as important mediator for VSMC proliferation in atherosclerosis upon activation by the PDGF signaling pathway.^97^ Of note, the PDGF receptor alpha gene (*PGDFRA*) is located in the novel locus detected in the meta-analysis of UK Biobank and CHARGE/UCLEB consortia in the current study.

Other novel genes identified in the current study may also be involved in regulation of VSMC proliferation, including *CDH13*,^98,99^ *TLN2*,^100^ *KANK2*, *NDRG4*, *MYOCD, MRTFA*, and *EDNRA*. KN motif and ankyrin repeat domains 2 (*KANK2*), which was found through the cross-tissue TWAS, encodes a protein with roles in cytoskeletal formation. *KANK2* was found to be more expressed in smooth muscle cells and endothelial cells in atherosclerotic plaques and has been reported to interact with talins.^101,102^ It has been proposed as a candidate causal gene for CAD in a recent study.^103^ Animal studies reported the role of *NDRG4* in cell proliferations in brain^104^ and the heart^105^ as well as in VSMCs via PDGF signaling pathway.^106^ Myocardin (*MYOCD*) encodes a nuclear protein that is expressed in cardiomyocytes and smooth muscle cells–containing tissues and has been shown to regulate inflammatory and lipid metabolism, both important to the pathogenesis of atherosclerosis, in VSMCs.^107,108^ Myocardin-related transcription factor A (*MRTFA*) encodes a protein that interacts with myocardin. A previous study in a Japanese population reported an association between *MRTFA* and a higher risk of CAD as well as with the severity of CAD.^109^ In mouse models, *MRTFA* was reported to be associated with the accumulation of proatherogenic macrophages in atherosclerotic plaques.^110^ Endothelin receptor type A (*EDNRA*), expressed in smooth muscle cells, encodes a receptor for endothelin-1, the most potent endogenous vasoconstrictive peptide. Numerous studies have found binding of endothelin-1 to the receptor promotes cell proliferation contributing to atherosclerosis.^111^ eQTL (expression quantitative trait loci) analyses in the current study prioritized other vascular molecules related to vascular tone regulation, Rho GTPase-activating protein 42 (*ARHGAP42*) and *ARHGAP26* (closest gene of fine-mapped variant at chromosome 5), although its link to atherosclerosis is unclear.^112^

The loci at 8p23.1 have been consistently reported to be associated with cIMT, although the exact genes involved and the associated molecular mechanisms remain unclear. Among genes in this region, the X Kell blood group complex subunit-related family member 6 (*XKR6*) gene was not only found in all three GWAS but also in TWAS and MAGMA analyses for all cIMT measurements. Although the exact function of *XKR6* is currently unknown, a recent study reported associations between *XKR6*, CAD, and ischemic stroke.^113^ Interestingly, this region was also found significant in the sex interaction analysis with the male-specific effects. Among the highlighted genes are *GATA4* and *SOX7*, which were also prioritized in MAGMA, TWAS, and DEPICT. Studies in individuals with rare 8p23.1 duplication/deletion syndrome suggested possible involvement of both genes in cardiovascular malformations among the patients.^114–116^

### Strengths and Limitations

A major strength of this study is the use of a large sample size from a single cohort. This study is the largest single-sample study of cIMT to date. Previous studies have meta-analyzed various cohorts which were heterogenic in population and phenotyping.^12^ By using a single cohort with a standardized method for cIMT measurements and rigid quality control, our analyses likely suffer less from heterogeneity. There is no consensus on the optimal cIMT assessment in the literature with reporting of mean or maximum cIMT values most common, although it has been suggested measurement of maximum values may be more susceptible to sampling error.^117,118^ In the current study, we analyzed all cIMT values available in the UK Biobank, namely cIMT_min_, cIMT_mean_, and cIMT_max_, which may facilitate comparison with other literature. Second, we performed not only GWAS but also sought to further characterize and prioritize genes from loci identified in GWAS by performing follow-up analyses leveraging external datasets. This allowed us to gather additional evidence, also in specific arterial tissues, for multiple genetic loci and provide additional insights into other novel loci that were not identified by GWAS but maybe interesting candidate genes for cIMT. The combination of evidence from these multiple data sources warrants further study of these genes in future (experimental) studies. The current study also has limitations. First, our analyses were performed in mostly Europeans, which may limit the generalizability of our results to other ethnic groups. Second, TWAS analyses have intrinsic limitations due to their methodology. The method is based on the prediction of the genetically-regulated component of gene expression and does not consider other factors that influence the expression (environmental and technical components). Additionally, false-positive signals may have arisen due to co-regulation of gene expression and TWAS may suffer in power to detect true risk genes in tissues not related to the trait.^119^ We, therefore, presented TWAS results on 3 arterial tissues (aorta, coronary, and tibial) which are mechanistically most relevant to cIMT. The prediction of gene expression in TWAS is based on the external reference panel which has a limited size of available samples for several tissues. The models for coronary artery, aorta, and tibial artery are based on 200 to 600 samples, which limits the definitive power of expression prediction. To tackle this issue, we additionally applied a TWAS approach that utilizes a joint imputation of gene expression in multiple tissues.^24^

### Conclusions

In conclusion, the present study characterized the genetic architecture underlying cIMT and identified 25 novel loci. We found sex differences in 7 cIMT loci. We prioritized genes that are potentially more relevant for cIMT through extensive-expression analyses using external datasets (Genotype-Tissue Expression and AtheroExpress). Gene set analyses provided additional evidence for candidate genes and highlighted biological pathways. Finally, we found shared genetic components between cIMT and various cardiovascular diseases and risk factors, namely CAD, PAD, SBP, and HDL-C. These findings warrant further investigation for the role of these genetic loci in the development and treatment options of atherosclerosis.

## Article Information

### Acknowledgments

We thank the CARDIoGRAMplusC4D (Coronary Artery Disease Genome-Wide Replication and Meta-Analysis Plus the Coronary Artery Disease Genetics), the GERA study (Genetic Epidemiology Research on Adult Health and Aging), GLGC (Global Lipids Genetics Consortium) and MEGASTROKE investigators for making their data publicly available. The MEGASTROKE project received funding from sources specified at http://www.megastroke.org/acknowledgments.html. We would like to thank the Centre for Information Technology of the University of Groningen for their support and for providing access to the Peregrine high-performance computing cluster. In addition, we thank Ruben N. Eppinga, MD, PhD, Tom Hendriks, MD, PhD, M. Yldau van der Ende, MD, PhD, Hilde E. Groot, MD, Jan Walter Benjamins, BEng, and Yanick Hagemeijer, MSc, University of Groningen, University Medical Center Groningen, Department of Cardiology, for their contributions to the extraction and processing of data in the UK Biobank. None of the mentioned contributors received compensation, except for their employment at the University Medical Center Groningen.

### Sources of Funding

None.

### Disclosures

N. Verweij is a paid consultant for Regeneron Pharmaceuticals. The other authors report no conflicts.

### Supplemental Materials

Supplementary Methods

Tables S1–S17

Figures S1–S10

Major Resources Table

Supplementary Note

Reference 120–124

## Supplementary Material


