# Supplementary material for: Twenty-Five Novel Loci for Carotid Intima-Media Thickness: A Genome-Wide Association Study in >45 000 Individuals and Meta-Analysis of >100 000 Individuals
Source: Arterioscler Thromb Vasc Biol. 2021 Dec 2;42(4):484–501. doi: 10.1161/ATVBAHA.121.317007 (PMC8939707; doi:10.1161/ATVBAHA.121.317007)
Supplement: Supplementary file 1 [file atv-42-484-s001.pdf]

SUPPLEMENTAL MATERIAL

25 novel loci for carotid intima-media thickness: a genome-wide association study in >45,000 individuals and meta-analysis of >100,000 individuals

Ming Wai Yeung, MSc[1,2]; Siqi Wang, MSc[1,3]; Yordi J. van de Vegte, BSc[1]; Oleg Borisov,MD,PhD[4]; Jessica van Setten,PhD[2]; Harold Snieder,PhD[3]; Niek Verweij, PhD[1]; M. Abdullah Said, MD, PhD[1]; Pim van der Harst, MD, PhD[1,2]

[1] University of Groningen, University Medical Center Groningen, Department of Cardiology, 9700 RB Groningen, the Netherlands

[2] Department of Cardiology, Division of Heart & Lungs, University Medical Center Utrecht, University of Utrecht, Utrecht, the Netherlands

[3] Department of Epidemiology, University of Groningen, University Medical Center Groningen, 9700 RB, Groningen, The Netherlands.

[4]Institute for Genomic Statistics and Bioinformatics, University Hospital Bonn, Bonn, Germany

Contents:

Supplementary Methods

Tables S1-S17

Figures S1-S10

Major Resources Table

Supplementary Note

Reference 121-125

Supplementary Methods

CVD risk factors measurements in UK Biobank

Cholesterol and triglyceride were measured using enzymatic assays; High density lipoprotein cholesterol (HDL-C) was measured using an enzyme Immuno-inhibition method while low density lipoprotein cholesterol (LDL-C) was measured using an enzymatic selective protection method[121]. Cholesterol/LDL-C levels of individuals with cholesterol lowering medication at time of inclusion were adjusted by dividing by 0.8/0.7 respectively.

Systolic and diastolic blood pressure were measured twice at a sitting position during participants' visits to assessment centers. The measurements were done first with an automated blood pressure device (Omron 705 IT electronic blood pressure monitor; OMRON Healthcare Europe B.V. Kruisweg 577 2132 NA Hoofddorp) (Field ID 4079, 4080)[122]; In case of unsuccessful automatic measurements, the blood pressures were manually measured by trained nurses (Field ID 93, 94). The average of multiple available measurements was taken as the blood pressure for each participant.

eQTL colocalization analysis with summary data based Mendelian randomization (SMR)

To prioritize genes identified in GWAS, we performed SMR analysis (version 0.710) to test for association between the expression level of each gene and cIMT by using the top-associated expression quantitative trait locus (eQTL) as an instrumental variable[123]. We conducted SMR analysis using the GTEx dataset (v7) but only on tissues relevant to cIMT, namely artery aorta, artery coronary and artery tibial[25]. Genes with p-value (P_{SMR}) passing Bonferroni-corrected thresholds, which was corrected based on the number of genes tested, were submitted to HEterogeneity In Dependent Instruments (HEIDI) test. Genes significant at SMR analysis and not rejected by HEIDI test (P_{HEIDI} >tissue specific Bonferroni-corrected thresholds).

Gene expression analysis in single-cell RNA-sequencing data in human carotid atherosclerotic plaques

The expression levels genes prioritized in GWAS along with other bioinformatic analyses were explored in a RNA-sequencing dataset of carotid artery plaque tissues from 38 patients on a single-cell level with a total of 6191 cells[102]. Analysis was done using PlaqView[124].

Gene prioritization with DEPICT

we used the bioinformatic tool DEPICT (v1.1beta, obtained from https://data.broadinstitute.org/mpg/depict/) to identify candidate causal genes whilst taking gene-gene similarities and LD structures between loci into consideration[125]. Variants at a P-value threshold of $P<1 \times 10^{-8}$ were submitted to this analysis.

Table S1. Clinical characteristics of the study population at imaging visit

Characteristics	All	Men	Women
N	45185	21827	23358
Age (years), mean (SD)	64.68 (7.72)	65.41 (7.81)	64.01 (7.56)
Sex (men)	21827 (48.3%)		
BMI (kg/m2), mean (SD)	26.52 (4.38)	26.97 (3.89)	26.09 (4.76)
LDL-C in mmol/l, median (IQR) *	3.54 (3.00,4.11)	3.54 (2.99, 4.10)	3.54 (3.01, 4.13)
HDL-C in mmol/l, median (IQR) *	1.43 (1.20,1.70)	1.26 (1.09, 1.48)	1.60 (1.37,1.86)
Cholesterol in mmol/l, median (IQR)*	5.68 (4.98, 6.42)	5.56 (4.85, 6.28)	5.79 (5.11, 6.55)
Triglyceride in mmol/l, median (IQR)*	1.40 (0.99, 2.02)	1.63 (1.15, 2.35)	1.22 (0.90, 1.72)
Blood pressure (mm Hg), mean (SD)			
Systolic	134.43 (17.96)	140.18 (16.15)	129.01 (17.88)
Diastolic	79.24 (8.54)	81.7 (8.15)	76.92 (8.24)
$cIMT_{min}$ (mm), mean (SD)	0.58 (0.11)	0.6 (0.12)	0.57 (0.1)
$cIMT_{mean}$ (mm), mean (SD)	0.69 (0.03)	0.71 (0.14)	0.67 (0.11)
$cIMT_{max}$ (mm), mean (SD)	0.8 (0.15)	0.83 (0.16)	0.77 (0.13)
Ethnicity			
White	43775 (96.9%)	21123 (96.8%)	22652 (97.0%)
Asian	612 (1.4%)	356 (1.6%)	256 (1.1%)
Black	291 (0.6%)	138 (0.6%)	153 (0.7%)
Mixed	215 (0.5%)	77 (0.4%)	138 (0.6%)
Other/Unknown	292 (0.6%)	133 (0.6%)	159 (0.7%)
Current smoker	3165 (7.0%)	1814 (8.3%)	1348 (5.8%)
Hypertension	15384 (34.0%)	8616 (39.5%)	6157 (26.4%)
Hyperlipidemia	12763 (28.2%)	8082 (37.0%)	4681 (20.0%)
Abdominal aortic aneurysm	146 (0.3%)	127 (0.6%)	19 (0.1%)
Cerebral infarction	367 (0.8%)	253 (1.2%)	114 (0.5%)
Coronary artery disease	2718 (6.0%)	2073 (9.5%)	645 (2.8%)
Peripheral artery disease	490 (1.1%)	281 (1.3%)	209 (0.9%)

* Measurements collected at the baseline visit.

Table S2. Variable definitions used in the UK Biobank

Variable	ICD-9	ICD-10	OPCS-4	Self-reported fields
Abdominal aortic aneurysm	4414,4413,44102	I714, I713	L183, L184, L185, L186, L193, L194, L195, L196, L271, L272, L275, L281, L282, L464	20002(1492, 1591, 1592), 20004(1104)
Coronary artery disease	414, 410, 412	I24, I25, Z955, I21, I22, I23, I252, Z951	K40, K41, K42, K43, K44, K45, K46, K49, K50, K75	20002(1075), 20004(1070, 1095, 1523)
Ischemic stroke	434	I63, I693, G951, H341, H342		20002(1583)
Hypertension	402, 403, 404, 405, 401	I11, I12, I13, I15, O10, I10		20002(1065, 1072)
Hyperlipidemia	272	E78		20002(1473)
Peripheral artery disease in extremities (include aneurysms)	4439	I739	L37, L381, L383, L384, L391, L392, L395, L48, L49, L50, L51, L52, L53, L541, L542, L544, L56, L57, L58, L59, L60, L62, L631, L632, L635, L638, L639, L653	20002(1087, 1067), 20004(1102, 1103, 1108)
Cholesterol lowering medication				6177(1), 6153(1),20003

Variable definitions constructed using ICD-9, ICD-10 and OPCS-4 codes as well as self-reported data fields with disease- or procedure-specific codes between brackets are shown. Abbreviations: ICD, International Classification of Diseases; OPCS, Office of Population, Censuses and Surveys: Classification of interventions and Procedure

Table S3. Replication of loci from UK Biobank data in CHARGE/UCLEB consortia meta-analysis

			$cIMT_{min}$		$cIMT_{mean}$		$cIMT_{max}$		Franceschini *et al*						
**#**	**SNP**	**EA**	**Beta (SE)**	***P***	**Beta (SE)**	***P***	**Beta (SE)**	***P***	**Proxy SNP**	**EA**	**I^2**	**Beta (SE)**	***P***	**N**	**consistent**
1*	rs222476	T	0.095 (0.017)	1.60E-08	0.086 (0.017)	2.70E-07	0.08 (0.017)	1.50E-06		T	0	-0.001 (0.0027)	8.16E-01	64611	n
2*	rs6744377	T	0.046 (0.008)	6.50E-09	0.039 (0.008)	3.20E-07	0.031 (0.008)	3.90E-05	rs144206469	D	17.7	0.003 (0.001)	**3.41E-03**	57141	y
3*	2:216300905_ACCCG_A	ACCCG	-0.039 (0.006)	2.20E-09	-0.036 (0.006)	3.30E-09	-0.032 (0.006)	3.40E-07	rs1250237	T	0	0.0015 (0.0007)	**3.69E-02**	64953	y
4*	rs1553085	A	0.038 (0.006)	2.60E-09	0.038 (0.006)	6.00E-10	0.034 (0.006)	4.50E-08		A	14.7	0.001 (0.0008)	7.74E-02	58694	y
5*	rs6795735	C	0.039 (0.006)	9.90E-11	0.039 (0.006)	4.20E-11	0.035 (0.006)	3.90E-09		T	24.1	-0.003 (0.0007)	**4.93E-06**	58799	y
6*	rs10305838	T	-0.051 (0.009)	2.10E-10	-0.052 (0.008)	7.90E-11	-0.046 (0.008)	5.50E-09		T	20.0	-0.004 (0.001)	**7.85E-05**	68002	y
7*	rs4235201	G	-0.04 (0.007)	4.00E-09	-0.04 (0.007)	2.40E-09	-0.039 (0.007)	1.60E-08		T	0.0	0.003 (0.0009)	**3.73E-03**	57794	y
8	rs224805	A	0.096 (0.014)	4.70E-12	0.091 (0.013)	6.30E-12	0.079 (0.013)	2.20E-09		T	1.3	0.009 (0.0016)	**8.07E-08**	68010	y
9	rs310503	G	-0.035 (0.006)	1.60E-08	-0.038 (0.006)	3.80E-10	-0.037 (0.006)	2.10E-09		T	14.4	0.003 (0.0008)	**7.27E-06**	67971	y
10*	rs72801051	A	-0.043 (0.008)	1.30E-06	-0.046 (0.008)	3.90E-08	-0.043 (0.008)	6.00E-07		A	0.0	-0.001 (0.001)	4.44E-01	67974	y
11*	rs11242713	G	-0.037 (0.006)	5.70E-09	-0.036 (0.006)	2.20E-09	-0.034 (0.006)	5.10E-08		A	0.0	0.002 (0.0007)	**6.62E-03**	67107	y
12	rs342988	C	0.046 (0.007)	3.20E-12	0.052 (0.007)	1.70E-15	0.054 (0.007)	2.10E-16		T	7.4	-0.003 (0.0008)	**1.53E-04**	64995	y
13	rs3020263	G	0.056 (0.007)	2.20E-17	0.055 (0.007)	8.10E-18	0.049 (0.007)	9.30E-14		A	4.3	-0.004 (0.0008)	**2.78E-08**	68003	y
14	rs2980478	T	-0.042 (0.006)	2.70E-12	-0.041 (0.006)	4.10E-12	-0.039 (0.006)	1.10E-10	rs2945319	T	0.0	-0.0018 (0.0009)	5.66E-02	35439	y
15	rs11250097	T	0.045 (0.006)	2.10E-14	0.045 (0.006)	1.30E-14	0.041 (0.006)	2.00E-12		T	33.3	0.004 (0.0007)	**8.36E-07**	67107	y
16*	rs4739742	T	0.035 (0.006)	3.80E-09	0.032 (0.006)	4.80E-08	0.027 (0.006)	5.00E-06		T	9.0	0.002 (0.0008)	**4.82E-03**	57795	y
17	rs10110725	T	0.036 (0.006)	3.80E-09	0.037 (0.006)	6.10E-10	0.034 (0.006)	1.30E-08		A	36.1	-0.004 (0.0007)	**2.35E-08**	67996	y
18	rs6470156	G	-0.056 (0.007)	2.80E-17	-0.056 (0.007)	2.90E-17	-0.05 (0.007)	3.80E-14		A	0.0	0.003 (0.0008)	**4.65E-04**	67952	y

#	SNP	EA	cIMT$_{min}$ Beta (SE)	cIMT$_{min}$ *P*	cIMT$_{mean}$ Beta (SE)	cIMT$_{mean}$ *P*	cIMT$_{max}$ Beta (SE)	cIMT$_{max}$ *P*	Franceschini *et al* Proxy SNP	EA	I^2	Beta (SE)	*P*	N	consistent
19*	rs10817556	C	-0.037 (0.006)	2.80E-09	-0.031 (0.006)	3.00E-07	-0.026 (0.006)	3.00E-05		T	0.0	0.002 (0.0007)	**4.26E-03**	64623	y
20*	rs11371318	G	-0.028 (0.006)	5.40E-06	-0.031 (0.006)	1.30E-07	-0.033 (0.006)	1.20E-08	rs7907935	T	20.8	-0.0011 (0.0007)	1.17E-01	58645	y
21	rs914279	T	0.039 (0.006)	4.10E-10	0.038 (0.006)	2.10E-10	0.033 (0.006)	2.60E-08		A	5.1	0.003 (0.0008)	**4.44E-05**	51484	y
22	rs2019090	A	0.041 (0.007)	1.10E-09	0.046 (0.006)	3.20E-12	0.046 (0.007)	3.80E-12		A	25.3	0.002 (0.0008)	**4.55E-03**	68937	y
23*	rs11172113	T	-0.042 (0.006)	4.90E-12	-0.038 (0.006)	2.60E-10	-0.03 (0.006)	1.40E-06		T	28.5	-0.003 (0.0008)	**1.80E-04**	60726	y
24*	rs61930625	C	-0.029 (0.007)	9.80E-06	-0.036 (0.007)	7.60E-08	-0.038 (0.007)	2.80E-08		T	0.0	0.001 (0.0008)	1.60E-01	62082	y
25	rs9515203	T	-0.059 (0.007)	2.90E-18	-0.059 (0.007)	1.50E-18	-0.056 (0.007)	9.70E-17		T	32.1	-0.004 (0.0009)	**3.12E-06**	61467	y
26*	rs2455925	T	0.066 (0.01)	6.80E-11	0.061 (0.01)	2.60E-10	0.05 (0.01)	3.00E-07		A	3.3	0.005 (0.0013)	**3.54E-04**	55625	y
27*	rs4774437	A	-0.031 (0.006)	8.60E-08	-0.034 (0.006)	3.00E-09	-0.032 (0.006)	1.70E-08		A	0.0	-0.003 (0.0007)	**4.44E-05**	65570	y
28	rs1808435	T	-0.044 (0.006)	9.00E-13	-0.044 (0.006)	1.30E-13	-0.04 (0.006)	2.30E-11		A	16.9	-0.004 (0.0007)	**2.74E-07**	67997	y
29*	rs7500448	A	0.037 (0.007)	6.10E-08	0.038 (0.007)	1.60E-08	0.035 (0.007)	5.60E-07		A	42.4	0.004 (0.0009)	**1.71E-05**	62395	y
30	rs488327	T	0.05 (0.006)	5.00E-16	0.049 (0.006)	1.00E-15	0.047 (0.006)	5.40E-14		T	51.7	0.004 (0.0009)	**1.54E-06**	48337	y
31*	rs12051555	A	-0.083 (0.011)	1.10E-14	-0.083 (0.011)	5.70E-15	-0.074 (0.011)	2.80E-12		A	0.0	-0.005 (0.0014)	**7.80E-04**	57795	y
32	rs112009052	T	-0.188 (0.026)	1.30E-12	-0.183 (0.026)	3.20E-12	-0.178 (0.026)	2.90E-11		A	0.0	0.01 (0.0032)	**2.74E-03**	56919	y
33	rs1065853	G	0.119 (0.011)	4.40E-27	0.126 (0.011)	4.70E-31	0.13 (0.011)	7.60E-32	rs7412	T	46.2	-0.0119 (0.0015)	**1.01E-14**	56530	y
34*	rs6120880	C	-0.038 (0.006)	5.70E-10	-0.035 (0.006)	4.30E-09	-0.033 (0.006)	5.90E-08		C	0.0	-0.003 (0.0007)	**3.06E-04**	68071	y
35*	rs5757983	C	-0.033 (0.006)	2.00E-08	-0.035 (0.006)	1.10E-09	-0.033 (0.006)	1.60E-08		C	9.5	-0.004 (0.0007)	**5.29E-07**	62319	y

Chr: Chromosome; EA: Effective allele; I^2: Heterogeneity I^2 statistics in the meta-analysis; NEA: Non-effective allele; EAF: Effective allele frequency; #: Locus number; *: novel loci discovered in the current study. Proxy SNP rs144206469, rs1250237, rs2945319, rs7907935 and rs7412 are in high LD (R^2>0.7) with lead SNP. Marked in bold: associations in CHARGE/UCLEB consortia meta-analysis with *P* < 0.05.

Table S4. Association results of previously reported cIMT loci not replicated in the current UK Biobank sample

							$cIMT_{min}$		$cIMT_{mean}$		$cIMT_{max}$	
**SNP**	**Closest gene**	**Reported literature**	**Chr**	**Position**	**EA**	**EAF**	**Beta (SE)**	***P***	**Beta (SE)**	***P***	**Beta (SE)**	***P***
rs201648240	*LINC01717*	Franceschini *et al.*	1	208953176								
rs6907215	*AIG1*	Franceschini *et al.*	6	143608968	C	0.39	0.024 (0.006)	2.20E-04	0.023 (0.006)	2.80E-04	0.019 (0.006)	4.00E-03
rs13225723	*PIK3CG*	Franceschini *et al.*	7	106416467	G	0.89	-0.046 (0.011)	8.50E-06	-0.041 (0.011)	8.10E-05	-0.034 (0.011)	1.30E-03
rs11196033	*VTI1A*	Franceschini *et al.*	10	114410998	C	0.50	0.002 (0.006)	6.60E-01	0.001 (0.006)	8.70E-01	-0.002 (0.006)	8.00E-01
rs11025608	*PRMT3, SLC6A5*	Strawbridge *et al.*	11	20554790	G	0.45	-0.022 (0.006)	5.30E-04	-0.023 (0.006)	3.00E-04	-0.023 (0.006)	3.00E-04

Four of five loci was reported in a meta-analysis by Franceschini *et al.*[12], one of which was not found in current results; the additional locus not replicated was reported in Strawbridge *et al.*[13] using a subset of current sample. Chr: Chromosome; EA: Effective allele; EAF: Effective allele frequency.

Table S5. Lead variants in loci associated with $cIMT_{min}$

SNP	Chr	Position	EA	NEA	EAF	Beta	SE	*P*	Region	Closest gene
rs222476	2	42659267	T	C	0.03	0.095	0.017	1.60E-08	p21	*COX7A2L,KCNG3*
rs6744377	2	102069748	T	A	0.184	0.046	0.008	6.50E-09	q11.2	*RFX8*
2:216300905_ACCCG_A	2	216300905	ACCCG	A	0.463	-0.039	0.006	2.20E-09	q35	*FN1*
rs1553085	3	21976118	A	G	0.33	0.038	0.006	2.60E-09	p24.3	*ZNF385D*
rs6795735	3	64705365	C	T	0.582	0.039	0.006	9.90E-11	p14.1	*ADAMTS9*
rs10305838	4	148400256	T	C	0.855	-0.051	0.009	2.10E-10	q31.22	*EDNRA*
rs188848834	4	174681501	C	T	0.748	-0.04	0.007	3.30E-09	q34.1	*HAND2*
rs224805	5	81683739	A	G	0.051	0.096	0.014	4.70E-12	q14.2	*ATP6AP1L*
rs310503	5	82891552	G	T	0.661	-0.035	0.006	1.60E-08	q14.2	*VCAN*
rs11242713	6	1668142	G	A	0.53	-0.037	0.006	5.70E-09	p25.3	*GMDS*
rs342988	7	35467026	C	T	0.278	0.046	0.007	3.20E-12	p14.2	*TBX20*
rs2912062	8	6485295	C	G	0.704	0.056	0.007	1.30E-17	p23.1	*MCPH1*
rs2980478	8	8088230	T	C	0.527	-0.042	0.006	2.70E-12	p23.1	*ZNF705B*
rs10096511	8	10810451	A	G	0.472	0.046	0.006	9.30E-15	p23.1	*XKR6*
rs4739742	8	81308435	T	C	0.558	0.035	0.006	3.80E-09	q21.13	*ZBTB10*
8:123413770_GA_G	8	123413770	GA	G	0.545	0.036	0.006	2.60E-09	q24.13	*ZHX2*
rs6470156	8	124579985	G	A	0.721	-0.056	0.007	2.80E-17	q24.13	*FBXO32*
rs10817556	9	116775859	C	T	0.661	-0.037	0.006	2.80E-09	q32	*ZNF618*
rs914279	10	30170487	T	G	0.416	0.039	0.006	4.10E-10	p11.23	*JCAD*
rs2019090	11	103668962	A	T	0.294	0.041	0.007	1.10E-09	q22.3	*PDGFD*
rs11172113	12	57527283	T	C	0.59	-0.042	0.006	4.90E-12	q13.3	*STAT6,LRP1*
rs9515203	13	111049623	T	C	0.741	-0.059	0.007	2.90E-18	q34	*COL4A2*
rs2455925	15	48893649	T	C	0.906	0.066	0.01	6.80E-11	q21.1	*FBN1*
rs35633915	16	75408954	T	TA	0.411	-0.044	0.006	8.20E-13	q23.1	*CFDP1*
rs488327	16	88989005	T	C	0.662	0.05	0.006	5.00E-16	q24.3	*CBFA2T3*
rs12051555	17	12447601	A	C	0.917	-0.083	0.011	1.10E-14	p12	*MYOCD*
rs112009052	19	41099501	T	A	0.986	-0.188	0.026	1.30E-12	q13.2	*SHKBP1,LTBP4*
rs1065853	19	45413233	G	T	0.921	0.119	0.011	4.40E-27	q13.32	*TOMM40,APOE,APOC1*
rs6120880	20	33829406	C	G	0.568	-0.038	0.006	5.70E-10	q11.22	*EDEM2,MMP24*
rs5757983	22	40920236	C	G	0.468	-0.033	0.006	2.00E-08	q13.2	*MRTFA*

Chr: Chromosome; EA: Effective allele; NEA: Non-effective allele; EAF: Effective allele frequency.

Table S6. Lead variants in loci associated with $cIMT_{mean}$

SNP	Chr	Position	EA	NEA	EAF	Beta	SE	*P*	Region	Closest gene
2:216300905_ACCCG_A	2	216300905	ACCCG	A	0.463	-0.036	0.006	3.30E-09	q35	*FN1*
rs1553085	3	21976118	A	G	0.33	0.038	0.006	6.00E-10	p24.3	*ZNF385D*
rs6795735	3	64705365	C	T	0.582	0.039	0.006	4.20E-11	p14.1	*ADAMTS9*
rs10305838	4	148400256	T	C	0.855	-0.052	0.008	7.90E-11	q31.22	*EDNRA*
rs4235201	4	174677572	G	T	0.759	-0.04	0.007	2.40E-09	q34.1	*HAND2*
rs224805	5	81683739	A	G	0.051	0.091	0.013	6.30E-12	q14.2	*ATP6AP1L*
rs310503	5	82891552	G	T	0.661	-0.038	0.006	3.80E-10	q14.2	*VCAN*
rs72801051	5	142685670	A	T	0.84	-0.046	0.008	3.90E-08	q31.3	*NR3C1*
rs11242713	6	1668142	G	A	0.53	-0.036	0.006	2.20E-09	p25.3	*GMDS*
rs342988	7	35467026	C	T	0.278	0.052	0.007	1.70E-15	p14.2	*TBX20*
rs3020263	8	6487449	G	A	0.718	0.055	0.007	8.10E-18	p23.1	*MCPH1*
rs2980478	8	8088230	T	C	0.527	-0.041	0.006	4.10E-12	p23.1	*ZNF705B*
rs4314618	8	10816772	A	G	0.471	0.045	0.006	1.10E-14	p23.1	*XKR6*
rs9632837	8	81318125	C	G	0.578	0.033	0.006	3.10E-08	q21.13	*ZBTB10*
rs10110725	8	123415086	T	A	0.543	0.037	0.006	6.10E-10	q24.13	*ZHX2*
rs6470156	8	124579985	G	A	0.721	-0.056	0.007	2.90E-17	q24.13	*FBXO32*
rs914279	10	30170487	T	G	0.416	0.038	0.006	2.10E-10	p11.23	*JCAD*
rs796784254	11	103669291	T	TTATTGAA	0.295	0.046	0.006	3.00E-12	q22.3	*PDGFD*
rs11172113	12	57527283	T	C	0.59	-0.038	0.006	2.60E-10	q13.3	*STAT6,LRP1*
rs9515203	13	111049623	T	C	0.741	-0.059	0.007	1.50E-18	q34	*COL4A2*
15:48737340_CACATATATAT_C	15	48737340	CACATATATAT	C	0.908	0.063	0.01	1.50E-10	q21.1	*FBN1*
rs4774437	15	62834113	A	G	0.543	-0.034	0.006	3.00E-09	q22.2	*TLN2*
rs1808435	16	75425819	T	C	0.411	-0.044	0.006	1.30E-13	q23.1	*CFDP1*
rs7500448	16	83045790	A	G	0.75	0.038	0.007	1.60E-08	q23.3	*CDH13*
rs488327	16	88989005	T	C	0.662	0.049	0.006	1.00E-15	q24.3	*CBFA2T3*
rs12051555	17	12447601	A	C	0.917	-0.083	0.011	5.70E-15	p12	*MYOCD*
rs112009052	19	41099501	T	A	0.986	-0.183	0.026	3.20E-12	q13.2	*SHKBP1,LTBP4*
rs1065853	19	45413233	G	T	0.921	0.126	0.011	4.70E-31	q13.32	*TOMM40,APOE, APOC1*

SNP	Chr	Position	EA	NEA	EAF	Beta	SE	*P*	Region	Closest gene
rs6120880	20	33829406	C	G	0.568	-0.035	0.006	4.30E-09	q11.22	*EDEM2,MMP24*
rs5757983	22	40920236	C	G	0.468	-0.035	0.006	1.10E-09	q13.2	*MRTFA*

Chr: Chromosome; EA: Effective allele; NEA: Non-effective allele; EAF: Effective allele frequency.

Table S7. Lead variants in loci associated with $cIMT_{max}$

SNP	Chr	Position	EA	NEA	EAF	Beta	SE	*P*	Region	Closest gene
rs7628630	3	21977337	G	A	0.329	0.034	0.006	4.20E-08	p24.3	*ZNF385D*
rs4611812	3	64699445	C	T	0.584	0.035	0.006	3.70E-09	p14.1	*ADAMTS9*
rs10305838	4	148400256	T	C	0.855	-0.046	0.008	5.50E-09	q31.22	*EDNRA*
rs4235201	4	174677572	G	T	0.759	-0.039	0.007	1.60E-08	q34.1	*HAND2*
rs224805	5	81683739	A	G	0.051	0.079	0.013	2.20E-09	q14.2	*ATP6AP1L*
rs310503	5	82891552	G	T	0.661	-0.037	0.006	2.10E-09	q14.2	*VCAN*
rs342988	7	35467026	C	T	0.278	0.054	0.007	2.10E-16	p14.2	*TBX20*
rs3020263	8	6487449	G	A	0.718	0.049	0.007	9.30E-14	p23.1	*MCPH1*
rs2980478	8	8088230	T	C	0.527	-0.039	0.006	1.10E-10	p23.1	*ZNF705B*
rs11250097	8	10811829	T	C	0.467	0.041	0.006	2.00E-12	p23.1	*XKR6*
rs10110725	8	123415086	T	A	0.543	0.034	0.006	1.30E-08	q24.13	*ZHX2*
rs6470156	8	124579985	G	A	0.721	-0.05	0.007	3.80E-14	q24.13	*FBXO32*
rs11371318	10	28230429	G	GAA	0.509	-0.033	0.006	1.20E-08	p12.1	*ARMC4*
rs2150562	10	30162423	A	G	0.311	0.037	0.006	7.40E-09	p11.23	*SVIL*
rs796784254	11	103669291	T	TTATTGAA	0.295	0.046	0.007	3.10E-12	q22.3	*PDGFD*
rs61930625	12	86167409	C	T	0.755	-0.038	0.007	2.80E-08	q21.31	*RASSF9*
rs9515203	13	111049623	T	C	0.741	-0.056	0.007	9.70E-17	q34	*COL4A2*
rs7176966	15	62817568	A	G	0.536	-0.033	0.006	1.30E-08	q22.2	*TLN2*
rs1808435	16	75425819	T	C	0.411	-0.04	0.006	2.30E-11	q23.1	*CFDP1*
rs488327	16	88989005	T	C	0.662	0.047	0.006	5.40E-14	q24.3	*CBFA2T3*
rs740854	17	12449192	A	G	0.915	-0.074	0.011	2.40E-12	p12	*MYOCD*
rs111689747	19	41333152	G	A	0.988	-0.189	0.028	2.10E-11	q13.2	*CYP2A6*
rs1065853	19	45413233	G	T	0.921	0.13	0.011	7.60E-32	q13.32	*TOMM40,APOE,APOC1*
rs369739453	20	32968411	CT	C	0.563	0.038	0.007	5.60E-09	q11.22	*ITCH*
rs5757983	22	40920236	C	G	0.468	-0.033	0.006	1.60E-08	q13.2	*MRTFA*

Chr: Chromosome; EA: Effective allele; NEA: Non-effective allele; EAF: Effective allele frequency.

Table S8. Variants identified by FINEMAP associated with $cIMT_{mean}$

#	Sentinel SNP	Chr	Position	Gene closest to sentinel SNP	Variants identified by FINEMAP	Gene closest to variants identified by FINEMAP	Coding variants (gene)
**1**	rs222476	2	42659267	*COX7A2L, KCNG3*	rs6736913	*EML4*	NP_061936.3: p.Lys283Gln/Glu/Ter (*EML4*)
**2**	rs6744377	2	102069748	*RFX8*	2:102054690_AT_A	*RFX8*	XP_011510074.1:p.Tyr65Cys/Phe/Ser (*RFX8*)
					rs6543078	*RFX8*	
**3**	2:216300905_ACCCG_A	2	216300905	*FN1*	2:216300905_ACCCG_A	*FN1*	
**4**	rs1553085	3	21976118	*ZNF385D*	rs1553085	*ZNF385D*	
**5**	rs6795735	3	64705365	*ADAMTS9*	rs67311847	*ADAMTS9*	
					rs71300731	*ADAMTS9*	
**6**	rs10305838	4	148400256	*EDNRA*	rs10305838	*EDNRA*	
**7**	rs4235201	4	174677572	*HAND2*	rs4235201	*HAND2*	
**8**	rs224805	5	81683739	*ATP6AP1L*	rs226208	*ATP6AP1L*	
					rs224805	*ATP6AP1L*	
					rs7702764	*ATP6AP1L*	
**9**	rs310503	5	82891552	*VCAN*	rs310503	*VCAN*	
					rs1563553	*HAPLN1*	
**10**	rs72801051	5	142685670	*NR3C1*	rs258811	*ARHGAP26*	
					rs72801051	*NR3C1*	NP_001018084.1:p.Asn766Lys (*NR3C1*)
**11**	rs11242713	6	1668142	*GMDS*	rs11242713	*GMDS*	
					rs9392347	*GMDS*	
**12**	rs342988	7	35467026	*TBX20*	rs342988	*TBX20*	
**13**	rs3020263	8	6487449	*MCPH1*	rs3020263	*MCPH1*	
**14**	rs2980478	8	8088230	*ZNF705B*	rs2980478	*ZNF705B*	
**15**	rs4314618	8	10816772	*XKR6*	rs4314618	*XKR6*	
**16**	rs9632837	8	81318125	*ZBTB10*	rs9632837	*ZBTB10*	
**17**	rs10110725	8	123415086	*ZHX2*	8:123413770_GA_G	*ZHX2*	
**18**	rs6470156	8	124579985	*FBXO32*	rs6470156	*FBXO32*	

#	SNP	Chr	Position	Gene closest to sentinel SNP	Variants identified by FINEMAP	Gene closest to variants identified by FINEMAP	Coding variants (gene)
**19**	rs10817556	9	116775859	*ZNF618*	rs10982031	*ZNF618*	NP_001304969.1:p.Tyr115Ter (*ZNF618*), NP_001304969.1:p.Cys299Ter/Trp (*ZNF618*)
**20**	rs11371318	10	28230429	*ARMC4*	rs11371318	*ARMC4*	NP_060546.2:p.Asn686Lys (*ARMC4*)
**21**	rs914279	10	30170487	*JCAD*	rs914279	*JCAD*	NP_065899.1:p.His668Gln (*JCAD*)
					rs9337951	*JCAD*	
**22**	rs796784254	11	103669291	*PDGFD*	rs796784254	*PDGFD*	
					rs36179007	*PDGFD*	
**23**	rs11172113	12	57527283	*STAT6, LRP1*	rs11172113	*LRP1*	NP_005529.1:p.Arg322Gln/Pro/Leu (*INHBC*)
					rs7484541	*R3HDM2*	
**24**	rs61930625	12	86167409	*RASSF9*	rs17345028	*RASSF9*	
**25**	rs9515203	13	111049623	*COL4A2*	rs12429386	*COL4A1*	
					rs9515203	*COL4A2*	
**26**	15:48737340_CACATATATATAT_C	15	48737340	*FBN1*	rs1678982	*FBN1*	
**27**	rs4774437	15	62834113	*TLN2*	rs12441323	*TLN2*	
**28**	rs1808435	16	75425819	*CFDP1*	rs4888367	*BCAR1*	
					rs35633915	*CFDP1*	
**29**	rs7500448	16	83045790	*CDH13*	rs7500448	*CDH13*	
					rs8059012	*CDH13*	
**30**	rs488327	16	88989005	*CBFA2T3*	rs488327	*CBFA2T3*	
**31**	rs12051555	17	12447601	*MYOCD*	rs4442861	*MAP2K4*	
					rs12051555	*MYOCD*	
					rs35712872	*MYOCD*	
					rs12150137	*MYOCD*	
**32**	rs112009052	19	41099501	*SHKBP1, LTBP4*	rs112009052	*LTBP4*	

#	SNP	Chr	Position	Gene closest to sentinel SNP	Variants identified by FINEMAP	Gene closest to variants identified by FINEMAP	Coding variants (gene)
**33**	rs1065853	19	45413233	*TOMM40, APOE, APOC1*	rs1065853	*APOE*	NP_001289617.1:p.Arg202 Cys/Gly/Ser (*APOE*)
					rs56131196	*APOC1*	
**34**	rs6120880	20	33829406	*EDEM2, MMP24*	rs6120880	*MMP24*	
**35**	rs5757983	22	40920236	*MRTFA*	rs5757983	*MRTFA*	

Chr: Chromosome; #: Locus number. Coding variants (gene): coding variants that are in high LD (r^2>0.8) with the variant identified by FINEMAP.

Table S9. cIMT loci with sex difference

Chr	SNP	EAF_{female}	EAF_{male}	INFO	$Beta_{female}$	SE_{female}	P_{female}	$Beta_{male}$	SE_{male}	P_{male}	T_{int}	$P_{sexdiff}$	Gene
							**$cIMT_{min}$**						
8	rs1834451	0.61	0.62	0.99	0.020	0.008	0.0096	0.069	0.010	**7.3E-13**	-3.98	**6.81E-05**	*MCPH1*
							**$cIMT_{mean}$**						
8	rs12543564	0.67	0.67	0.97	0.020	0.008	0.0084	0.066	0.010	**2E-11**	-3.67	**2.44E-04**	*MCPH1*
8	rs71217283	0.58	0.59	0.91	-0.016	0.008	0.061	-0.058	0.010	**2.2E-09**	3.42	**6.36E-04**	*ZNF705B*
8	rs10100333	0.39	0.39	0.98	0.009	0.008	0.22	0.053	0.009	**1.5E-08**	-3.69	**2.23E-04**	*SOX7*
8	rs745379	0.50	0.49	1.00	0.008	0.008	0.33	0.053	0.009	**7.8E-09**	-3.87	**1.09E-04**	*GATA4,C8orf49*
14	rs1187729	0.58	0.59	1.00	-0.007	0.008	0.33	0.051	0.009	**3.3E-08**	-4.93	**8.35E-07**	*SYNE3*
							**$cIMT_{max}$**						
5	rs7447467	0.51	0.51	0.99	-0.043	0.008	**3.5E-08**	-0.005	0.009	0.62	-3.27	**1.08E-03**	*HAPLN1*
8	rs71217283	0.58	0.59	0.91	-0.013	0.008	0.1	-0.058	0.010	**1.9E-09**	3.59	**3.35E-04**	*ZNF705B*
8	rs7005905	0.38	0.38	0.98	0.008	0.008	0.29	0.051	0.009	**4.5E-08**	-3.52	**4.30E-04**	*SOX7*
8	rs745379	0.50	0.49	1.00	0.008	0.008	0.31	0.051	0.009	**2.6E-08**	-3.64	**2.71E-04**	*GATA4,C8orf49*
17	rs80019927	0.75	0.75	0.95	-0.052	0.009	**1.7E-08**	-0.003	0.011	0.81	-3.54	**3.96E-04**	*MAP2K4*

Chr: Chromosome; EA: Effective allele; NEA: Non-effective allele; EAF: Effective allele frequency; Tint: t test statistics of the interaction analysis; $P_{sexdiff}$: *P* for sex interaction test.

Table S10. Cross-tissue TWAS associations at $P < 3.33×10^{-6}$

Gene	Chr	$cIMT_{min}$ Test score	$cIMT_{min}$ *P*	$cIMT_{mean}$ Test score	$cIMT_{mean}$ *P*	$cIMT_{max}$ Test score	$cIMT_{max}$ *P*
ASAP2	2	13.66	**8.32E-07**	8.96	1.01E-04	5.00	6.52E-03
SH3RF3	2	6.37	1.20E-03	12.21	**2.54E-06**	11.00	9.03E-06
XRCC4	5	13.27	**1.38E-06**	14.74	**3.08E-07**	14.45	**4.12E-07**
MFHAS1	8	21.24	**3.11E-10**	20.75	**5.03E-10**	17.20	**1.73E-08**
ERI1	8	25.31	**4.44E-12**	23.25	**2.43E-11**	19.47	**8.06E-10**
PPP1R3B	8	21.37	**3.98E-10**	20.34	**1.18E-09**	16.11	**9.26E-08**
TNKS	8	17.13	**2.12E-08**	17.32	**1.74E-08**	14.99	**2.06E-07**
MSRA	8	19.04	**2.30E-09**	17.46	**1.06E-08**	14.17	**3.22E-07**
SOX7	8	156.95	**8.40E-11**	29.85	**1.13E-13**	23.04	**1.03E-10**
PINX1	8	359.34	**1.81E-11**	361.45	**1.81E-11**	311.12	**1.81E-11**
XKR6	8	223.65	**2.18E-11**	215.65	**2.18E-11**	186.46	**2.19E-11**
MTMR9	8	28.41	**7.19E-13**	28.97	**4.35E-13**	23.63	**5.72E-11**
SLC35G5	8	392.60	**1.78E-11**	384.30	**1.78E-11**	25.69	**4.26E-12**
C8orf12	8	17.10	**1.67E-08**	17.29	**1.38E-08**	15.03	**1.37E-07**
FAM167A	8	26.74	**5.60E-12**	27.95	**2.09E-12**	22.84	**1.47E-10**
BLK	8	84.07	**1.10E-11**	85.90	**1.09E-11**	29.99	**4.11E-14**
GATA4	8	18.11	**1.43E-08**	16.49	**7.45E-08**	14.31	**6.82E-07**
C8orf49	8	12.50	**1.79E-06**	13.39	**7.27E-07**	11.30	5.99E-06
NEIL2	8	16.59	**1.24E-08**	16.42	**1.49E-08**	13.11	**5.22E-07**
DEFB134	8	26.70	**1.31E-12**	28.99	**1.37E-13**	25.48	**4.33E-12**
FAM86B1	8	17.25	**8.50E-09**	18.08	**3.76E-09**	15.45	**5.28E-08**
FAM86B2	8	24.66	**1.26E-11**	29.00	**3.79E-13**	22.91	**5.50E-11**
GTF2E2	8	13.31	**1.45E-06**	12.21	4.66E-06	8.91	1.57E-04
SFRP5	10	13.16	**2.02E-06**	9.96	5.07E-05	8.84	1.60E-04
ARHGAP42	11	12.70	**1.11E-06**	15.46	**6.03E-08**	11.62	3.58E-06
TMEM133	11	10.05	1.73E-05	13.46	**4.58E-07**	10.73	8.61E-06
PDGFD	11	16.96	**2.49E-08**	24.11	**1.82E-11**	26.84	**1.09E-12**
COQ10A	12	21.33	**5.40E-10**	23.17	**8.83E-11**	18.64	**7.46E-09**
LRP1	12	30.64	**5.44E-14**	22.97	**7.32E-11**	12.39	3.12E-06
TLN2	15	13.02	**1.41E-06**	16.52	**4.02E-08**	13.41	**9.47E-07**
RFWD3	16	11.68	**2.84E-06**	10.03	1.57E-05	8.05	1.18E-04
LDHD	16	20.17	**1.92E-09**	19.30	**4.44E-09**	13.02	**1.97E-06**
BCAR1	16	28.98	**6.60E-13**	308.52	**3.04E-11**	22.41	**1.34E-10**
CFDP1	16	23.10	**2.49E-11**	421.76	**2.12E-11**	19.78	**4.99E-10**
TMEM170A	16	256.66	**1.05E-11**	274.30	**1.05E-11**	23.49	**3.37E-11**
CDH13	16	22.01	**2.04E-10**	26.97	**1.38E-12**	22.75	**9.67E-11**
MVD	16	19.01	**3.87E-09**	20.39	**9.40E-10**	15.77	**1.04E-07**
CBFA2T3	16	28.63	**1.04E-13**	27.85	**2.27E-13**	25.68	**2.06E-12**
KANK2	19	9.71	3.42E-05	12.39	**2.28E-06**	13.14	**1.08E-06**
PVRL2	19	10.76	1.43E-05	14.97	**2.04E-07**	16.36	**5.07E-08**
ZNF296	19	11.66	8.52E-06	16.71	**6.78E-08**	15.46	**2.22E-07**
DMPK	19	9.54	2.29E-05	12.25	**1.44E-06**	10.55	8.23E-06
DMWD	19	7.85	1.90E-04	11.92	**2.71E-06**	10.16	1.76E-05

Gene	Chr	$cIMT_{min}$		$cIMT_{mean}$		$cIMT_{max}$	
		**Test score**	***P***	**Test score**	***P***	**Test score**	***P***
ZNF341	20	11.33	7.34E-06	12.32	**2.54E-06**	11.08	9.55E-06
ITCH	20	12.46	3.82E-06	13.77	**9.56E-07**	13.47	**1.32E-06**
MMP24	20	14.07	**4.79E-07**	15.88	**7.72E-08**	13.80	**6.27E-07**
FAM83C	20	15.49	**2.21E-08**	13.73	**1.46E-07**	12.36	**6.14E-07**
GDF5OS	20	12.54	3.67E-06	12.36	4.42E-06	13.11	**2.09E-06**
GDF5	20	14.54	**3.58E-07**	13.76	**8.32E-07**	13.00	**1.87E-06**
RBM12	20	171.47	**6.53E-11**	184.61	**2.97E-11**	23.61	**3.19E-11**
NFS1	20	19.12	**2.37E-09**	20.02	**9.11E-10**	19.12	**2.36E-09**
ST13	22	12.90	**1.57E-06**	15.68	**7.83E-08**	13.22	**1.12E-06**
EP300	22	11.67	4.22E-06	14.83	**1.33E-07**	11.95	3.11E-06

The test statistics of 44 tissues was combined using the Generalized Berk-Jones test to derive the test score. Chr: Chromosome.

Table S11. Result of single-tissue TWAS in arterial tissues for $cIMT_{mean}$

Gene	Chromosome	$P_{artery\ aorta}$	$P_{artery\ coronary}$	$P_{artery\ tibial}$
MRAS	3	1.7E-05	1.0E-05	**1.8E-06**
FER	5	**2.3E-06**	1.3E-05	4.1E-05
ERI1	8	**2.7E-08**	**3.4E-08**	**1.2E-10**
FAM167A	8	**2.0E-06**	NA	NA
FAM86B1	8	1.9E-05	**1.6E-07**	1.5E-02
FAM86B2	8	2.0E-04	**3.7E-08**	**3.2E-06**
MSRA	8	**4.1E-06**	**1.1E-08**	**6.0E-07**
NEIL2	8	1.4E-05	4.3E-04	**8.4E-08**
PINX1	8	1.9E-01	NA	**3.5E-12**
PPP1R3B	8	NA	NA	**9.0E-07**
SLC35G5	8	**1.7E-11**	**5.0E-08**	**1.1E-09**
SOX7	8	2.1E-05	1.5E-03	**1.4E-09**
SVIL	10	**1.2E-06**	9.8E-02	6.8E-05
ARHGAP42	11	**1.4E-07**	**3.7E-07**	**1.0E-07**
TMEM133	11	**7.8E-08**	**8.1E-08**	**6.4E-08**
LRP1	12	**7.1E-09**	**1.1E-08**	**1.6E-10**
TLN2	15	**3.5E-08**	2.5E-05	**1.6E-06**
BCAR1	16	**1.2E-12**	1.0E-05	**3.1E-12**
CBFA2T3	16	**1.1E-08**	1.8E-05	6.7E-01
CDH13	16	**8.1E-12**	**1.7E-07**	**2.3E-12**
CFDP1	16	4.1E-01	**6.1E-12**	**3.4E-06**
NDRG4	16	**2.4E-06**	NA	**2.4E-06**
ZNF469	16	8.5E-01	6.7E-01	**3.1E-06**
APOE	19	6.3E-01	**1.9E-07**	1.7E-04
DMPK	19	**1.1E-06**	**9.7E-07**	**9.4E-07**
DMWD	19	**3.1E-06**	**2.4E-06**	**1.9E-06**
RBM12	20	1.6E-01	**1.4E-09**	6.1E-03
ARVCF	22	**1.3E-06**	7.6E-06	1.4E-05
EP300	22	NA	**5.6E-07**	8.7E-06
ST13	22	NA	**1.6E-08**	2.3E-01

Marked in bold: significant associations passing respective Bonferroni corrected thresholds.

Table S12. Genes associated with cIMT via SMR analysis of the cIMTmean GWAS summary statistics and the eQTL summary statistics from GTEx.

Probe ID	Chr	Gene	Top SNP	A1	A2	Freq	P_{GWAS}	P_{eQTL}	P_{SMR}	P_{HEIDI}
**Artery Aorta**										
ENSG00000253893.2	8	*FAM85B*	rs2945886	G	C	0.44094	1.50E-08	1.17E-14	6.82E-06	6.18E-02
ENSG00000197321.10	10	*SVIL*	rs10826719	A	G	0.58167	9.40E-10	2.56E-12	4.32E-06	4.41E-01
ENSG00000165895.13	11	*ARHGAP42*	rs604723	C	T	0.71758	1.10E-07	7.32E-21	3.22E-06	1.56E-01
ENSG00000170647.2	11	*TMEM133*	rs604723	C	T	0.71758	1.10E-07	4.25E-16	7.51E-06	2.08E-02
ENSG00000171914.10	15	*TLN2*	rs956006	T	C	0.33367	1.10E-08	8.39E-19	2.49E-06	4.61E-01
ENSG00000050820.12	16	*BCAR1*	rs4887824	C	T	0.58773	3.70E-13	3.00E-15	9.89E-08	6.98E-01
ENSG00000140945.11	16	*CDH13*	rs7500448	G	A	0.25238	1.60E-08	1.10E-14	5.77E-06	7.66E-01
**Artery Coronary**										
ENSG00000255310.2	8	*AF131215.2*	rs6996846	G	C	0.53497	6.40E-11	1.09E-09	1.10E-05	1.74E-01
ENSG00000170647.2	11	*TMEM133*	rs633185	C	G	0.71003	9.10E-08	1.58E-11	2.38E-05	9.10E-01
**Artery Tibial**										
ENSG00000253893.2	8	*FAM85B*	rs2955581	C	G	0.45954	1.00E-10	2.00E-20	1.37E-07	1.67E-01
ENSG00000255310.2	8	*AF131215.2*	rs2061830	G	C	0.48864	9.70E-12	8.48E-11	3.16E-06	6.16E-01
ENSG00000079459.8	8	*FDFT1*	rs1736057	A	G	0.57633	4.30E-09	1.44E-37	1.76E-07	1.97E-01
ENSG00000197321.10	10	*SVIL*	rs1571759	C	T	0.58211	7.40E-10	1.43E-19	3.80E-07	3.21E-01
ENSG00000165895.13	11	*ARHGAP42*	rs604723	C	T	0.71758	1.10E-07	1.82E-30	1.17E-06	6.21E-01
ENSG00000170647.2	11	*TMEM133*	rs604723	C	T	0.71758	1.10E-07	2.06E-34	8.91E-07	7.35E-01
ENSG00000123384.9	12	*LRP1*	rs11172113	C	T	0.40633	2.60E-10	1.92E-17	3.43E-07	1.91E-01
ENSG00000050820.12	16	*BCAR1*	rs3784935	G	T	0.59185	1.10E-11	2.06E-23	2.14E-08	1.80E-01

Chr: probe chromosome, A1: effect allele of the top SNP, A2: other allele; Freq: frequency of the A1 of the top SNP, P_{GWAS}: p-value from GWAS, P_{eQTL} : p-value from eQTL study, P_{SMR} :p-value from SMR analysis and P_{HEIDI} : p-value from HEIDI test. A total of 4705, 1975 and 6305 genes were tested in artery aorta, artery coronary and artery tibial tissues respectively. The corresponding significance levels (P_{SMR}) after correcting for multiple testing for SMR analyses were 1.06×10^{-5}, 2.53×10^{-5} and 7.93×10^{-6} respectively. The significance level for rejecting HEIDI test (P_{HEIDI})were 0.05/8=0.00625, 0.05/3=0.0167 and 0.05/9=0.00556 respectively; One gene RP11-252K23.2 on chromosome 16 was filtered out in all three tissues based on HEIDI test.

Table S13. Associations in MAGMA gene-based analysis.

Gene identifier	Gene symbol	Chr	$cIMT_{min}$	$cIMT_{mean}$	$cIMT_{max}$
ENSG00000152518	*ZFP36L2*	2	1.06E-05	**1.91E-06**	4.85E-06
ENSG00000196460	*RFX8*	2	**1.74E-08**	**1.89E-08**	**1.19E-06**
ENSG00000151617	*EDNRA*	4	**2.88E-07**	**5.12E-08**	**1.44E-06**
ENSG00000152348	*ATG10*	5	**8.18E-07**	**2.31E-06**	3.74E-05
ENSG00000186468	*RPS23*	5	**2.26E-07**	**5.56E-07**	9.96E-06
ENSG00000205464	*ATP6AP1L*	5	**2.10E-11**	**2.35E-11**	**4.27E-09**
ENSG00000113580	*NR3C1*	5	3.37E-05	**2.38E-06**	1.84E-05
ENSG00000164463	*CREBRF*	5	**1.89E-06**	**7.82E-08**	**2.78E-07**
ENSG00000113734	*BNIP1*	5	**1.65E-06**	**8.21E-08**	**5.56E-07**
ENSG00000112699	*GMDS*	6	2.74E-06	**1.08E-07**	**6.97E-07**
ENSG00000182611	*HIST1H2AJ*	6	1.07E-05	**2.38E-06**	5.22E-05
ENSG00000184357	*HIST1H1B*	6	7.18E-06	**1.42E-06**	7.15E-05
ENSG00000147316	*MCPH1*	8	**4.56E-10**	**1.33E-07**	**4.03E-08**
ENSG00000147324	*MFHAS1*	8	**1.28E-14**	**5.72E-14**	**4.26E-12**
ENSG00000104626	*ERI1*	8	**1.71E-10**	**3.71E-10**	**1.01E-08**
ENSG00000173281	*PPP1R3B*	8	**7.00E-08**	**4.78E-07**	3.44E-05
ENSG00000173273	*TNKS*	8	**8.89E-07**	**2.38E-06**	2.08E-05
ENSG00000175806	*MSRA*	8	**1.25E-11**	**1.05E-11**	**2.79E-10**
ENSG00000183638	*RP1L1*	8	**8.68E-11**	**3.93E-11**	**1.25E-09**
ENSG00000171056	*SOX7*	8	**7.60E-09**	**1.89E-08**	**4.54E-07**
ENSG00000258724	*SOX7*	8	**5.30E-08**	**1.16E-07**	**1.90E-06**
ENSG00000254093	*PINX1*	8	**3.42E-08**	**8.71E-08**	**1.57E-06**
ENSG00000171044	*XKR6*	8	**3.09E-16**	**6.39E-16**	**2.30E-13**
ENSG00000215346	*AF131215.5*	8	**1.28E-10**	**3.24E-10**	**8.28E-09**
ENSG00000104643	*MTMR9*	8	**6.46E-10**	**2.22E-10**	**3.11E-09**
ENSG00000177710	*SLC35G5*	8	**4.60E-07**	**3.01E-07**	**2.51E-06**
ENSG00000184608	*C8orf12*	8	**5.24E-11**	**4.55E-11**	**5.00E-10**
ENSG00000154319	*FAM167A*	8	**3.78E-09**	**4.24E-09**	**5.48E-08**
ENSG00000136573	*BLK*	8	**8.20E-12**	**6.43E-12**	**8.71E-11**
ENSG00000136574	*GATA4*	8	**2.89E-11**	**8.39E-11**	**2.49E-09**
ENSG00000255046	*RP11-297N6.4*	8	**1.62E-06**	**1.94E-06**	3.44E-05
ENSG00000205882	*DEFB134*	8	**3.79E-07**	**9.71E-07**	1.82E-05
ENSG00000255098	*RP11-481A20.11*	8	**2.55E-06**	**2.57E-06**	1.13E-05
ENSG00000157657	*ZNF618*	9	**1.06E-06**	4.74E-05	1.37E-03
ENSG00000169126	*ARMC4*	10	7.85E-05	**1.44E-06**	**1.45E-07**
ENSG00000135517	*MIP*	12	**5.15E-07**	**6.54E-07**	6.46E-06
ENSG00000176422	*SPRYD4*	12	3.43E-06	**2.06E-06**	1.91E-05
ENSG00000135423	*GLS2*	12	**2.30E-06**	**9.08E-07**	**2.41E-06**
ENSG00000198774	*RASSF9*	12	6.06E-06	**1.26E-07**	**1.36E-07**
ENSG00000134871	*COL4A2*	13	**1.74E-07**	**4.11E-07**	2.88E-06
ENSG00000171914	*TLN2*	15	3.74E-05	**1.73E-06**	4.42E-06
ENSG00000166949	*SMAD3*	15	**2.32E-06**	9.25E-06	3.88E-04
ENSG00000129038	*LOXL1*	15	**1.06E-06**	**1.66E-06**	**1.65E-06**
ENSG00000125107	*CNOT1*	16	4.74E-06	**5.14E-07**	**1.75E-06**
ENSG00000153774	*CFDP1*	16	**3.58E-12**	**2.37E-12**	**1.45E-10**
ENSG00000261717	*RP11-77K12.1*	16	**2.65E-11**	**7.80E-12**	**6.22E-10**
ENSG00000166822	*TMEM170A*	16	**4.40E-10**	**1.05E-10**	**5.80E-09**
ENSG00000183196	*CHST6*	16	1.02E-05	**1.38E-06**	7.63E-06
ENSG00000103335	*PIEZO1*	16	**3.66E-07**	**1.69E-06**	8.11E-06
ENSG00000129993	*CBFA2T3*	16	**3.53E-12**	**1.44E-11**	**2.37E-10**
ENSG00000205018	*RP11-830F9.6*	16	**1.46E-13**	**5.17E-13**	**4.44E-12**

Gene identifier	Gene symbol	Chr	$cIMT_{min}$	$cIMT_{mean}$	$cIMT_{max}$
ENSG00000130203	*APOE*	19	**3.33E-16**	**7.55E-11**	**2.22E-16**
ENSG00000130208	*APOC1*	19	**4.96E-07**	**1.40E-07**	**6.87E-08**
ENSG00000078804	*TP53INP2*	20	**1.10E-06**	**1.74E-06**	9.36E-06
ENSG00000088298	*EDEM2*	20	**3.37E-07**	**1.50E-06**	1.16E-05
ENSG00000124126	*PREX1*	20	1.78E-05	**4.13E-07**	**1.85E-06**

Chr: Chromosome. Marked in bold: significant association with $P<2.63\times10^{-6}$.

Table S14. Top ten genes prioritized by DEPICT to be associated with cIMT

Locus	Nr of genes in locus	Chromosome and position	Ensembl Gene ID	Gene symbol	Nominal P value	Gene closest to lead SNP	Gene bio-type	Top cis eQTL SNP (Westra et al. Nature Genetics 2014)	False discovery rate < 5%
				$cIMT_{min}$					
rs12549144;rs13262031;rs7831557	29	chr8:9106927-11726957	ENSG00000136574	*GATA4*	5.10E-06	FALSE	protein coding+ processed transcript+ retained intron	-	Yes
rs342988	1	chr7:35242042-35293758	ENSG00000164532	*TBX20*	7.09E-03	TRUE	protein coding+ retained intron	rs1833130;rs17676885	No
rs914279	1	chr10:30301729-30404423	ENSG00000165757	*JCAD*	7.19E-03	TRUE	protein coding+ processed transcript	-	No
rs12549144;rs13262031;rs7831557	29	chr8:9106927-11726957	ENSG00000171056	*SOX7*	8.02E-03	FALSE	protein coding	rs10092781;rs7001281	No
rs12549144;rs13262031;rs7831557	29	chr8:9106927-11726957	ENSG00000255394	*C8orf49*	2.98E-02	FALSE	protein coding	-	No
rs9515203	1	chr13:110958159-111165374	ENSG00000134871	*COL4A2*	9.68E-02	TRUE	protein coding+ processed transcript	-	No
rs12549144;rs13262031;rs7831557	29	chr8:9106927-11726957	ENSG00000184608	*C8orf12*	1.06E-01	FALSE	processed transcript+ protein coding	-	No
rs12549144;rs13262031;rs7831557	29	chr8:9106927-11726957	ENSG00000173273	*TNKS*	1.07E-01	TRUE	protein coding+ processed transcript+ retained intron+nonsense mediated decay	rs17737611	No
rs2019090	1	chr11:103777914-104035107	ENSG00000170962	*PDGFD*	1.10E-01	TRUE	protein coding	rs481552;rs11226008	No
rs12549144;rs13262031;rs7831557	29	chr8:9106927-11726957	ENSG00000253887	-	1.20E-01	FALSE	lincRNA	-	No
				$cIMT_{mean}$					
rs342988	1	chr7:35242042-35293758	ENSG00000164532	*TBX20*	1.10E-02	TRUE	protein coding+ retained intron	rs1833130;rs17676885	No
rs914279	1	chr10:30301729-30404423	ENSG00000165757	*JCAD*	4.92E-02	TRUE	protein coding+ processed transcript	-	No
rs9329221	17	chr8:9911778-11296167	ENSG00000171056	*SOX7*	5.16E-02	FALSE	protein coding	rs10092781;rs7001281	No
rs224960	1	chr5:81575281-81682796	ENSG00000205464	*ATP6AP1L*	6.75E-02	TRUE	protein coding+ processed transcript+ retained intron	-	No
rs4774437	1	chr15:62682725-63136830	ENSG00000171914	*TLN2*	1.25E-01	TRUE	processed transcript+ protein coding+ retained intron	-	No
rs6795735	1	chr3:64670585-64997143	ENSG00000241684	*ADAMTS9-AS2*	1.37E-01	TRUE	processed transcript+ retained intron	-	No
rs7500448	1	chr16:82660663-83829174	ENSG00000140945	*CDH13*	1.54E-01	TRUE	protein coding	rs16962255;rs6565105	No
rs9329221	17	chr8:9911778-11296167	ENSG00000255310	-	1.79E-01	FALSE	protein coding	-	No
rs9329221	17	chr8:9911778-11296167	ENSG00000184608	*C8orf12*	2.25E-01	FALSE	processed transcript+protein coding	-	No
rs9515203	1	chr13:110958159-111165374	ENSG00000134871	*COL4A2*	2.52E-01	TRUE	protein coding+processed transcript	-	No

Locus	Nr of genes in locus	Chromosome and position	Ensembl Gene ID	Gene symbol	Nominal P value	Gene closest to lead SNP	Gene bio-type	Top cis eQTL SNP (Westra et al. Nature Genetics 2014)	False discovery rate < 5%
					**$cIMT_{max}$**				
rs342988	1	chr7:35242042-35293758	ENSG00000164532	*TBX20*	3.65E-03	TRUE	protein coding+retained intron	rs1833130;rs17676885	No
rs740854	1	chr17:12569207-12672266	ENSG00000141052	*MYOCD*	2.20E-02	TRUE	protein coding+processed transcript	-	No
rs7831557	17	chr8:9911778-11296167	ENSG00000171056	*SOX7*	6.62E-02	FALSE	protein coding	rs10092781;rs7001281	No
rs224960	1	chr5:81575281-81682796	ENSG00000205464	*ATP6AP1L*	6.73E-02	TRUE	protein coding+processed transcript+retained intron	-	No
rs7176966	1	chr15:62682725-63136830	ENSG00000171914	*TLN2*	8.28E-02	TRUE	processed transcript+protein coding+retained intron	-	No
rs4611812	1	chr3:64670585-64997143	ENSG00000241684	*ADAMTS9-AS2*	9.69E-02	TRUE	processed transcript+retained intron	-	No
rs2150562	1	chr10:29746267-30025710	ENSG00000197321	*SVIL*	1.07E-01	TRUE	protein coding+processed transcript	-	No
rs4235201	1	chr4:174818403-174850676	ENSG00000250708	-	1.12E-01	TRUE	lincRNA	-	No
rs7831557	17	chr8:9911778-11296167	ENSG00000255310	-	1.18E-01	FALSE	protein coding	-	No
rs9515203	1	chr13:110958159-111165374	ENSG00000134871	*COL4A2*	3.19E-01	TRUE	protein coding+processed transcript	-	No

Table S15. Gene prioritization results for GWAS loci identified in the UK Biobank

Gene	Chr	Region	SNP-level analysis			Gene-level analysis			
			Closest to the lead variant	Closest to fine-mapped variant	Coding variant in high LD to fine-mapped variant*	Cross-tissue TWAS†	Arterial TWAS	SMR analysis	DEPICT
EML4	2	p21		y	y				
COX7A2L	2	p21	main						
KCNG3	2	p21	main						
ZFP36L2	2	p21							
RFX8	2	q11.2	main	y	y				
FN1	2	q35	main	y					
ZNF385D	3	p24.3	main	y					
ADAMTS9	3	p14.1	main	y					
EDNRA	4	q31.22	main	y					
HAND2	4	q34.1	main	y					
RPS23	5	q14.2							
ATP6AP1L	5	q14.2	main	y					
XRCC4	5	q14.2				y			
VCAN	5	q14.2	main	y					
HAPLN1	5	q14.2	sex interaction (female)	y					
ARHGAP26	5	q31.3		y					
NR3C1	5	q31.3	main	y	y				
GMDS	6	p25.3	main	y					
TBX20	7	p14.2	main	y					
HERPUD2	7	p14.2	male						
MCPH1	8	p23.1	main	y					
FAM85B	8	p23.1						y	
ZNF705B	8	p23.1	main	y					
MFHAS1	8	p23.1				y			
ERI1	8	p23.1				y	y		
PPP1R3B	8	p23.1				y	y		
TNKS	8	p23.1				y			
MSRA	8	p23.1				y	y		
RP1L1	8	p23.1							
SOX7	8	p23.1	sex interaction (male)			y	y		
PINX1	8	p23.1				y	y		

XKR6	8	p23.1	main	y		y			
AF131215.5	8	p23.1						y	
MTMR9	8	p23.1				y			
SLC35G5	8	p23.1				y	y		
C8orf12	8	p23.1				y			
FAM167A	8	p23.1				y	y		
BLK	8	p23.1				y			
GATA4	8	p23.1	sex interaction (male)			y			y
C8orf49	8	p23.1	sex interaction (male)			y			
NEIL2	8	p23.1				y	y		
FDFT1	8	p23.1						y	
DEFB134	8	p23.1				y			
FAM86B1	8	p23.1				y	y		
FAM86B2	8	p23.1				y	y		
ZBTB10	8	q21.13	main	y					
ZHX2	8	q24.13	main	y					
FBXO32	8	q24.13	main	y					
ZNF618	9	q32	main	y	y				
ARMC4	10	p12.1	main	y	y				
SVIL	10	p11.23	main				y	y	
JCAD	10	p11.23	main	y	y				
PDGFD	11	q22.3	main	y		y			
GLS2	12	q13.3							
STAT6	12	q13.3	main						
LRP1	12	q13.3	main	y		y	y		
R3HDM2	12	q13.3		y					
INHBC	12	q13.3			y				
RASSF9	12	q21.31	main	y					
COL4A1	13	q34		y					
COL4A2	13	q34	main	y					
SYNE3	14	q32.13	sex interaction (male)						
FBN1	15	q21.1	main	y					
TLN2	15	q22.2	main	y		y	y	y	
LOXL1	15	q24.1	meta-analysis	y					
RFWD3	16	q23.1				y			
LDHD	16	q23.1				y			
BCAR1	16	q23.1		y		y	y	y	
CFDP1	16	q23.1	main	y					
RP11-77K12.1	16	q23.1							

TMEM170A	16	q23.1				y		
CDH13	16	q23.3	main	y			y	y
MVD	16	q24.2				y		
ZNF469	16	q24.2					y	
CBFA2T3	16	q24.3	main	y			y	
RP11-830F9.6	16	q24.3						
MAP2K4	17	p12	sex interaction (female)	y				
MYOCD	17	p12	main	y				
SHKBP1	19	q13.2	main					
LTBP4	19	q13.2	main	y				
CYP2A6	19	q13.2	main					
PVRL2	19	q13.32				y		
TOMM40	19	q13.32	main					
APOE	19	q13.32	main	y	y		y	
APOC1	19	q13.32	main	y				
ZNF296	19	q13.32				y		
DMPK	19	q13.32					y	
DMWD	19	q13.32					y	
ZNF341	20	q11.22				y		
ITCH	20	q11.22	main			y		
EDEM2	20	q11.22	main					
MMP24	20	q11.22	main	y		y		
FAM83C	20	q11.22				y		
GDF5OS	20	q11.22				y		
GDF5	20	q11.22				y		
RBM12	20	q11.22				y	y	
NFS1	20	q11.22				y		
MRTFA	22	q13.2	main					
ST13	22	q13.2				y	y	
EP300	22	q13.2				y	y	

Genes located within loci identified in the UK Biobank via the main GWAS, sex interaction analysis and meta-analysis were included; Rows with alternate colors indicate different loci. Genes closest to the lead variant included the nearest gene and any additional gene within 10 KB of the lead variant. Chr: Chromosome; *: if there existed non-synonymous variant in LD R^2>0.8 with the fine-mapped variant; †: genes consistently prioritized in all cIMT traits were noted.

Table S16 Top ten results from MAGMA gene-set analyses for three cIMT phenotypes

Gene Set	N_{genes}	Beta	Beta STD	SE	*P*	P_{bon}
**$cIMT_{min}$**						
Curated gene sets:reactome crosslinking of collagen fibrils	16	1.337	0.039	0.258	1.15E-07	**0.002**
Curated gene sets:nikolsky breast cancer 20q11 amplicon	31	1.426	0.057	0.284	2.66E-07	**0.004**
Curated gene sets:reactome signaling by pdgf	58	0.583	0.032	0.127	2.18E-06	**0.034**
GO cc:go collagen type iv trimer	4	2.414	0.035	0.535	3.18E-06	**0.049**
GO bp:go peptidyl lysine oxidation	5	1.984	0.032	0.457	7.26E-06	0.112
GO mf:go protein lysine 6 oxidase activity	5	1.984	0.032	0.457	7.26E-06	0.112
GO cc:go cell substrate junction	395	0.196	0.028	0.045	7.65E-06	0.118
Curated gene sets:ji carcinogenesis by kras and stk11 dn	16	1.017	0.029	0.235	7.81E-06	0.121
GO bp:go regulation of platelet derived growth factor receptor beta signaling pathway	8	1.350	0.027	0.314	8.49E-06	0.131
GO bp:go platelet derived growth factor receptor beta signaling pathway	14	1.068	0.029	0.253	1.22E-05	0.188
**$cIMT_{mean}$**						
Curated gene sets:nikolsky breast cancer 20q11 amplicon	31	1.424	0.057	0.284	2.68E-07	**0.004**
Curated gene sets:reactome crosslinking of collagen fibrils	16	1.279	0.037	0.258	3.65E-07	**0.006**
Curated gene sets:reactome signaling by pdgf	58	0.620	0.034	0.127	4.95E-07	**0.008**
Curated gene sets:nakamura adipogenesis late dn	35	0.697	0.030	0.160	6.70E-06	0.104
GO bp:go artery development	84	0.464	0.031	0.108	9.34E-06	0.145
GO bp:go regulation of low density lipoprotein particle receptor catabolic process	5	1.802	0.029	0.422	9.68E-06	0.150
Curated gene sets:reactome chylomicron clearance	5	1.888	0.030	0.447	1.20E-05	0.185
Curated gene sets:tsai response to radiation therapy	33	0.732	0.030	0.173	1.20E-05	0.185
GO bp:go ameboidal type cell migration	368	0.201	0.027	0.048	1.64E-05	0.253
GO bp:go low density lipoprotein particle remodeling	14	1.069	0.029	0.258	1.76E-05	0.273
**$cIMT_{max}$**						
Curated gene sets:nikolsky breast cancer 20q11 amplicon	31	1.362	0.055	0.280	5.64E-07	**0.009**
Curated gene sets:reactome crosslinking of collagen fibrils	16	1.216	0.035	0.254	8.85E-07	**0.014**
Curated gene sets:reactome signaling by pdgf	58	0.559	0.031	0.125	3.68E-06	0.057
GO bp:go artery development	84	0.478	0.031	0.107	3.80E-06	0.059
Curated gene sets:reactome chylomicron clearance	5	1.959	0.032	0.440	4.31E-06	0.067
GO bp:go regulation of low density lipoprotein particle receptor catabolic process	5	1.795	0.029	0.416	7.90E-06	0.122
GO bp:go low density lipoprotein particle remodeling	14	1.096	0.030	0.254	8.27E-06	0.128
GO bp:go dephosphorylation	451	0.171	0.026	0.041	1.55E-05	0.240
GO bp:go protein containing complex remodeling	30	0.717	0.028	0.174	1.99E-05	0.307
Curated gene sets:hellebrekers silenced during tumor angiogenesis	75	0.444	0.028	0.108	2.02E-05	0.312

P_{bon} : Bonferroni corrected P-value.

Table S17. cIMT variants significantly associated with CAD

cIMT lead SNP	CAD meta-analysis SNP	Chr	Position	Effect	SE	*P*	Gene
rs515135	rs515135	2	21286057	-0.0555	0.0066	**5.74E-17**	*APOB*
rs6795735	rs7428936	3	64710850	-0.019	0.0052	**2.87E-04**	*ADAMTS9*
rs59415853	rs6841581	4	148401190	0.0751	0.0074	**5.18E-24**	*EDNRA*
rs17477177	rs12705390	7	106410777	0.03	0.0062	**1.21E-06**	*PIK3CG*
rs7004066	rs6995692	8	10587008	-0.0189	0.0058	**1.18E-03**	*SOX7*
rs55917128	rs11189523	10	100011077	-0.0268	0.0056	**1.96E-06**	*LOXL4*
rs974819	rs974819	11	103660567	0.0614	0.0055	**1.12E-28**	*PDGFD*
rs11172113	rs11172113	12	57527283	-0.0227	0.0051	**1.04E-05**	*STAT6,LRP1*
rs9515203	rs9515203	13	111049623	0.0596	0.006	**3.89E-23**	*COL4A2*
rs12441130	rs12441130	15	74234902	0.0199	0.0056	**4.13E-04**	*LOXL1*
rs3851740	rs8046696	16	75442143	-0.0479	0.0058	**1.91E-16**	*BCAR/CFDP151*
rs7500448	rs7500448	16	83045790	0.0557	0.0068	**1.61E-16**	*CDH13*
rs7412	rs7412	19	45412079	-0.1368	0.011	**2.14E-35**	*APOE,APOC,TOMM40*
rs8139974	rs139064	22	40886557	0.0215	0.0057	**1.56E-04**	*MRTFA*

Chr: Chromosome; SE: standard error. Marked in bold: significant association after Bonferroni correction with $P<1.19\times10^{-3}$ (0.05/42). Associations of CAD were obtained in a meta-analysis of UK Biobank and CARDIoGRAMplusC4D. If a cIMT lead SNP was not available in the meta-analysis, a proxy SNP in high LD ($R^2>0.8$) was selected.

Supplementary figures

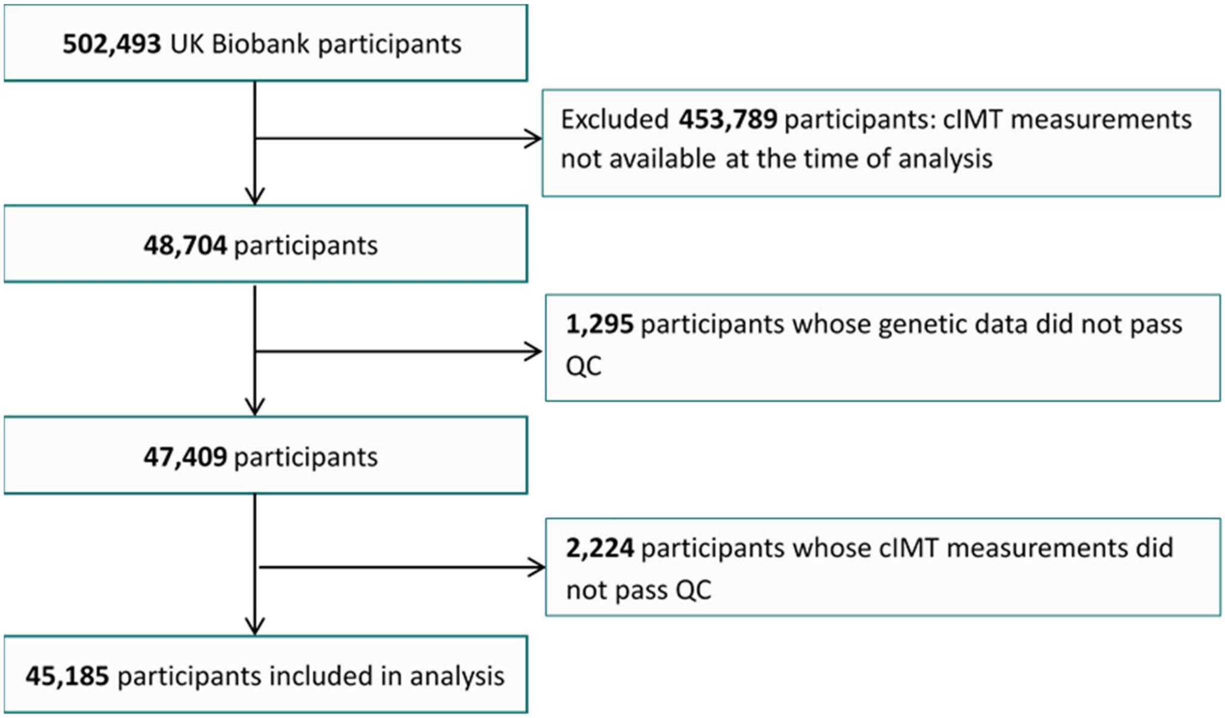

Figure S1. Study population.

GWAS on cIMT measurements were performed in 45,185 participants in the UK Biobank.

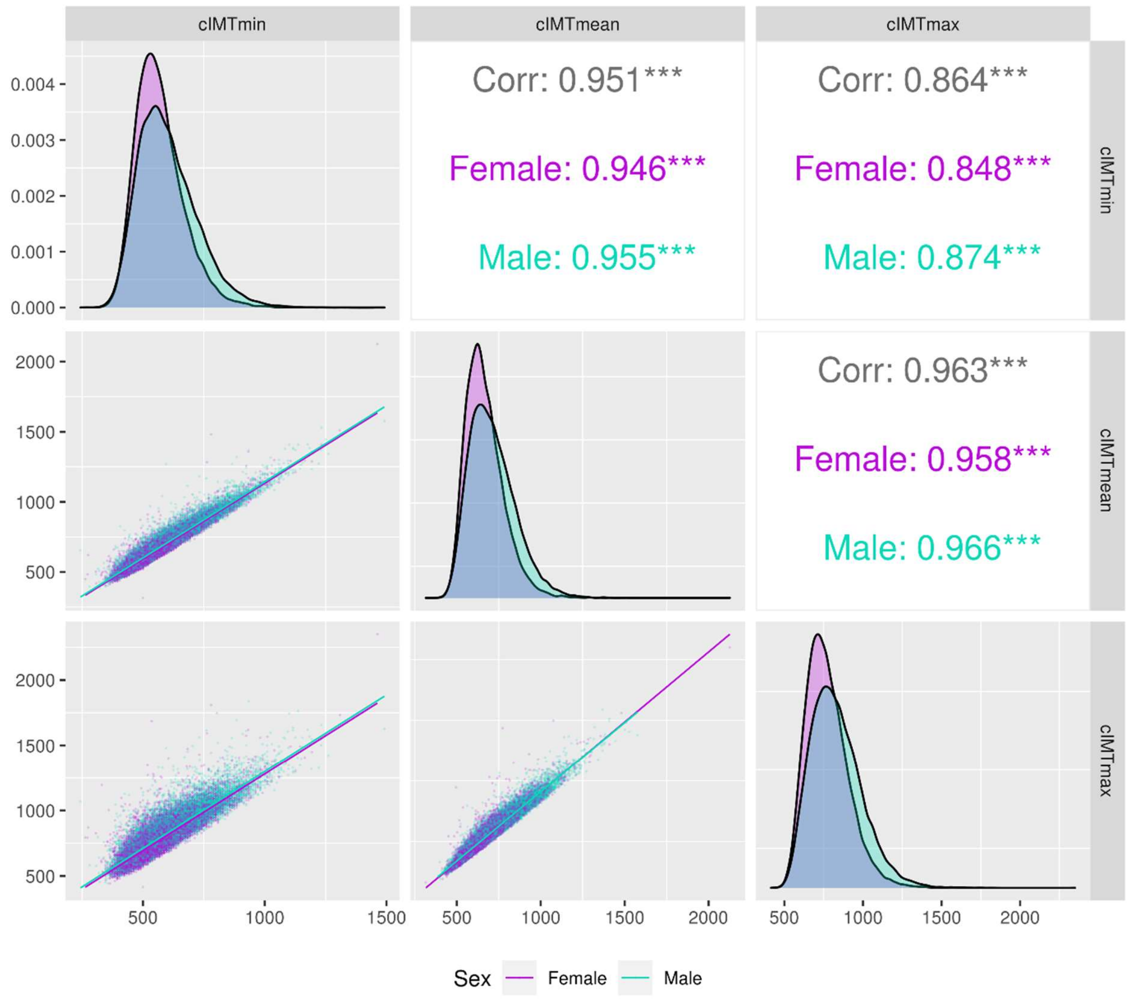

Figure S2. Distribution and correlations Between cIMT measurements. Diagonal: Distribution of $cIMT_{min,}$ $cIMT_{mean,}$ $cIMT_{max}$ stratified by sex; lower triangular panels: scatterplots against cIMT measurements; upper triangular panels: Spearman rank correlations between cIMT measurements in overall sample (Corr) and sex-stratified.

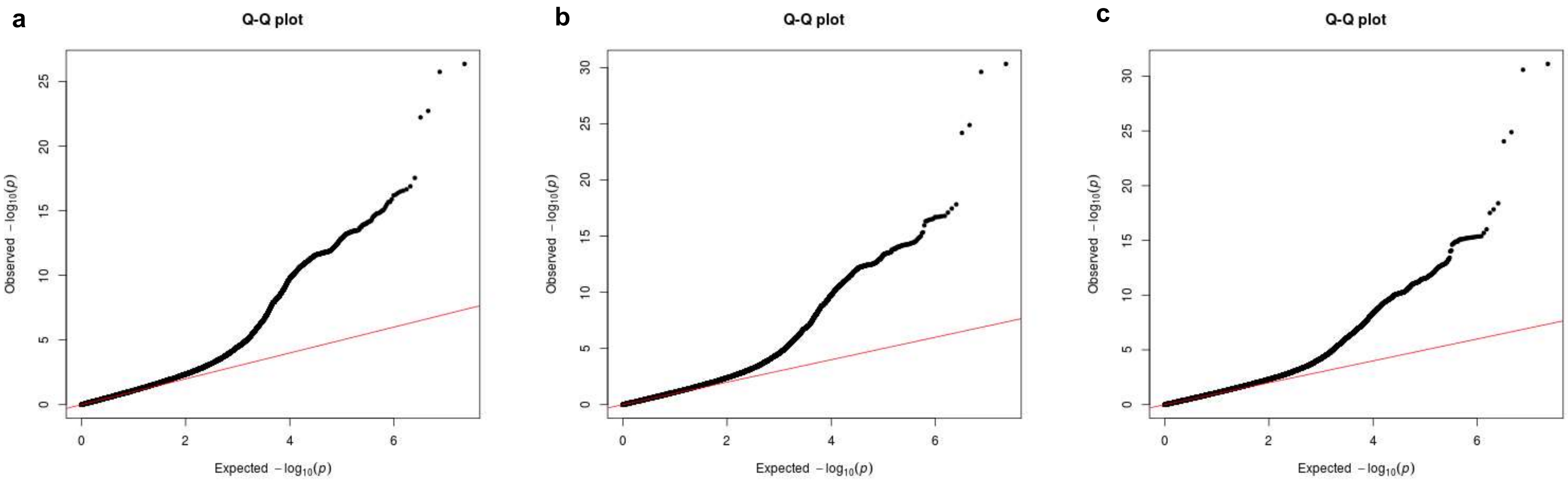

Figure S3. Q-Q plot for the three cIMT measurements

a: cIMT$_{min}$; b: cIMT$_{mean}$; c: cIMT$_{max}$. The genomic inflation factor λ are1.06,1.07 and 1.07 for cIMT$_{min}$, cIMT$_{mean}$ and cIMT$_{max}$ respectively

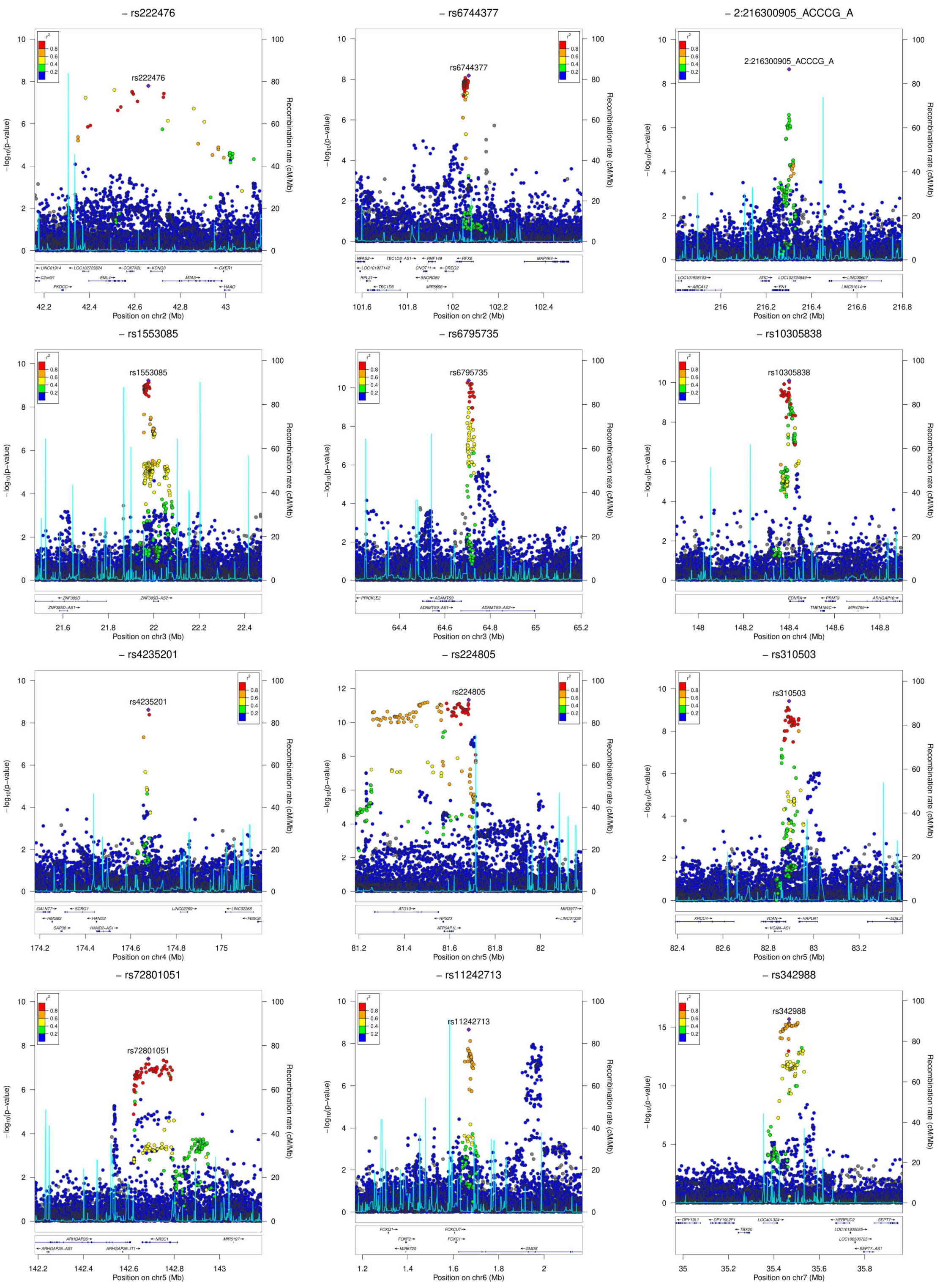

rs222476
rs6744377
2:216300905_ACCCG_A
rs1553085
rs6795735
rs10305838
rs4235201
rs224805
rs310503
rs72801051
rs11242713
rs342988
−log10(p-value)
Recombination rate (cM/Mb)
r2
Position on chr2 (Mb)
Position on chr3 (Mb)
Position on chr4 (Mb)
Position on chr5 (Mb)
Position on chr6 (Mb)
Position on chr7 (Mb)

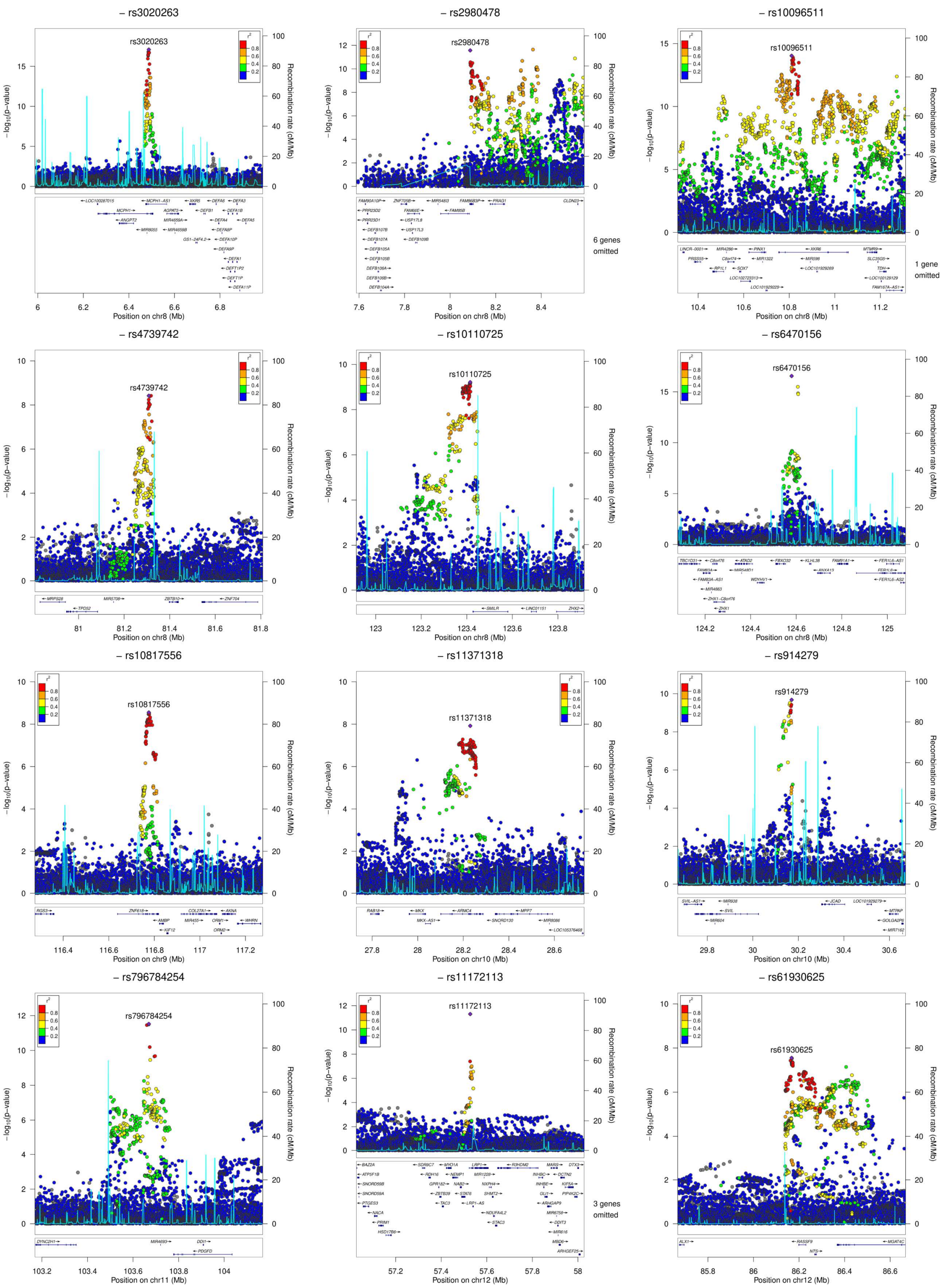
– rs3020263
– rs2980478
– rs10096511
– rs4739742
– rs10110725
– rs6470156
– rs10817556
– rs11371318
– rs914279
– rs796784254
– rs11172113
– rs61930625
−log10(p−value)
Recombination rate (cM/Mb)
Position on chr8 (Mb)
Position on chr9 (Mb)
Position on chr10 (Mb)
Position on chr11 (Mb)
Position on chr12 (Mb)
6 genes omitted
1 gene omitted
3 genes omitted

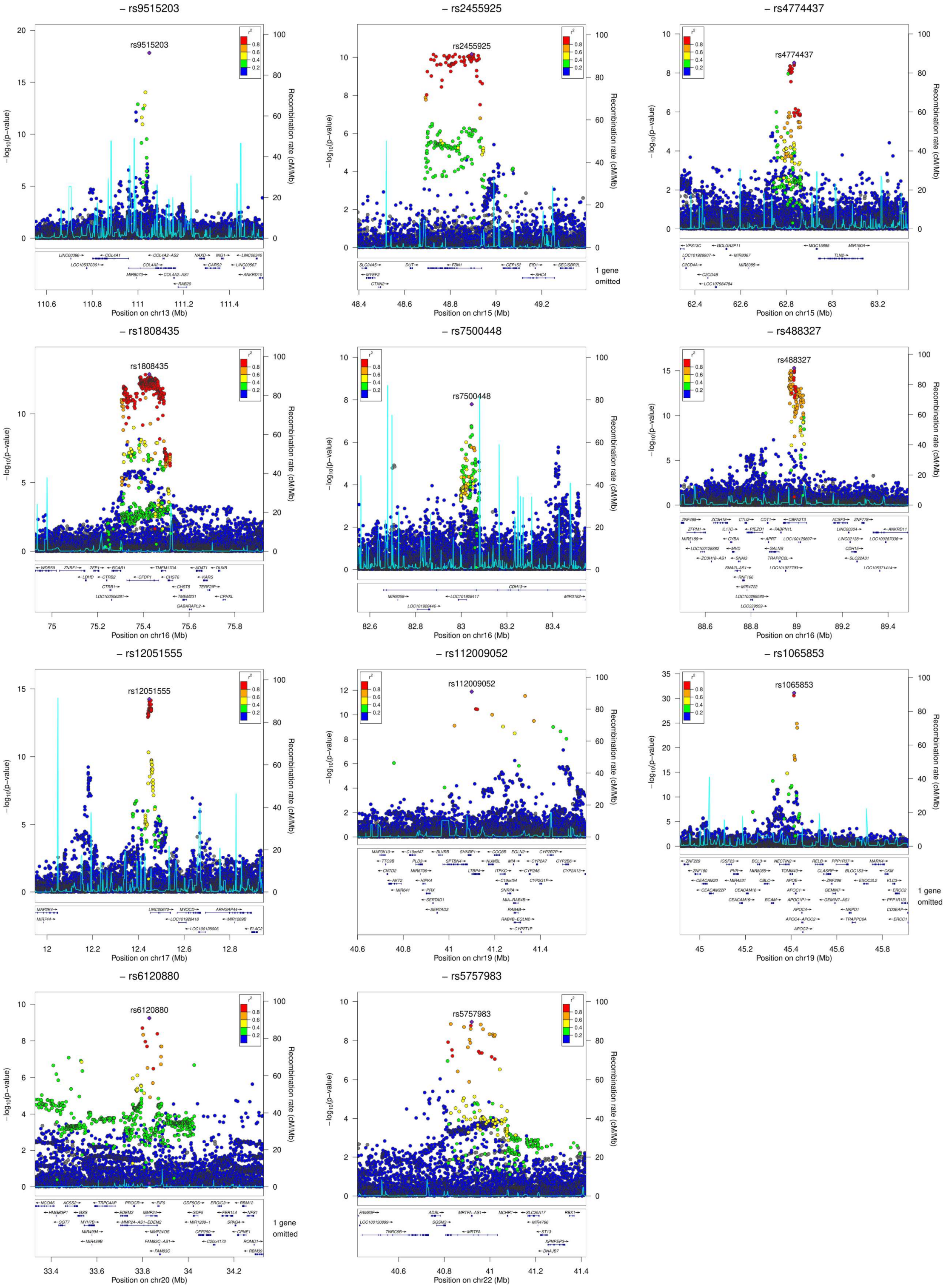

Figure S4. Locus zoom plots for 35 independent cIMT loci.

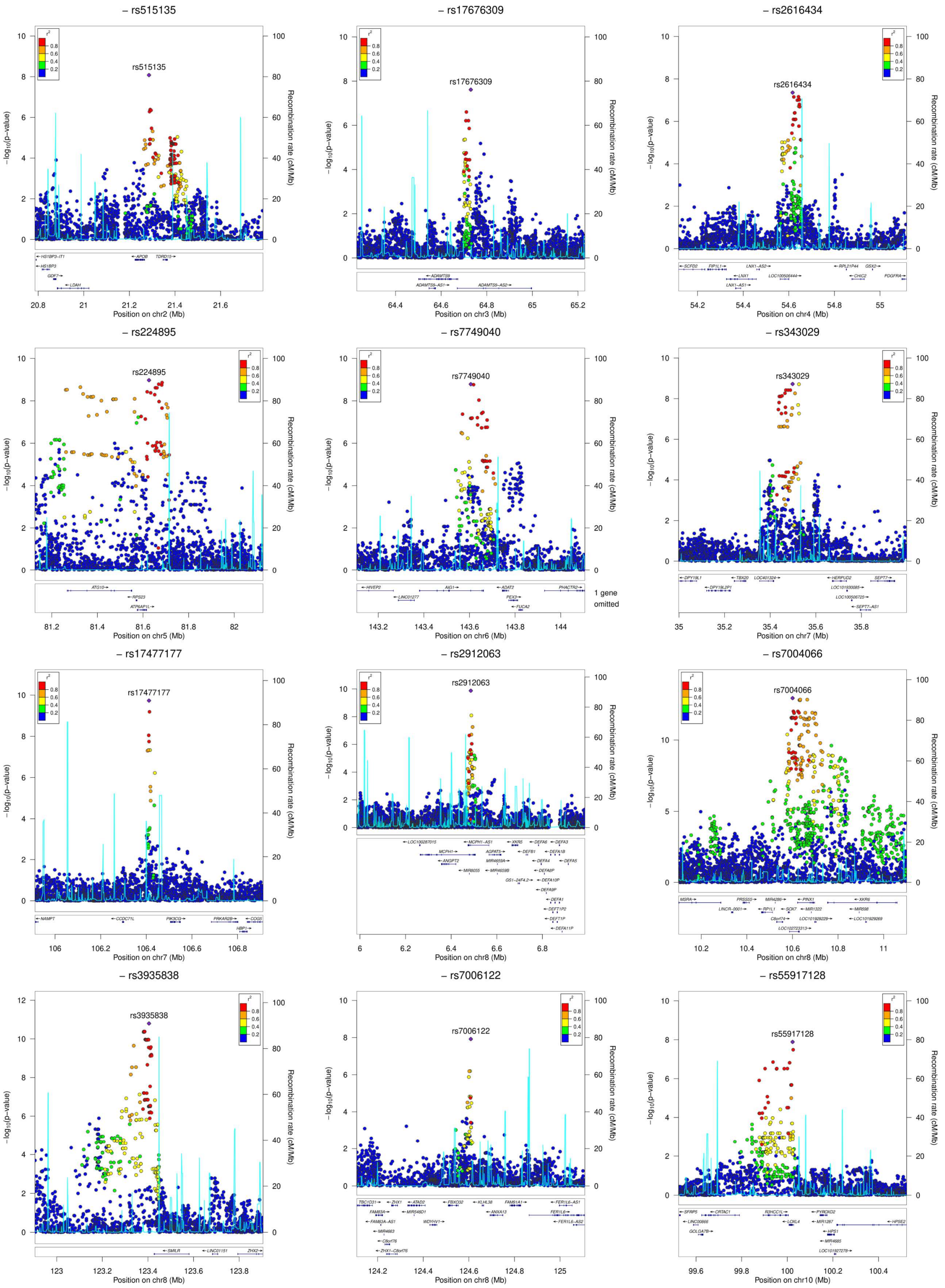

– rs515135
rs515135
Position on chr2 (Mb)
– rs17676309
rs17676309
Position on chr3 (Mb)
– rs2616434
rs2616434
Position on chr4 (Mb)
– rs224895
rs224895
Position on chr5 (Mb)
– rs7749040
rs7749040
1 gene omitted
Position on chr6 (Mb)
– rs343029
rs343029
Position on chr7 (Mb)
– rs17477177
rs17477177
Position on chr7 (Mb)
– rs2912063
rs2912063
Position on chr8 (Mb)
– rs7004066
rs7004066
Position on chr8 (Mb)
– rs3935838
rs3935838
Position on chr8 (Mb)
– rs7006122
rs7006122
Position on chr8 (Mb)
– rs55917128
rs55917128
Position on chr10 (Mb)
–log10(p-value)
Recombination rate (cM/Mb)
r2

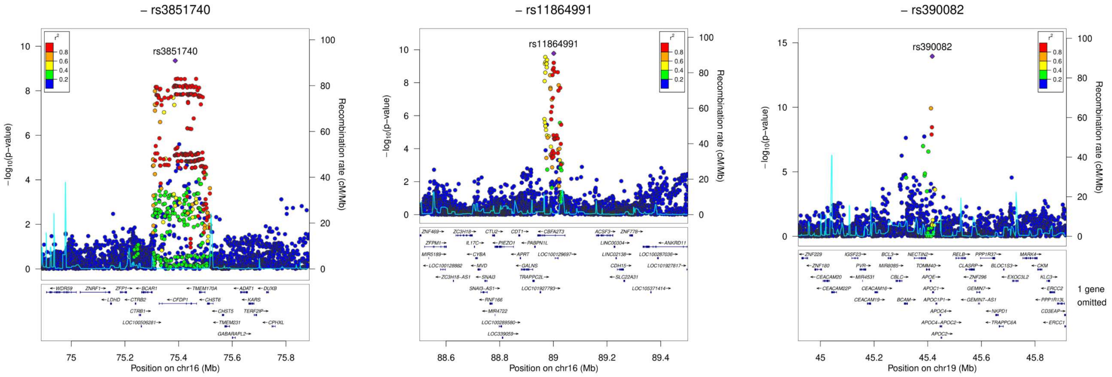

Figure S5. Locus zoom plots for $cIMT_{max}$ loci from the meta-analysis of UK Biobank and CHARGE/UCLEB consortia.

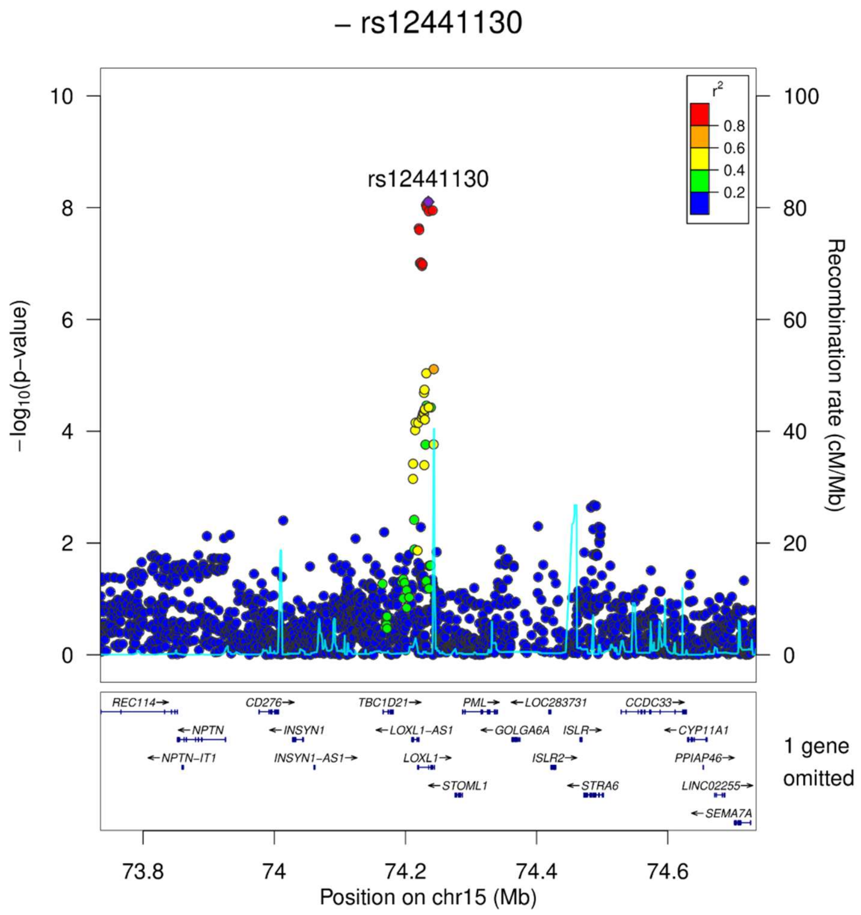

Figure S6. Locus zoom plot for *LOXL1* identified in the meta-analysis of the three cIMT measurements.

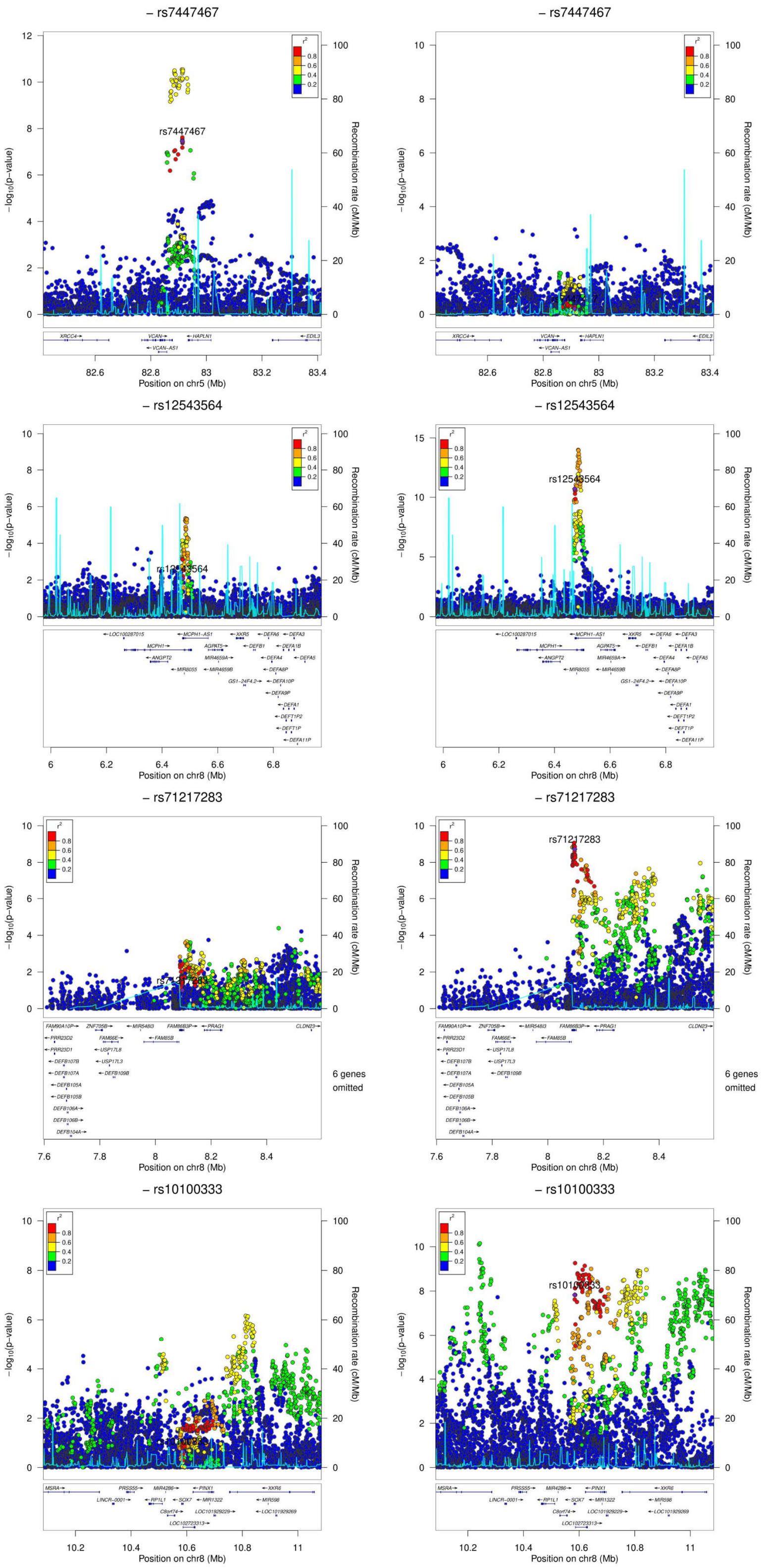
– rs7447467
rs7447467
−log10(p−value)
Recombination rate (cM/Mb)
Position on chr5 (Mb)
XRCC4
VCAN
HAPLN1
VCAN-AS1
EDIL3
– rs12543564
rs12543564
Position on chr8 (Mb)
LOC100287015
MCPH1-AS1
XKR5
DEFA6
DEFA3
MCPH1
AGPAT5
DEFB1
DEFA1B
ANGPT2
MIR4659A
DEFA4
DEFA5
MIR8055
MIR4659B
DEFA8P
GS1−24F4.2
DEFA10P
DEFA9P
DEFA1
DEFT1P2
DEFT1P
DEFA11P
– rs71217283
rs71217283
FAM90A10P
ZNF705B
MIR548I3
FAM86B3P
PRAG1
CLDN23
PRR23D2
FAM66E
FAM85B
PRR23D1
USP17L8
DEFB107B
USP17L3
DEFB107A
DEFB109B
DEFB105A
DEFB105B
DEFB106A
DEFB106B
DEFB104A
6 genes omitted
– rs10100333
rs10100333
MSRA
PRSS55
MIR4286
PINX1
XKR6
LINCR−0001
RP1L1
SOX7
MIR1322
MIR598
C8orf74
LOC101929229
LOC101929269
LOC102723313

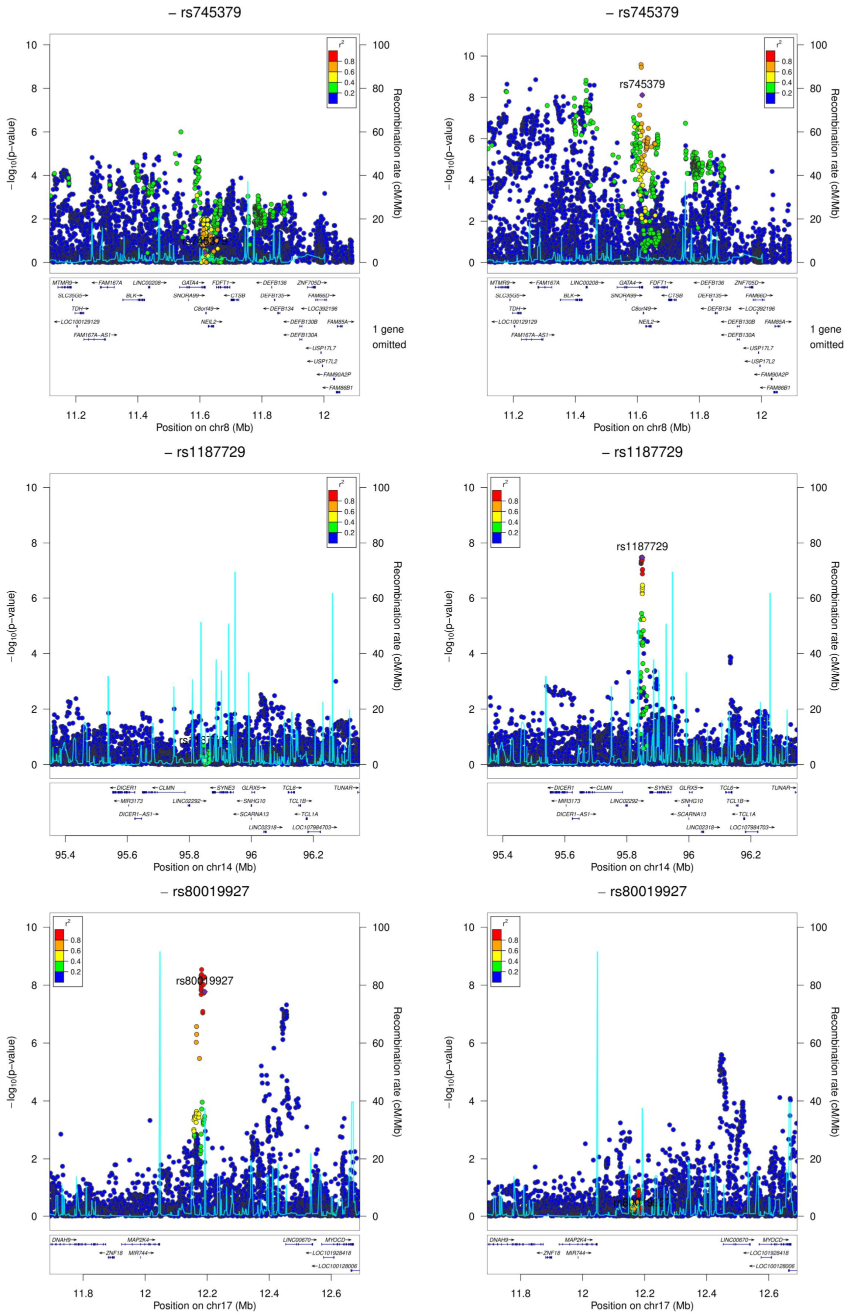

Figure S7. Locus zoom plots for sex specific loci.

The variants with the strongest interaction effect were marked as the reference variant in each plot. Each row shows one locus in females (left) and males (right) respectively.

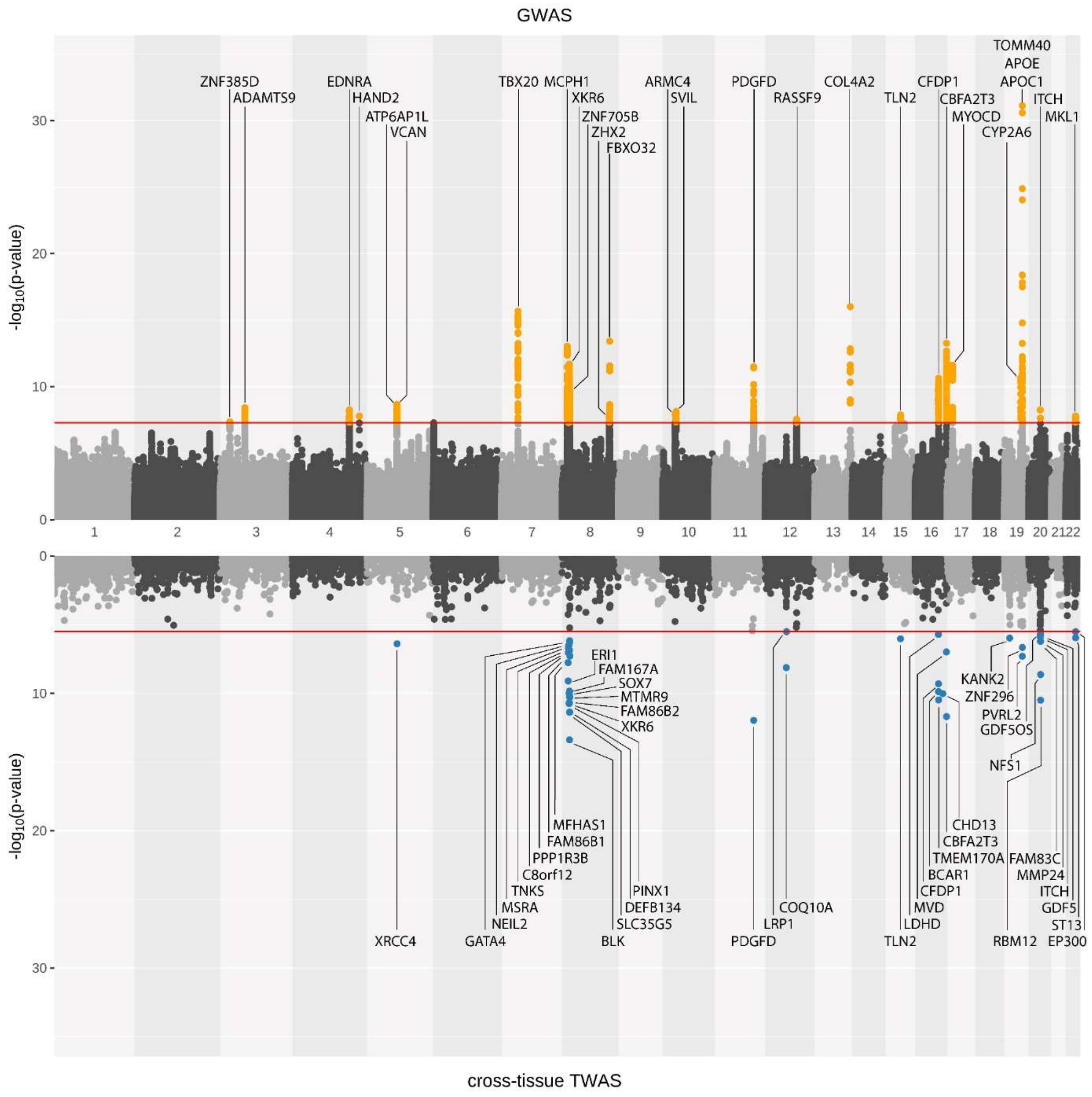

Figure S8. Miami plot of $cIMT_{max}$

The top panel shows the GWAS results while the bottom panel shows results from cross-tissue TWAS. Orange dot: variants passing genome-wide significance threshold ($P<5\times10^{-8}$) in GWAS; Blue dots: genes passing significance threshold after Bonferroni correction ($P<3.3\times10^{-6}$) in TWAS.

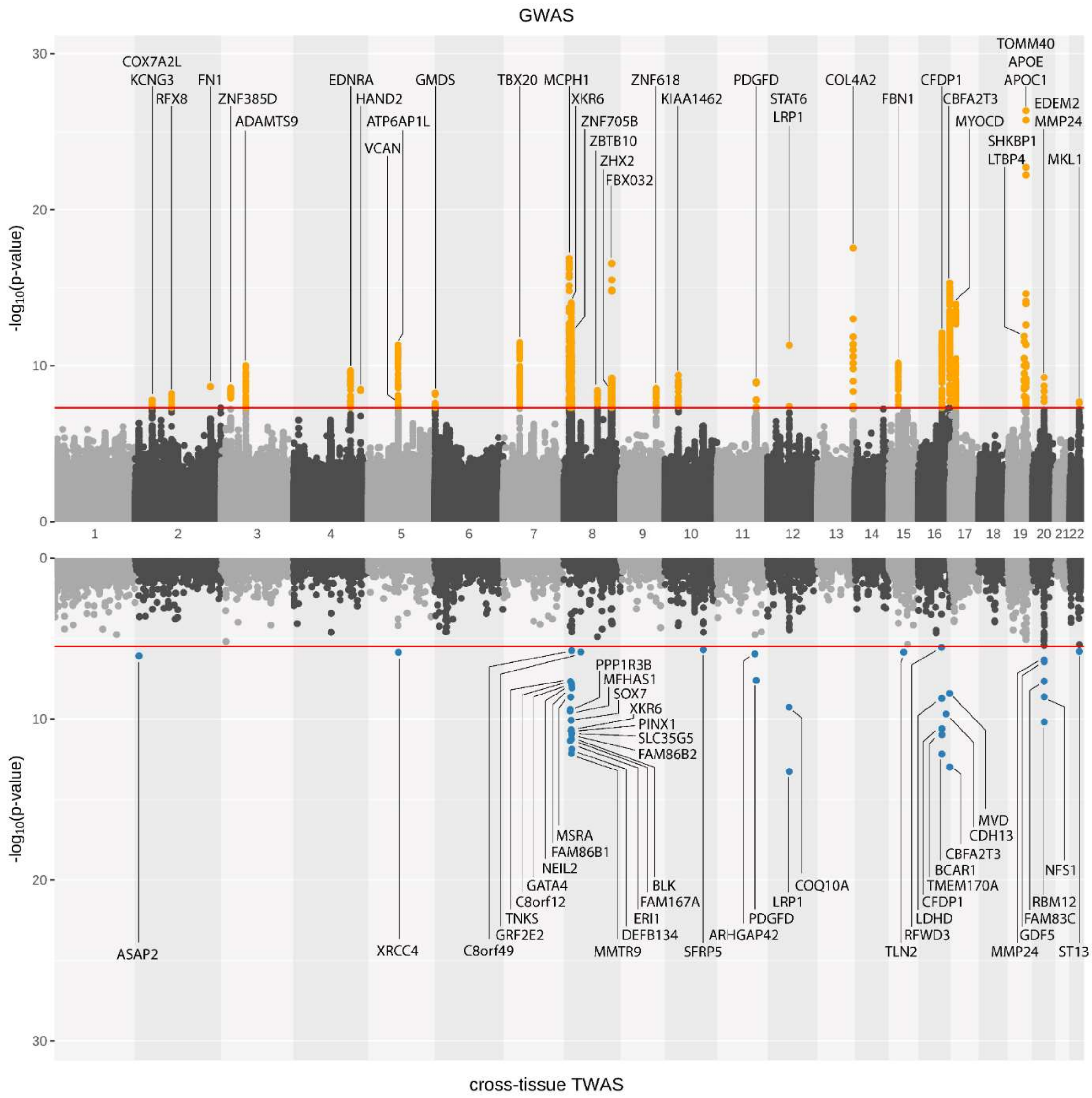

Figure S9. Miami plot of $cIMT_{min}$

The top panel shows the GWAS results while the bottom panel shows results from cross-tissue TWAS. Orange dot: variants passing genome-wide significance threshold (P<5×10^{-8}) in GWAS; Blue dots: genes passing significance threshold after Bonferroni correction (P < 3.3×10^{-6}) in TWAS.

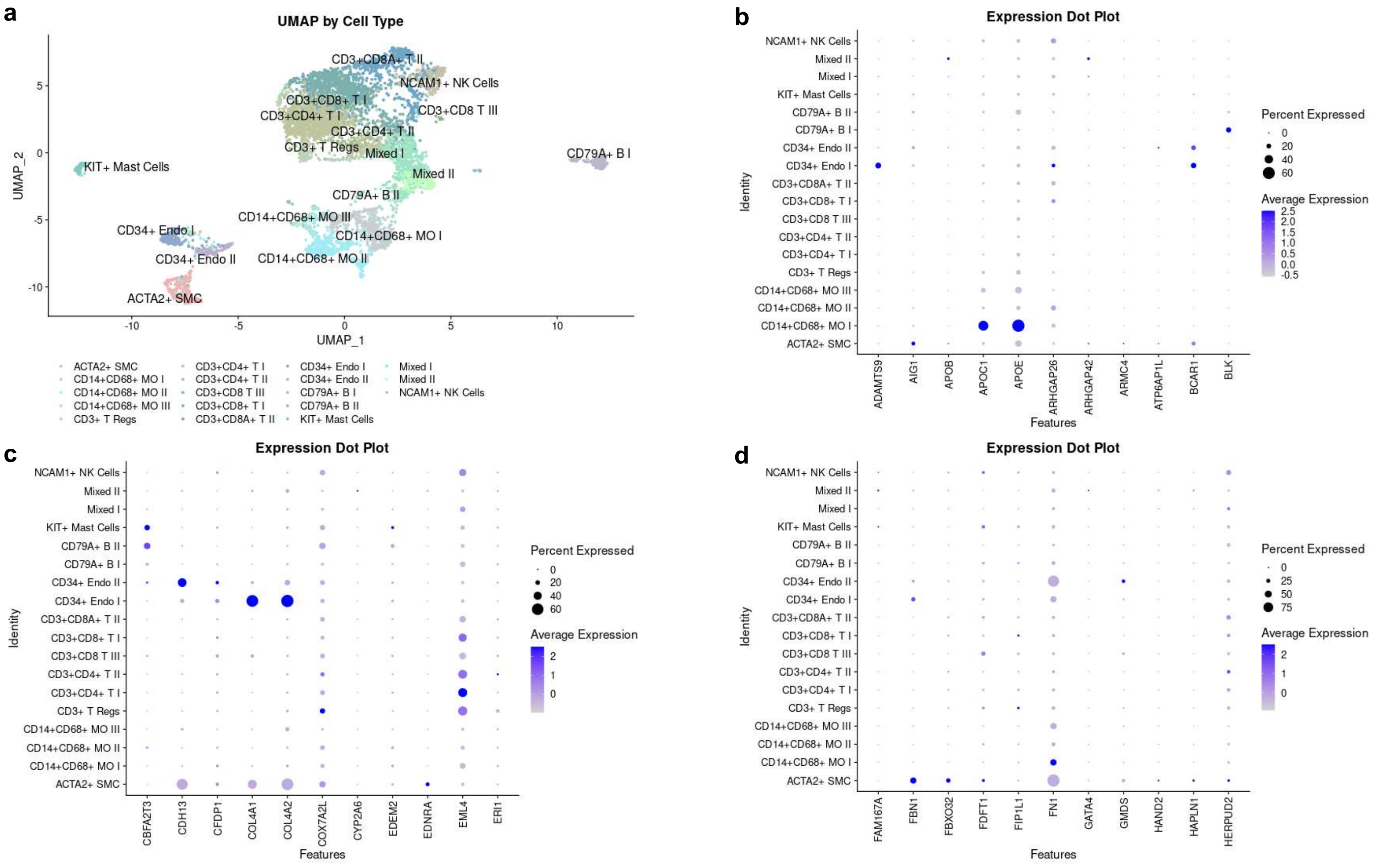
a
UMAP by Cell Type
UMAP_1
UMAP_2
ACTA2+ SMC
CD14+CD68+ MO I
CD14+CD68+ MO II
CD14+CD68+ MO III
CD3+ T Regs
CD3+CD4+ T I
CD3+CD4+ T II
CD3+CD8 T III
CD3+CD8+ T I
CD3+CD8A+ T II
CD34+ Endo I
CD34+ Endo II
CD79A+ B I
CD79A+ B II
KIT+ Mast Cells
Mixed I
Mixed II
NCAM1+ NK Cells
b
Expression Dot Plot
Identity
Features
ADAMTS9
AIG1
APOB
APOC1
APOE
ARHGAP26
ARHGAP42
ARMC4
ATP6AP1L
BCAR1
BLK
Percent Expressed
Average Expression
c
Expression Dot Plot
CBFA2T3
CDH13
CFDP1
COL4A1
COL4A2
COX7A2L
CYP2A6
EDEM2
EDNRA
EML4
ERI1
d
Expression Dot Plot
FAM167A
FBN1
FBXO32
FDFT1
FIP1L1
FN1
GATA4
GMDS
HAND2
HAPLN1
HERPUD2

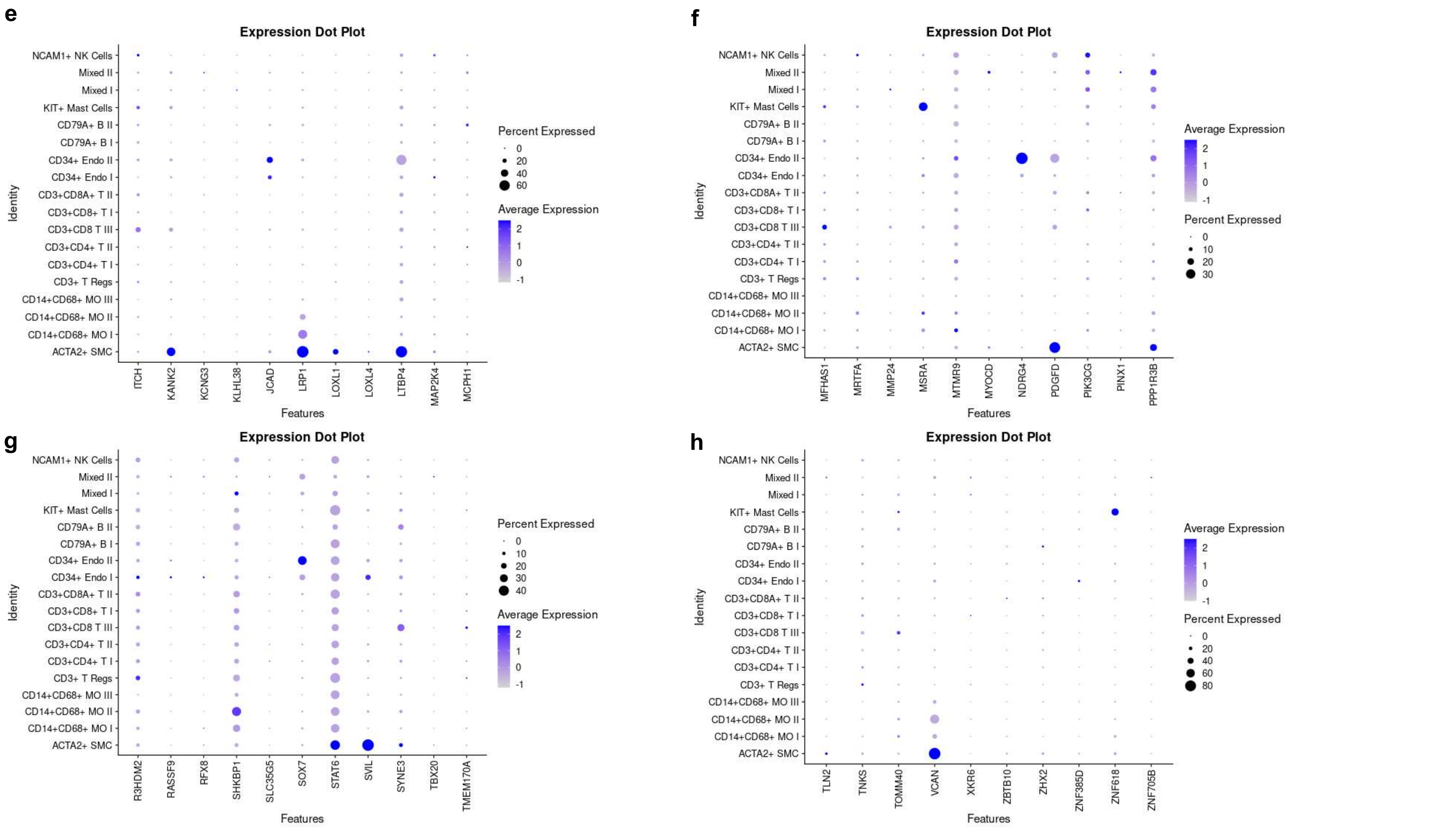

Figure S10. Expression of prioritized genes in human carotid plaque tissues.

a. UMAP clustering plots on cell populations denoted with corresponding identities (determined by marker expressions). b-h: Expression levels of genes, which are the closest genes to GWAS loci or prioritized in subsequent bioinformatic analyses, stratified by cell populations. B: B cells; Endo: endothelial; MO: macrophage; NK: natural killer; SMC: smooth muscle cell; T: T cells; T Regs: regulatory T cells.

Major Resources Table

Data & Code Availability

Description	Source / Repository	Persistent ID / URL
Summary statistics of cIMT GWAS in 45,185 UK Biobank participants	Mendeley	doi: 10.17632/47gd92pbn9.1
UK Biobank data	UK Biobank	Available for researchers upon approved request by the UK Biobank https://www.ukbiobank.ac.uk/
CARDIoGRAMplusC4D summary statistics	CARDIoGRAMplusC4D consortium	http://www.cardiogramplusc4d.org/data-downloads/
CHARGE summary statistics	dbGaP	Available for researchers upon approved request by data access committee from NIH dbGaP Study Accession: phs000930.v9.p1 Analysis Accession: pha004762.1
GERA summary statistics on blood pressure traits	GWAS catalog	Systolic blood pressure: https://www.ebi.ac.uk/gwas/studies/GCST007095 Diastolic blood pressure: https://www.ebi.ac.uk/gwas/studies/GCST007098
GLGC summary statistics	GLGC	http://csg.sph.umich.edu/willer/public/lipids2013/
GTEx summary statistics	GTEx portal	https://gtexportal.org/home/datasets
MEGASTROKE summary statistics	MEGASTROKE consortium	https://www.megastroke.org/download.html
BOLT-LMM		https://alkesgroup.broadinstitute.org/BOLT-LMM/downloads/
dbNSFP		https://sites.google.com/site/jpopgen/dbNSFP
DEPICT		https://data.broadinstitute.org/mpg/depict/
FINEMAP		http://www.christianbenner.com/
FUMA		https://fuma.ctglab.nl/
LDSC	GitHub	https://github.com/bulik/ldsc
LocusZoom		http://locuszoom.org/
MTAG	GitHub	https://github.com/JonJala/mtag
PlaqView	GitHub	https://github.com/MillerLab-CPHG/PlaqView
PLINK 1.9		https://www.cog-genomics.org/plink/
SMR		https://cnsgenomics.com/software/smr/#Overview
UTMOST	GitHub	https://github.com/Joker-Jerome/UTMOST

Supplementary Note

MEGASTROKE CONSORTIUM

Rainer Malik [1], Ganesh Chauhan [2], Matthew Traylor [3], Muralidharan Sargurupremraj [4,5], Yukinori Okada [6,7,8], Aniket Mishra [4,5], Loes Rutten-Jacobs [3], Anne-Katrin Giese [9], Sander W van der Laan [10], Solveig Gretarsdottir [11], Christopher D Anderson [12,13,14,14], Michael Chong [15], Hieab HH Adams [16,17], Tetsuro Ago [18], Peter Almgren [19], Philippe Amouyel [20,21], Hakan Ay [22,13], Traci M Bartz [23], Oscar R Benavente [24], Steve Bevan [25], Giorgio B Boncoraglio [26], Robert D Brown, Jr. [27], Adam S Butterworth [28,29], Caty Carrera [30,31], Cara L Carty [32,33], Daniel I Chasman [34,35], Wei-Min Chen [36], John W Cole [37], Adolfo Correa [38], Ioana Cotlarciuc [39], Carlos Cruchaga [40,41], John Danesh [28,42,43,44], Paul IW de Bakker [45,46], Anita L DeStefano [47,48], Marcel den Hoed [49], Qing Duan [50], Stefan T Engelter [51,52], Guido J Falcone [53,54], Rebecca F Gottesman [55], Raji P Grewal [56], Vilmundur Gudnason [57,58], Stefan Gustafsson [59], Jeffrey Haessler [60], Tamara B Harris [61], Ahamad Hassan [62], Aki S Havulinna [63,64], Susan R Heckbert [65], Elizabeth G Holliday [66,67], George Howard [68], Fang-Chi Hsu [69], Hyacinth I Hyacinth [70], M Arfan Ikram [16], Erik Ingelsson [71,72], Marguerite R Irvin [73], Xueqiu Jian [74], Jordi Jiménez-Conde [75], Julie A Johnson [76,77], J Wouter Jukema [78], Masahiro Kanai [6,7,79], Keith L Keene [80,81], Brett M Kissela [82], Dawn O Kleindorfer [82], Charles Kooperberg [60], Michiaki Kubo [83], Leslie A Lange [84], Carl D Langefeld [85], Claudia Langenberg [86], Lenore J Launer [87], Jin-Moo Lee [88], Robin Lemmens [89,90], Didier Leys [91], Cathryn M Lewis [92,93], Wei-Yu Lin [28,94], Arne G Lindgren [95,96], Erik Lorentzen [97], Patrik K Magnusson [98], Jane Maguire [99], Ani Manichaikul [36], Patrick F McArdle [100], James F Meschia [101], Braxton D Mitchell [100,102], Thomas H Mosley [103,104], Michael A Nalls [105,106], Toshiharu Ninomiya [107], Martin J O'Donnell [15,108], Bruce M Psaty [109,110,111,112], Sara L Pulit [113,45], Kristiina Rannikmäe [114,115], Alexander P Reiner [65,116], Kathryn M Rexrode [117], Kenneth Rice [118], Stephen S Rich [36], Paul M Ridker [34,35], Natalia S Rost [9,13], Peter M Rothwell [119], Jerome I Rotter [120,121], Tatjana Rundek [122], Ralph L Sacco [122], Saori Sakaue [7,123], Michele M Sale [124], Veikko Salomaa [63], Bishwa R Sapkota [125], Reinhold Schmidt [126], Carsten O Schmidt [127], Ulf Schminke [128], Pankaj Sharma [39], Agnieszka Slowik [129], Cathie LM Sudlow [114,115], Christian Tanislav [130], Turgut Tatlisumak [131,132], Kent D Taylor [120,121], Vincent NS Thijs [133,134], Gudmar Thorleifsson [11], Unnur Thorsteinsdottir [11], Steffen Tiedt [1], Stella Trompet [135], Christophe Tzourio [5,136,137], Cornelia M van Duijn [138,139], Matthew Walters [140], Nicholas J Wareham [86], Sylvia Wassertheil-Smoller [141], James G Wilson [142], Kerri L Wiggins [109], Qiong Yang [47], Salim Yusuf [15], Najaf Amin [16], Hugo S Aparicio [185,48], Donna K Arnett [186], John Attia [187], Alexa S Beiser [47,48], Claudine Berr [188], Julie E Buring [34,35], Mariana Bustamante [189], Valeria Caso [190], Yu-Ching Cheng [191], Seung Hoan Choi [192,48], Ayesha Chowhan [185,48], Natalia Cullell [31], Jean-François Dartigues [193,194], Hossein Delavaran [95,96], Pilar Delgado [195], Marcus Dörr [196,197], Gunnar Engström [19], Ian Ford [198], Wander S Gurpreet [199], Anders Hamsten [200,201], Laura Heitsch [202], Atsushi Hozawa [203], Laura Ibanez [204], Andreea Ilinca [95,96], Martin Ingelsson [205], Motoki Iwasaki [206], Rebecca D Jackson [207], Katarina Jood [208], Pekka Jousilahti [63], Sara Kaffashian [4,5], Lalit Kalra [209], Masahiro Kamouchi [210], Takanari Kitazono [211], Olafur Kjartansson [212], Manja Kloss [213], Peter J Koudstaal [214], Jerzy Krupinski [215], Daniel L Labovitz [216], Cathy C Laurie [118], Christopher R Levi [217], Linxin Li [218], Lars Lind [219], Cecilia M Lindgren [220,221], Vasileios Lioutas [222,48], Yong Mei Liu [223], Oscar L Lopez [224], Hirata Makoto [225], Nicolas Martinez-Majander [172], Koichi Matsuda [225], Naoko Minegishi [203], Joan Montaner [226], Andrew P Morris [227,228], Elena Muiño [31], Martina Müller-Nurasyid [229,230,231], Bo Norrving [95,96], Soichi Ogishima [203], Eugenio A Parati [232], Leema Reddy Peddareddygari [56], Nancy L Pedersen [98,233], Joanna Pera [129], Markus Perola [63,234], Alessandro Pezzini [235], Silvana Pileggi [236], Raquel Rabionet [237], Iolanda Riba-Llena [30], Marta Ribasés [238], Jose R Romero [185,48], Jaume Roquer [239,240], Anthony G Rudd [241,242], Antti-Pekka Sarin [243,244], Ralhan Sarju [199], Chloe Sarnowski [47,48], Makoto Sasaki [245], Claudia L Satizabal [185,48], Mamoru Satoh [245], Naveed Sattar [246], Norie Sawada [206], Gerli Sibolt [172], Ásgeir Sigurdsson [247], Albert Smith [248], Kenji Sobue [245], Carolina Soriano-Tárraga [240], Tara Stanne [249], O Colin Stine [250], David J Stott [251], Konstantin Strauch [229,252], Takako Takai [203], Hideo Tanaka [253,254], Kozo Tanno [245], Alexander Teumer [255], Liisa Tomppo [172], Nuria P Torres-Aguila [31], Emmanuel Touze [256,257],

Shoichiro Tsugane [206], Andre G Uitterlinden [258], Einar M Valdimarsson [259], Sven J van der Lee [16], Henry Völzke [255], Kenji Wakai [253], David Weir [260], Stephen R Williams [261], Charles DA Wolfe [241,242], Quenna Wong [118], Huichun Xu [191], Taiki Yamaji [206], Dharambir K Sanghera [125,169,170], Olle Melander [19], Christina Jern [171], Daniel Strbian [172,173], Israel Fernandez-Cadenas [31,30], W T Longstreth, Jr [174,65], Arndt Rolfs [175], Jun Hata [107], Daniel Woo [82], Jonathan Rosand [12,13,14], Guillaume Pare [15], Jemma C Hopewell [176], Danish Saleheen [177], Kari Stefansson [11,178], Bradford B Worrall [179], Steven J Kittner [37], Sudha Seshadri [180,48], Myriam Fornage [74,181], Hugh S Markus [3], Joanna MM Howson [28], Yoichiro Kamatani [6,182], Stephanie Debette [4,5], Martin Dichgans [1,183,184]

1 Institute for Stroke and Dementia Research (ISD), University Hospital, LMU Munich, Munich, Germany
2 Centre for Brain Research, Indian Institute of Science, Bangalore, India
3 Stroke Research Group, Division of Clinical Neurosciences, University of Cambridge, UK
4 INSERM U1219 Bordeaux Population Health Research Center, Bordeaux, France
5 University of Bordeaux, Bordeaux, France
6 Laboratory for Statistical Analysis, RIKEN Center for Integrative Medical Sciences, Yokohama, Japan
7 Department of Statistical Genetics, Osaka University Graduate School of Medicine, Osaka, Japan
8 Laboratory of Statistical Immunology, Immunology Frontier Research Center (WPI-IFReC), Osaka University, Suita, Japan.
9 Department of Neurology, Massachusetts General Hospital, Harvard Medical School, Boston, MA, USA
10 Laboratory of Experimental Cardiology, Division of Heart and Lungs, University Medical Center Utrecht, University of Utrecht, Utrecht,Netherlands
11 deCODE genetics/AMGEN inc, Reykjavik, Iceland
12 Center for Genomic Medicine, Massachusetts General Hospital (MGH), Boston, MA, USA
13 J. Philip Kistler Stroke Research Center, Department of Neurology, MGH, Boston, MA, USA
14 Program in Medical and Population Genetics, Broad Institute, Cambridge, MA, USA
15 Population Health Research Institute, McMaster University, Hamilton, Canada
16 Department of Epidemiology, Erasmus University Medical Center, Rotterdam, Netherlands
17 Department of Radiology and Nuclear Medicine, Erasmus University Medical Center, Rotterdam, Netherlands
18 Department of Medicine and Clinical Science, Graduate School of Medical Sciences, Kyushu University, Fukuoka, Japan
19 Department of Clinical Sciences, Lund University, Malmö, Sweden
20 Univ. Lille, Inserm, Institut Pasteur de Lille, LabEx DISTALZ-UMR1167, Risk factors and molecular determinants of aging-related diseases, F-59000 Lille, France
21 Centre Hosp. Univ Lille, Epidemiology and Public Health Department, F-59000 Lille, France
22 AA Martinos Center for Biomedical Imaging, Department of Radiology, Massachusetts General Hospital, Harvard Medical School, Boston, MA, USA
23 Cardiovascular Health Research Unit, Departments of Biostatistics and Medicine, University of Washington, Seattle, WA, USA
24 Division of Neurology, Faculty of Medicine, Brain Research Center, University of British Columbia, Vancouver, Canada
25 School of Life Science, University of Lincoln, Lincoln, UK
26 Department of Cerebrovascular Diseases, Fondazione IRCCS Istituto Neurologico "Carlo Besta", Milano, Italy
27 Department of Neurology, Mayo Clinic Rochester, Rochester, MN, USA
28 MRC/BHF Cardiovascular Epidemiology Unit, Department of Public Health and Primary Care, University of Cambridge, Cambridge, UK

29 The National Institute for Health Research Blood and Transplant Research Unit in Donor Health and Genomics, University of Cambridge, UK
30 Neurovascular Research Laboratory, Vall d'Hebron Institut of Research, Neurology and Medicine Departments-Universitat Autònoma de Barcelona, Vall d’Hebrón Hospital, Barcelona, Spain
31 Stroke Pharmacogenomics and Genetics, Fundacio Docència i Recerca MutuaTerrassa, Terrassa, Spain
32 Children's Research Institute, Children's National Medical Center, Washington, DC, USA
33 Center for Translational Science, George Washington University, Washington, DC, USA
34 Division of Preventive Medicine, Brigham and Women's Hospital, Boston, MA, USA
35 Harvard Medical School, Boston, MA, USA
36 Center for Public Health Genomics, Department of Public Health Sciences, University of Virginia, Charlottesville, VA, USA
37 Department of Neurology, University of Maryland School of Medicine and Baltimore VAMC, Baltimore, MD, USA
38 Departments of Medicine, Pediatrics and Population Health Science, University of Mississippi Medical Center, Jackson, MS, USA
39 Institute of Cardiovascular Research, Royal Holloway University of London, UK & Ashford and St Peters Hospital, Surrey UK
40 Department of Psychiatry,The Hope Center Program on Protein Aggregation and Neurodegeneration (HPAN),Washington University, School of Medicine, St. Louis, MO, USA
41 Department of Developmental Biology, Washington University School of Medicine, St. Louis, MO, USA
42 NIHR Blood and Transplant Research Unit in Donor Health and Genomics, Department of Public Health and Primary Care, University of Cambridge, Cambridge, UK
43 Wellcome Trust Sanger Institute, Wellcome Trust Genome Campus, Hinxton, Cambridge, UK
44 British Heart Foundation, Cambridge Centre of Excellence, Department of Medicine, University of Cambridge, Cambridge, UK
45 Department of Medical Genetics, University Medical Center Utrecht, Utrecht, Netherlands
46 Department of Epidemiology, Julius Center for Health Sciences and Primary Care, University Medical Center Utrecht, Utrecht, Netherlands
47 Boston University School of Public Health, Boston, MA, USA
48 Framingham Heart Study, Framingham, MA, USA
49 Department of Immunology, Genetics and Pathology and Science for Life Laboratory, Uppsala University, Uppsala, Sweden
50 Department of Genetics, University of North Carolina, Chapel Hill, NC, USA
51 Department of Neurology and Stroke Center, Basel University Hospital, Switzerland
52 Neurorehabilitation Unit, University and University Center for Medicine of Aging and Rehabilitation Basel, Felix Platter Hospital, Basel, Switzerland
53 Department of Neurology, Yale University School of Medicine, New Haven, CT, USA
54 Program in Medical and Population Genetics, The Broad Institute of Harvard and MIT, Cambridge, MA, USA
55 Department of Neurology, Johns Hopkins University School of Medicine, Baltimore, MD, USA
56 Neuroscience Institute, SF Medical Center, Trenton, NJ, USA
57 Icelandic Heart Association Research Institute, Kopavogur, Iceland
58 University of Iceland, Faculty of Medicine, Reykjavik, Iceland
59 Department of Medical Sciences, Molecular Epidemiology and Science for Life Laboratory, Uppsala University, Uppsala, Sweden
60 Division of Public Health Sciences, Fred Hutchinson Cancer Research Center, Seattle, WA, USA
61 Laboratory of Epidemiology and Population Science, National Institute on Aging, National Institutes of Health, Bethesda, MD, USA

62 Department of Neurology, Leeds General Infirmary, Leeds Teaching Hospitals NHS Trust, Leeds, UK
63 National Institute for Health and Welfare, Helsinki, Finland
64 FIMM - Institute for Molecular Medicine Finland, Helsinki, Finland
65 Department of Epidemiology, University of Washington, Seattle, WA, USA
66 Public Health Stream, Hunter Medical Research Institute, New Lambton, Australia
67 Faculty of Health and Medicine, University of Newcastle, Newcastle, Australia
68 School of Public Health, University of Alabama at Birmingham, Birmingham, AL, USA
69 Department of Biostatistical Sciences, Wake Forest School of Medicine, Winston-Salem, NC, USA
70 Aflac Cancer and Blood Disorder Center, Department of Pediatrics, Emory University School of Medicine, Atlanta, GA, USA
71 Department of Medicine, Division of Cardiovascular Medicine, Stanford University School of Medicine, CA, USA
72 Department of Medical Sciences, Molecular Epidemiology and Science for Life Laboratory, Uppsala University, Uppsala, Sweden
73 Epidemiology, School of Public Health, University of Alabama at Birmingham, USA
74 Brown Foundation Institute of Molecular Medicine, University of Texas Health Science Center at Houston, Houston, TX, USA
75 Neurovascular Research Group (NEUVAS), Neurology Department, Institut Hospital del Mar d'Investigació Mèdica, Universitat Autònoma de Barcelona, Barcelona, Spain
76 Department of Pharmacotherapy and Translational Research and Center for Pharmacogenomics, University of Florida, College of Pharmacy, Gainesville, FL, USA
77 Division of Cardiovascular Medicine, College of Medicine, University of Florida, Gainesville, FL, USA
78 Department of Cardiology, Leiden University Medical Center, Leiden, the Netherlands
79 Program in Bioinformatics and Integrative Genomics, Harvard Medical School, Boston, MA, USA
80 Department of Biology, East Carolina University, Greenville, NC, USA
81 Center for Health Disparities, East Carolina University, Greenville, NC, USA
82 University of Cincinnati College of Medicine, Cincinnati, OH, USA
83 RIKEN Center for Integrative Medical Sciences, Yokohama, Japan
84 Department of Medicine, University of Colorado Denver, Anschutz Medical Campus, Aurora, CO, USA
85 Center for Public Health Genomics and Department of Biostatistical Sciences, Wake Forest School of Medicine, Winston-Salem, NC, USA
86 MRC Epidemiology Unit, University of Cambridge School of Clinical Medicine, Institute of Metabolic Science, Cambridge Biomedical Campus, Cambridge, UK
87 Intramural Research Program, National Institute on Aging, National Institutes of Health, Bethesda, MD, USA
88 Department of Neurology, Radiology, and Biomedical Engineering, Washington University School of Medicine, St. Louis, MO, USA
89 KU Leuven – University of Leuven, Department of Neurosciences, Experimental Neurology, Leuven, Belgium
90 VIB Center for Brain & Disease Research, University Hospitals Leuven, Department of Neurology, Leuven, Belgium
91 Univ.-Lille, INSERM U 1171. CHU Lille. Lille, France
92 Department of Medical and Molecular Genetics, King's College London, London, UK
93 SGDP Centre, Institute of Psychiatry, Psychology & Neuroscience, King's College London, London, UK
94 Northern Institute for Cancer Research, Paul O'Gorman Building, Newcastle University, Newcastle, UK
95 Department of Clinical Sciences Lund, Neurology, Lund University, Lund, Sweden
96 Department of Neurology and Rehabilitation Medicine, Skåne University Hospital, Lund, Sweden
97 Bioinformatics Core Facility, University of Gothenburg, Gothenburg, Sweden
98 Department of Medical Epidemiology and Biostatistics, Karolinska Institutet, Stockholm, Sweden

99 University of Technology Sydney, Faculty of Health, Ultimo, Australia
100 Department of Medicine, University of Maryland School of Medicine, MD, USA
101 Department of Neurology, Mayo Clinic, Jacksonville, FL, USA
102 Geriatrics Research and Education Clinical Center, Baltimore Veterans Administration Medical Center, Baltimore, MD, USA
103 Division of Geriatrics, School of Medicine, University of Mississippi Medical Center, Jackson, MS, USA
104 Memory Impairment and Neurodegenerative Dementia Center, University of Mississippi Medical Center, Jackson, MS, USA
105 Laboratory of Neurogenetics, National Institute on Aging, National institutes of Health, Bethesda, MD, USA
106 Data Tecnica International, Glen Echo MD, USA
107 Department of Epidemiology and Public Health, Graduate School of Medical Sciences, Kyushu University, Fukuoka, Japan
108 Clinical Research Facility, Department of Medicine, NUI Galway, Galway, Ireland
109 Cardiovascular Health Research Unit, Department of Medicine, University of Washington, Seattle, WA, USA
110 Department of Epidemiology, University of Washington, Seattle, WA
111 Department of Health Services, University of Washington, Seattle, WA, USA
112 Kaiser Permanente Washington Health Research Institute, Seattle, WA, USA
113 Brain Center Rudolf Magnus, Department of Neurology, University Medical Center Utrecht, Utrecht, The Netherlands
114 Usher Institute of Population Health Sciences and Informatics, University of Edinburgh, Edinburgh, UK
115 Centre for Clinical Brain Sciences, University of Edinburgh, Edinburgh, UK
116 Fred Hutchinson Cancer Research Center, University of Washington, Seattle, WA, USA
117 Department of Medicine, Brigham and Women's Hospital, Boston, MA, USA
118 Department of Biostatistics, University of Washington, Seattle, WA, USA
119 Nuffield Department of Clinical Neurosciences, University of Oxford, UK
120 Institute for Translational Genomics and Population Sciences, Los Angeles Biomedical Research Institute at Harbor-UCLA Medical Center, Torrance, CA, USA
121 Division of Genomic Outcomes, Department of Pediatrics, Harbor-UCLA Medical Center, Torrance, CA, USA
122 Department of Neurology, Miller School of Medicine, University of Miami, Miami, FL, USA
123 Department of Allergy and Rheumatology, Graduate School of Medicine, the University of Tokyo, Tokyo, Japan
124 Center for Public Health Genomics, University of Virginia, Charlottesville, VA, USA
125 Department of Pediatrics, College of Medicine, University of Oklahoma Health Sciences Center, Oklahoma City, OK, USA
126 Department of Neurology, Medical University of Graz, Graz, Austria
127 University Medicine Greifswald, Institute for Community Medicine, SHIP-KEF, Greifswald, Germany
128 University Medicine Greifswald, Department of Neurology, Greifswald, Germany
129 Department of Neurology, Jagiellonian University, Krakow, Poland
130 Department of Neurology, Justus Liebig University, Giessen, Germany
131 Department of Clinical Neurosciences/Neurology, Institute of Neuroscience and Physiology, Sahlgrenska Academy at University of Gothenburg, Gothenburg, Sweden
132 Sahlgrenska University Hospital, Gothenburg, Sweden
133 Stroke Division, Florey Institute of Neuroscience and Mental Health, University of Melbourne, Heidelberg, Australia
134 Austin Health, Department of Neurology, Heidelberg, Australia
135 Department of Internal Medicine, Section Gerontology and Geriatrics, Leiden University Medical Center, Leiden, the Netherlands
136 INSERM U1219, Bordeaux, France
137 Department of Public Health, Bordeaux University Hospital, Bordeaux, France

138 Genetic Epidemiology Unit, Department of Epidemiology, Erasmus University Medical Center Rotterdam, Netherlands
139 Center for Medical Systems Biology, Leiden, Netherlands
140 School of Medicine, Dentistry and Nursing at the University of Glasgow, Glasgow, UK
141 Department of Epidemiology and Population Health, Albert Einstein College of Medicine, NY, USA
142 Department of Physiology and Biophysics, University of Mississippi Medical Center, Jackson, MS, USA
143 A full list of members and affiliations appears in the Supplementary Note
144 Department of Human Genetics, McGill University, Montreal, Canada
145 Department of Pathophysiology, Institute of Biomedicine and Translation Medicine, University of Tartu, Tartu, Estonia
146 Department of Cardiac Surgery, Tartu University Hospital, Tartu, Estonia
147 Clinical Gene Networks AB,Stockholm, Sweden
148 Department of Genetics and Genomic Sciences, The Icahn Institute for Genomics and Multiscale Biology Icahn School of Medicine at Mount Sinai, New York, NY , USA
149 Department of Pathophysiology, Institute of Biomedicine and Translation Medicine, University of Tartu, Biomeedikum, Tartu, Estonia
150 Integrated Cardio Metabolic Centre, Department of Medicine, Karolinska Institutet, Karolinska Universitetssjukhuset, Huddinge, Sweden.
151 Clinical Gene Networks AB, Stockholm, Sweden
152 Sorbonne Universités, UPMC Univ. Paris 06, INSERM, UMR_S 1166, Team Genomics & Pathophysiology of Cardiovascular Diseases, Paris, France
153 ICAN Institute for Cardiometabolism and Nutrition, Paris, France
154 Department of Biomedical Engineering, University of Virginia, Charlottesville, VA, USA
155 Group Health Research Institute, Group Health Cooperative, Seattle, WA, USA
156 Seattle Epidemiologic Research and Information Center, VA Office of Research and Development, Seattle, WA, USA
157 Cardiovascular Research Center, Massachusetts General Hospital, Boston, MA, USA
158 Department of Medical Research, Bærum Hospital, Vestre Viken Hospital Trust, Gjettum, Norway
159 Saw Swee Hock School of Public Health, National University of Singapore and National University Health System, Singapore
160 National Heart and Lung Institute, Imperial College London, London, UK
161 Department of Gene Diagnostics and Therapeutics, Research Institute, National Center for Global Health and Medicine, Tokyo, Japan
162 Department of Epidemiology, Tulane University School of Public Health and Tropical Medicine, New Orleans, LA, USA
163 Department of Cardiology,University Medical Center Groningen, University of Groningen, Netherlands
164 MRC-PHE Centre for Environment and Health, School of Public Health, Department of Epidemiology and Biostatistics, Imperial College London, London, UK
165 Department of Epidemiology and Biostatistics, Imperial College London, London, UK
166 Department of Cardiology, Ealing Hospital NHS Trust, Southall, UK
167 National Heart, Lung and Blood Research Institute, Division of Intramural Research, Population Sciences Branch, Framingham, MA, USA
168 A full list of members and affiliations appears at the end of the manuscript
169 Department of Phamaceutical Sciences, Collge of Pharmacy, University of Oklahoma Health Sciences Center, Oklahoma City, OK, USA
170 Oklahoma Center for Neuroscience, Oklahoma City, OK, USA
171 Department of Pathology and Genetics, Institute of Biomedicine, The Sahlgrenska Academy at University of Gothenburg, Gothenburg, Sweden
172 Department of Neurology, Helsinki University Hospital, Helsinki, Finland
173 Clinical Neurosciences, Neurology, University of Helsinki, Helsinki, Finland
174 Department of Neurology, University of Washington, Seattle, WA, USA
175 Albrecht Kossel Institute, University Clinic of Rostock, Rostock, Germany

176 Clinical Trial Service Unit and Epidemiological Studies Unit, Nuffield Department of Population Health, University of Oxford, Oxford, UK
177 Department of Genetics, Perelman School of Medicine, University of Pennsylvania, PA, USA
178 Faculty of Medicine, University of Iceland, Reykjavik, Iceland
179 Departments of Neurology and Public Health Sciences, University of Virginia School of Medicine, Charlottesville, VA, USA
180 Department of Neurology, Boston University School of Medicine, Boston, MA, USA
181 Human Genetics Center, University of Texas Health Science Center at Houston, Houston, TX, USA
182 Center for Genomic Medicine, Kyoto University Graduate School of Medicine, Kyoto, Japan
183 Munich Cluster for Systems Neurology (SyNergy), Munich, Germany
184 German Center for Neurodegenerative Diseases (DZNE), Munich, Germany
185 Boston University School of Medicine, Boston, MA, USA
186 University of Kentucky College of Public Health, Lexington, KY, USA
187 University of Newcastle and Hunter Medical Research Institute, New Lambton, Australia
188 Univ. Montpellier, Inserm, U1061, Montpellier, France
189 Centre for Research in Environmental Epidemiology, Barcelona, Spain
190 Department of Neurology, Università degli Studi di Perugia, Umbria, Italy
191 Department of Medicine, University of Maryland School of Medicine, Baltimore, MD, USA
192 Broad Institute, Cambridge, MA, USA
193 Univ. Bordeaux, Inserm, Bordeaux Population Health Research Center, UMR 1219, Bordeaux, France
194 Bordeaux University Hospital, Department of Neurology, Memory Clinic, Bordeaux, France
195 Neurovascular Research Laboratory. Vall d'Hebron Institut of Research, Neurology and Medicine Departments-Universitat Autònoma de Barcelona. Vall d'Hebrón Hospital, Barcelona, Spain
196 University Medicine Greifswald, Department of Internal Medicine B, Greifswald, Germany
197 DZHK, Greifswald, Germany
198 Robertson Center for Biostatistics, University of Glasgow, Glasgow, UK
199 Hero DMC Heart Institute, Dayanand Medical College & Hospital, Ludhiana, India
200 Atherosclerosis Research Unit, Department of Medicine Solna, Karolinska Institutet, Stockholm, Sweden
201 Karolinska Institutet, Stockholm, Sweden
202 Division of Emergency Medicine, and Department of Neurology, Washington University School of Medicine, St. Louis, MO, USA
203 Tohoku Medical Megabank Organization, Sendai, Japan
204 Department of Psychiatry, Washington University School of Medicine, St. Louis, MO, USA
205 Department of Public Health and Caring Sciences / Geriatrics, Uppsala University, Uppsala, Sweden
206 Epidemiology and Prevention Group, Center for Public Health Sciences, National Cancer Center, Tokyo, Japan
207 Department of Internal Medicine and the Center for Clinical and Translational Science, The Ohio State University, Columbus, OH, USA
208 Institute of Neuroscience and Physiology, the Sahlgrenska Academy at University of Gothenburg, Goteborg, Sweden
209 Department of Basic and Clinical Neurosciences, King's College London, London, UK
210 Department of Health Care Administration and Management, Graduate School of Medical Sciences, Kyushu University, Japan
211 Department of Medicine and Clinical Science, Graduate School of Medical Sciences, Kyushu University, Japan

212 Landspitali National University Hospital, Departments of Neurology & Radiology, Reykjavik, Iceland
213 Department of Neurology, Heidelberg University Hospital, Germany
214 Department of Neurology, Erasmus University Medical Center
215 Hospital Universitari Mutua Terrassa, Terrassa (Barcelona), Spain
216 Albert Einstein College of Medicine, Montefiore Medical Center, New York, NY, USA
217 John Hunter Hospital, Hunter Medical Research Institute and University of Newcastle, Newcastle, NSW, Australia
218 Centre for Prevention of Stroke and Dementia, Nuffield Department of Clinical Neurosciences, University of Oxford, UK
219 Department of Medical Sciences, Uppsala University, Uppsala, Sweden
220 Genetic and Genomic Epidemiology Unit, Wellcome Trust Centre for Human Genetics, University of Oxford, Oxford, UK
221 The Wellcome Trust Centre for Human Genetics, Oxford, UK
222 Beth Israel Deaconess Medical Center, Boston, MA, USA
223 Wake Forest School of Medicine, Wake Forest, NC, USA
224 Department of Neurology, University of Pittsburgh, Pittsburgh, PA, USA
225 BioBank Japan, Laboratory of Clinical Sequencing, Department of Computational biology and medical Sciences, Graduate school of Frontier Sciences, The University of Tokyo, Tokyo, Japan
226 Neurovascular Research Laboratory, Vall d'Hebron Institut of Research, Neurology and Medicine Departments-Universitat Autònoma de Barcelona. Vall d'Hebrón Hospital, Barcelona, Spain
227 Department of Biostatistics, University of Liverpool, Liverpool, UK
228 Wellcome Trust Centre for Human Genetics, University of Oxford, Oxford, UK
229 Institute of Genetic Epidemiology, Helmholtz Zentrum München - German Research Center for Environmental Health, Neuherberg, Germany
230 Department of Medicine I, Ludwig-Maximilians-Universität, Munich, Germany
231 DZHK (German Centre for Cardiovascular Research), partner site Munich Heart Alliance, Munich, Germany
232 Department of Cerebrovascular Diseases, Fondazione IRCCS Istituto Neurologico "Carlo Besta", Milano, Italy
233 Karolinska Institutet, MEB, Stockholm, Sweden
234 University of Tartu, Estonian Genome Center, Tartu, Estonia, Tartu, Estonia
235 Department of Clinical and Experimental Sciences, Neurology Clinic, University of Brescia, Italy
236 Translational Genomics Unit, Department of Oncology, IRCCS Istituto di Ricerche Farmacologiche Mario Negri, Milano, Italy
237 Department of Genetics, Microbiology and Statistics, University of Barcelona, Barcelona, Spain
238 Psychiatric Genetics Unit, Group of Psychiatry, Mental Health and Addictions, Vall d'Hebron Research Institute (VHIR), Universitat Autònoma de Barcelona, Biomedical Network Research Centre on Mental Health (CIBERSAM), Barcelona, Spain
239 Department of Neurology, IMIM-Hospital del Mar, and Universitat Autònoma de Barcelona, Spain
240 IMIM (Hospital del Mar Medical Research Institute), Barcelona, Spain
241 National Institute for Health Research Comprehensive Biomedical Research Centre, Guy's & St. Thomas' NHS Foundation Trust and King's College London, London, UK
242 Division of Health and Social Care Research, King's College London, London, UK
243 FIMM-Institute for Molecular Medicine Finland, Helsinki, Finland
244 THL-National Institute for Health and Welfare, Helsinki, Finland
245 Iwate Tohoku Medical Megabank Organization, Iwate Medical University, Iwate, Japan
246 BHF Glasgow Cardiovascular Research Centre, Faculty of Medicine, Glasgow, UK
247 deCODE Genetics/Amgen, Inc., Reykjavik, Iceland
248 Icelandic Heart Association, Reykjavik, Iceland
249 Institute of Biomedicine, the Sahlgrenska Academy at University of Gothenburg, Goteborg, Sweden

250 Department of Epidemiology, University of Maryland School of Medicine, Baltimore, MD, USA
251 Institute of Cardiovascular and Medical Sciences, Faculty of Medicine, University of Glasgow, Glasgow, UK
252 Chair of Genetic Epidemiology, IBE, Faculty of Medicine, LMU Munich, Germany
253 Division of Epidemiology and Prevention, Aichi Cancer Center Research Institute, Nagoya, Japan
254 Department of Epidemiology, Nagoya University Graduate School of Medicine, Nagoya, Japan
255 University Medicine Greifswald, Institute for Community Medicine, SHIP-KEF, Greifswald, Germany
256 Department of Neurology, Caen University Hospital, Caen, France
257 University of Caen Normandy, Caen, France
258 Department of Internal Medicine, Erasmus University Medical Center, Rotterdam, Netherlands
259 Landspitali University Hospital, Reykjavik, Iceland
260 Survey Research Center, University of Michigan, Ann Arbor, MI, USA
261 University of Virginia Department of Neurology, Charlottesville, VA, US

Reference

121. Fry D, Almond R, Moffat S, Gordon M, Singh P. UK Biobank Biomarker Project Companion Document to Accompany Serum Biomarker Data. Published online 2019. Accessed August 5, 2021. http://www.ukbiobank.ac.uk/uk-biobank-biomarker-panel/.

122. UK Biobank Blood Pressure. Accessed July 31, 2021. http://www.ukbiobank.ac.uk/

123. Zhu Z, Zhang F, Hu H, Bakshi A, Robinson MR, Powell JE, Montgomery GW, Goddard ME, Wray NR, Visscher PM, et al. Integration of summary data from GWAS and eQTL studies predicts complex trait gene targets. *Nat Genet*. 2016;48:481-487. doi:10.1038/ng.3538

124. Ma WF, Hodonsky CJ, Turner AW, Wong D, Song Y, Barrientos NB, Mosquera JV, Miller CL. Single-cell RNA-seq analysis of human coronary arteries using an enhanced workflow reveals SMC transitions and candidate drug targets. *bioRxiv*. Published online March 17, 2021:2020.10.27.357715. doi:10.1101/2020.10.27.357715

125. Pers TH, Karjalainen JM, Chan Y, Westra HJ, Wood AR, Yang J, Lui JC, Vedantam S, Gustafsson S, Esko T, et al. Biological interpretation of genome-wide association studies using predicted gene functions. *Nat Commun*. 2015;6:5890. doi:10.1038/ncomms6890
